# Dynamic control of electron correlations in photodoped charge-transfer insulators

**DOI:** 10.1126/sciadv.adx5676

**Published:** 2025-09-05

**Authors:** Thomas C. Rossi, Nicolas Tancogne-Dejean, Malte Oppermann, Michael Porer, Arnaud Magrez, Rajesh V. Chopdekar, Yayoi Takamura, Urs Staub, Renske M. van der Veen, Angel Rubio, Majed Chergui

**Affiliations:** ^1^ Laboratory of Ultrafast Spectroscopy, SB-ISIC, and Lausanne Centre for Ultrafast Science (LACUS), Ecole Polytechnique Fédérale de Lausanne, Station 6, CH-1015 Lausanne, Switzerland.; ^2^ Department of Atomic-Scale Dynamics in Light-Energy Conversion (PS-ADLU), Helmholtz Zentrum Berlin für Materialien und Energie GmbH, Magnusstrasse 2, 12489 Berlin, Germany.; ^3^ Max Planck Institute for the Structure and Dynamics of Matter, Luruper Chaussee 149, Geb. 99 (CFEL), 22761 Hamburg, Germany.; ^4^ Department of Chemistry, University of Basel, Klingelbergstrasse 80, 4056 Basel, Switzerland.; ^5^ Swiss Light Source, Paul Scherrer Institute, CH-5232 Villigen PSI, Switzerland.; ^6^ Crystal Growth Facility, Institute of Physics, Ecole Polytechnique Fédérale de Lausanne, Lausanne 1015, Switzerland.; ^7^ Department of Materials and Science Engineering, University of California, Davis, One Shields Avenue, Davis, CA 95616, USA.; ^8^ Initiative for Computational Catalysis (ICC) and Center for Computational Quantum Physics (CCQ), Simons Foundation Flatiron Institute, New York, NY 10010, USA.; ^9^ Elettra-Sincrotrone Trieste, SS 14, km 163.5, 34149 Basovizza, Trieste, Italy.

## Abstract

The electronic properties of correlated insulators are governed by the strength of Coulomb interactions, enabling the control of electronic conductivity with external stimuli. This work highlights that the strength of electronic correlations in nickel oxide (NiO), a prototypical charge-transfer insulator, can be coherently reduced by tuning the intensity of an optical pulse excitation. This weakening of correlations persists for hundreds of picoseconds and exhibits a recovery time independent of photodoping density across two orders of magnitude. A broadening of the charge-transfer gap is also observed, consistent with dynamical screening. The high degree of control achieved over both the energy and temporal dynamics of electronic correlations offers a promising avenue to a full optical control of correlated systems and the Mott transition.

## Teaser

Electronic properties of NiO can be optically controlled for hundreds of picoseconds by screening electronic correlations.

## INTRODUCTION

**Fig. 1.**
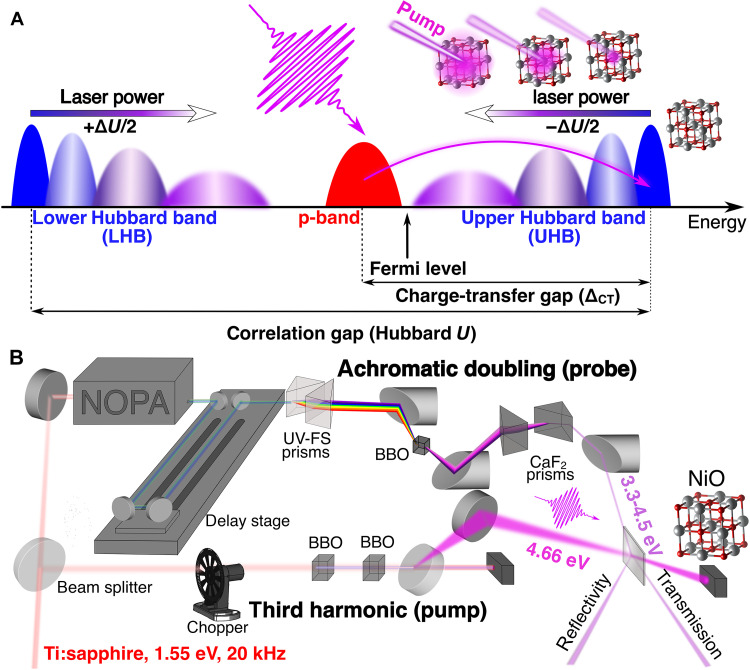
Control over the NiO electronic correlations achieved with a pump-probe experimental setup in the UV. (**A**) Optical control of the correlation gap (Hubbard *U*) in the density of states of NiO films. Photodoping leads to a linear reduction of *U* with the LHB and the UHB shifting in energy by +ΔU/2 and −ΔU/2 , respectively. (**B**) Broadband UV setup to measure the response of NiO to optical excitation above the CT gap. UV-FS, UV-fused silica; BBO, β-barium borate; NOPA, noncolinear optical parametric amplifier.

Transition metal oxides are fascinating because of their wide range of electronic, magnetic, and structural properties, often driven by strong electron correlations. Understanding insulating phases in materials with partially filled d orbitals has been a challenge for decades, with several questions still open to this day. Charge-transfer (CT) insulators have a bandgap $\Delta_{\text{CT}}$ forming between a p-band derived from intermetallic ligand states and a split-off band [upper Hubbard band (UHB)] originating from strong electronic correlations in the metallic 3d orbitals (Fig. 1A). Photodoping upon light excitation across the CT gap has the ability to reduce the strength of on-site Coulomb repulsions (*1*) governing the energy gap between the lower Hubbard band (LHB) and the UHB, referred to as Hubbard *U* (Fig. 1A). Future optoelectronic devices will demand a high degree of control over the electronic properties of correlated materials, which we aim at demonstrating in this work using nickel oxide (NiO) as an example.

Photodoping of CT insulators has been an intense research topic for triggering metal-to-insulator transitions (MITs) or generating new material phases (*2*–*5*). Although ultrafast optical spectroscopy has been listed as one of the most important techniques to study the effect of photodoping on the electronic structure of emerging functional strongly correlated materials (*6*, *7*), there is a lack of studies on CT insulators, which calls for a deeper understanding on the effect of photodoping on electronic correlations. Beyond this, a coherent control of the band structure of a strongly correlated material still awaits to be demonstrated and used.

On the basis of theoretical predictions (*1*, *8*), a few recent works have revealed photoinduced energy renormalization of the UHB in hole-doped cuprates (*9*) and in NiO (*10*) compatible with the screening of electronic correlations. However, these experiments do not capture the dynamical evolution of the bandgap and its dependence with the excitation density, with limited insight into the Coulomb screening in photodoped CT insulators. In this work, we look at how the temporal evolution of the CT gap builds up in NiO by implementing pump-probe optical spectroscopy in the ultraviolet (UV) illustrated in Fig. 1B (*11*, *12*), which reveals the magnitude of the screening of electron correlations and its lifetime. The analysis of spectral line shapes from the broadband UV achromatic doubling combined with spectroscopic ellipsometry demonstrates that a change of screening can be coherently adjusted by controlling the density of photoexcited carriers, exemplifying a clear light-induced control over band-structure renormalization. The CT gap of photoexcited NiO above its bandgap decreases linearly with the photodoping density over two orders of magnitude on a subpicosecond timescale (sketched in Fig. 1A), and this transient state persists over hundreds of picoseconds. These are key requirements to fully exploit NiO and more generally CT insulators in recently proposed optoelectronic devices (*1*, *13*). Time-dependent density functional theory (TDDFT) calculations support our observation of a linear control of the CT gap reduction by photodoping and reveal that the main contribution is due to a renormalization of the on-site correlation in terms of an effective Hubbard *U*.

## RESULTS

### Long-lived dynamics at the CT gap

**Fig. 2.**
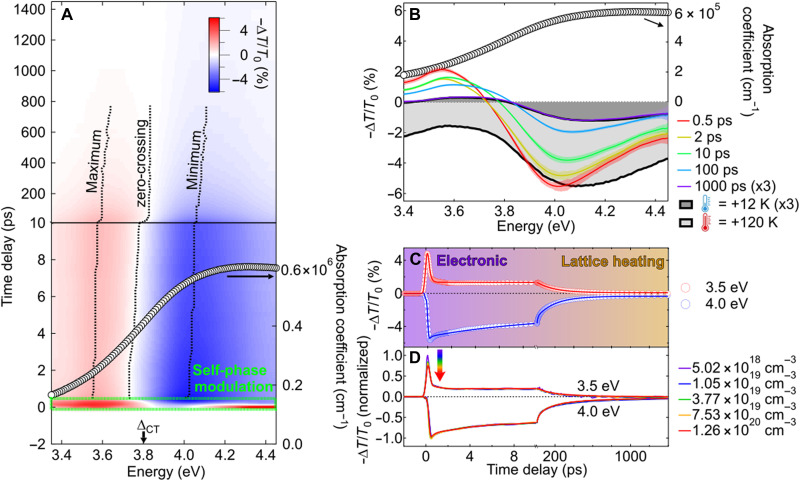
Electronic response of NiO persisting for hundreds of picoseconds, followed by the emergence of pure lattice heating. (**A**) Color-coded map of the transient transmission ( $-\;\Delta T\;/\;T_{0}$ ) of the NiO (001) thin film upon photoexcitation at 4.66 eV at room temperature. The excitation density is 1.26 × 10^20^ cm^−3^ (0.009 eh/uc). The NiO absorption coefficient is shown with black circles for reference (right axis). The green delimited region with dashed lines shows the signal affected by self-phase modulation. Black dashed lines represent the time evolution of the minimum, the zero, and the maximum of the transient signal. The energy of the CT gap ( $\Delta_{\text{CT}}$ ) is shown for reference (bottom axis). (**B**) Spectral traces and (**C**) kinetics of the NiO (001) thin film upon photoexcitation at 4.66 eV. The excitation density is 1.26 × 10^20^ cm^−3^ (0.009 eh/uc). Colored-shaded areas in (B) represent the SD between individual measurements. Gray shaded curves with black lines represent simulations of spectral traces caused by lattice heating by 12 K (dark gray) and 120 K (light gray) above room temperature (300 K), respectively. These simulations are on the basis of temperature-dependent spectroscopic ellipsometry [data from ref. (*53*) and section S1 and fig. S2B]. In (C), the transient transmission at selected probe energies (circles) are overlapped with fitted kinetics from a target analysis detailed in the main text (lines). Temporal regions where the electron response and lattice heating dominate the transient spectral response are displayed with colored areas. (**D**) Evolution of the normalized kinetics at probe photon energies of 3.5 eV (positive signal) and 4.0 eV (negative signal) at different excitation densities. Normalization is performed on the integrated signal amplitude between 1 and 2 ps.

We first look into the temporal evolution of the transient optical signal of NiO thin film with the pump-probe setup sketched in Fig. 1B (thin film deposition procedure and description of the pump-probe setup in Materials and Methods and thin film characterizations in section S1). Figure 2A shows the evolution of the transient transmission at room temperature upon photoexcitation at 4.66 eV [~50-fs pulses full width at half maximum (FWHM)] with a fluence of 260 $\mathrm{\mu J}\;{\text{cm}}^{–2}$ (excitation density of 1.26 × 10^20^ cm^−3^ corresponding to 0.009 electron-hole per unit cell or eh/uc). The signal starts after the self-phase modulation (*14*) at ~100 fs and has a wavelet line shape with distinct regions of positive (3.35 to 3.75 eV) and negative (3.75 to 4.45 eV) signals, corresponding to energies below and above the CT gap, respectively. These results are compatible with a red shift of the optical spectrum in the excited state (*8*, *15*). Notably, the wavelet persists over the nanosecond timescale, in stark contrast with the picosecond-lived wavelet signals observed in cuprates, which exhibit similar line shapes previously assigned to lattice heating (*16*–*18*). Furthermore, there is a progressive blue shift of the extrema of the transient signal and the zero-crossing point (black dashed lines in Fig. 2A), suggesting that the observed signal not only originates from population effects but also reflects time-dependent changes in the electronic structure.

Figure 2B shows selected spectral traces at time delays between 500 fs and 1 ns (colored curves). An excellent agreement is found between the spectral trace at 1 ns (purple curve) and the simulated transient transmission of NiO heated up from room temperature (300 K) by an increment of 12 K (dark gray shaded curve with black line), in agreement with the calculated temperature jump of 10 K induced upon transfer of the pump excess energy to the lattice and after heat transfer to the MgO substrate (see section S2 for more details). Spectral traces at 0.5-, 2-, and 10-ps time delays cannot be uniquely reproduced by the simulated transient signal of a hot lattice because the simulation shows purely negative transient signals for larger temperature jumps with mismatched extrema along the spectral axis and unrealistic temperature differences for the absorbed pump fluences (light gray shaded curve with black line in Fig. 2B). Hence, nonthermal effects dominate at picosecond time delays whereas lattice heating is dominant at nanosecond time delays (represented by colored areas in the kinetics of Fig. 2C).

A target analysis of the kinetics shows that two parallel exponential decay components are required to describe the kinetics between 0.5 and 200 ps, assigned to the decay of the electronic response coupled to lattice heating, followed by the rise of a saturating exponential component [ $\propto \left(1-e^{-t/\tau }\right)$ ], assigned to pure lattice heating (fittings in Fig. 2C; details in section S3). The time constants of the electronic decay are found to be independent of the pump excitation density with time constants of ~10 and ~200 ps. The short time constant is compatible with the emergence of nonthermal lattice deformations reported in (*19*) but has not been observed in previous ultrafast XAS measurements (*10*). The long time constant is consistent with a previously reported decay time assigned to polarons using transient absorption/reflectivity in the extreme UV (*20*). The analysis confirms that lattice heating dominates the transient signal at time delays ≳200 ps, whereas the transient signal at <10 ps is not exclusively thermal. The long rise time of a purely thermal phase is remarkable and in line with other works on the same material (*19*, *21*). It indicates that a long-lived nonthermal metastable state is stabilized in NiO for hundreds of picoseconds, possibly upon the formation of polarons as recently proposed (*10*, *20*, *22*). We exclude that this long-lived state originates from the stabilization of photoexcited carriers at point defects, based on complementary transient reflectivity measurements showing the decay of carriers populating shallow traps in ~3 ps and the absence of long-lived trapped carriers at deep trap states (section S7 and fig. S31) and because of the low point defect concentration in the investigated materials (section S1.6). The metastable state could be used for optoelectronic applications.

### Linearity of the transient spectral response

**Fig. 3.**
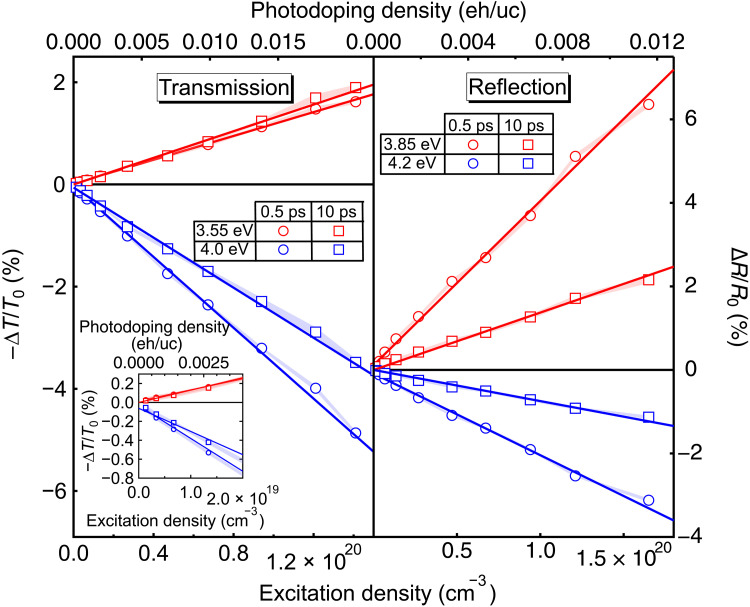
Fully linear optical response achieved over picosecond timescales. Evolution of the transient amplitude at 0.5 ps (circles) and 10 ps (squares) with the excitation density (photodoping density, top axis) at probe photon energies 3.55 eV (blue) and 4.0 eV (red). Shaded areas are SDs between individual measurements. Linear fits (continuous curves) are shown. Inset is the same plot in the region of low excitation densities. The transient transmission is plotted as a negative relative change ( $-\;\Delta T\;/\;T_{0}$ ) with the equilibrium transmission $T_{0}$ such that, in the absence of large changes in the real part of the refractive index, negative signals represent a decreased absorption and positive signals represent an increased absorption. The transient reflectivity is plotted as a relative change ( $\Delta R\;/\;R_{0}$ ) with the equilibrium reflectivity $R_{0}$.

We now look at the linearity of photoinduced changes in NiO thin films. The dependence of the photoinduced changes in transmission ( $-\;\Delta T\;/\;T_{0}$ ) and reflection ( $\Delta R\;/\;R_{0}$ ) are displayed in Fig. 3 after pumping the sample with 4.66-eV pulses. The transient spectral response is found to be fully linear with the excitation density over two orders of magnitude (between 1.0 × 10^18^ and 1.5 × 10^20^ cm^−3^). That this behavior is observed both in transmission and reflection indicates that it is homogeneous across the sample (more details in section S4 and fig. S10).

The extended linearity in the transient optical response of NiO is uncommon upon photodoping as a function of the excitation density of solids where many-body effects often lead to nonlinear variations. For instance, previous transient photodoping studies in cuprates and in nickelates show nonlinear evolutions with an excitation density below ~0.005 eh/uc (*17*, *23*) whereas, here, NiO displays a linear response up to ~0.02 eh/uc (details of the photodoping concentration calculation in section S5). The relative transmission changes, of the order of a few percent, are modest in the investigated range of photodoping compared with photonic crystals (*24*) or metamaterials (*25*). Extension of this dynamic range to higher excitation densities is limited by the regime of Coulomb screening, which becomes nonlinear when screening lengths become comparable to interparticle distances. However, carrier localization through polaron formation may extend the linear regime due to reduced carrier interactions (*22*) and thus achieve larger linear optical changes. The linearity is achieved on subpicosecond timescales and persists up to 100 ps, which is notably longer than with photonic or metamaterials (*24*, *25*). The fully linear dynamic range controlled by the light intensity and the time delay between the pump and the probe offers avenues to the design of next-generation optical switches.

### Constant transient kinetic response

Long-lived metastable states can be used for applications under the condition that they recover the equilibrium state with unchanged time constants such that the repetition rate of optoelectronic devices remains independent of the working conditions. Figure 2D displays the evolution of normalized kinetics with the excitation density measured in transmission. The kinetic traces at 3.5-eV (positive traces) and 4.0-eV (negative traces) probe photon energies remain unchanged for excitation densities between 5.02 × 10^18^ and 1.26 × 10^20^ cm^−3^ (between 0.0004 and 0.009 eh/uc). Similar results are obtained in reflection up to 1.84 × 10^20^ cm^−3^ (0.013 eh/uc) (section S6 and fig. S20). The initial excitation has almost fully recovered after 1.4 ns, showing that the material can be reversibly excited up to a repetition rate of ~700 MHz. The robustness of the kinetics with the excitation density is important for the development of passive ultrafast optoelectronics, such as optical switches (*26*) or triggered optical modulators (*27*), because the wide dynamic range of operation should maintain the maximum repetition rate achievable, limited by the recovery time between two consecutive optical stimuli.

Consistent results were obtained in reflection on NiO single crystals with different surface orientations and pump photon energies above the CT gap at fixed excitation densities, revealing that the observed effects do not depend on the sample morphology, surface orientation, or carrier excess energy for crystalline NiO (see section S7 and figs. S22 to S30).

### Electronic response to photodoping: Renormalization of Hubbard *U* and dynamical screening

**Fig. 4.**
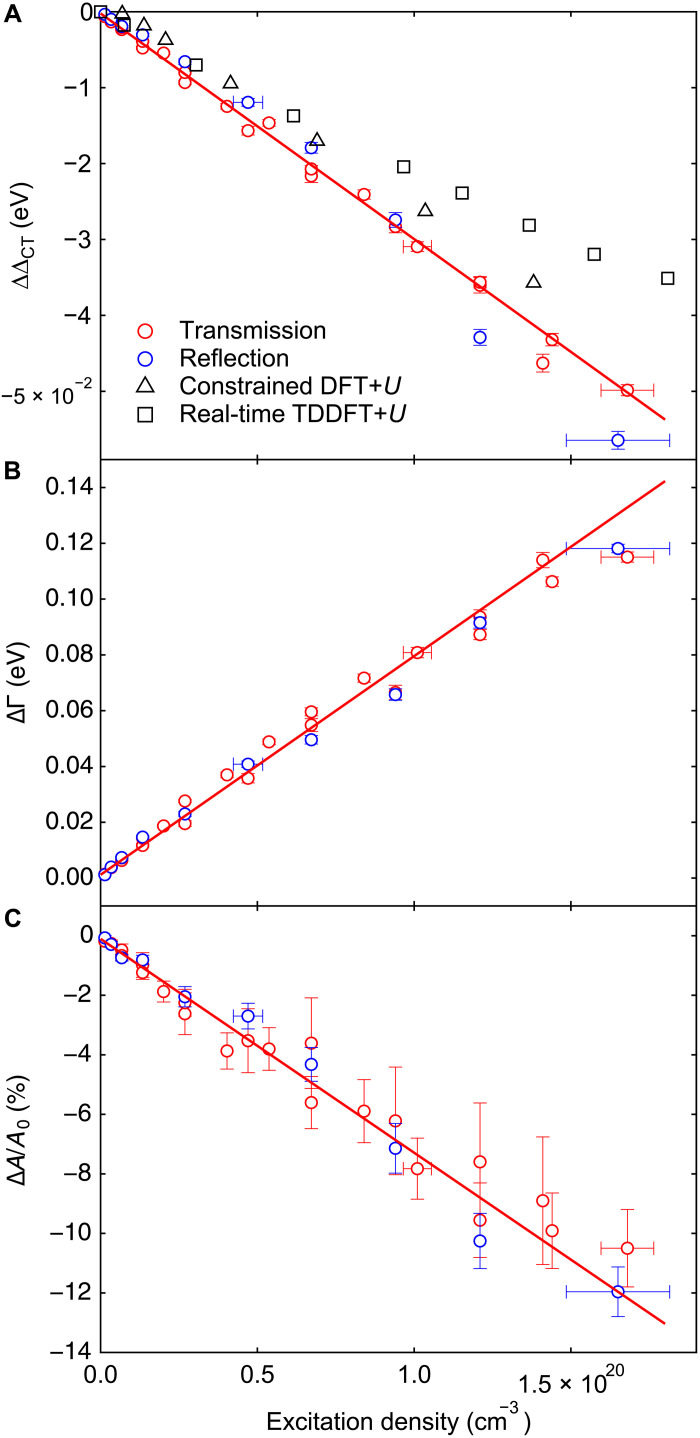
Controlling the CT gap of NiO with photodoping. Fitted (**A**) bandgap energy reduction ( ${\Delta \Delta }_{\text{CT}}$ ), (**B**) broadening change ( ΔΓ ), and (**C**) change in relative oscillator strength ( $\Delta A\;/\;A_{0}$ ) of the Tauc-Lorentz oscillator at the CT gap (2-ps time delay and 4.66-eV excitation). Results are displayed for measurements in transmission (red circles) and in reflection (blue circles). Continuous red curves are linear fits to the parameter values for the measurements in transmission. The vertical error bars indicate 95% confidence intervals calculated from the SE of the fitting parameters. Deviations of some data points from the linear fit outside the confidence intervals are attributed to slight inhomogeneities in stoichiometry and sample thickness, which are not included in the estimate of the confidence intervals. The main uncertainty is in the calculation of the excitation density, which is shown with horizontal error bars for a few points. Calculated bandgap reduction with constrained DFT+*U* (black triangles) and real-time TDDFT+*U* (black squares) are displayed in (A).

We now focus on the microscopic interpretation of the NiO spectral response to connect the spectral changes to modifications of the electronic structure. Between 0.5 and 100 ps, spectral traces in transmission of NiO thin films (Fig. 2B) have different relative amplitudes in the positive and negative parts indicating that they cannot be simply reproduced by a spectral red shift or a broadening of the equilibrium spectrum alone (see simulations in section S8). Instead, this points to the simultaneous contribution of both a spectral shift and broadening. We developed an analytical model to track the evolution of both contributions to the transient line shape, similar to the methodology previously used for semiconductors photoexcited near their optical bandgap (*28*, *29*). Spectroscopic ellipsometry measurements are used to track the evolution of the relative permittivity of the NiO thin film (see section S1.3.2). The real and imaginary components of the relative permittivity are simultaneously fitted in the CT gap region using a phenomenological model of classical oscillators with two Tauc-Lorentz resonances and one Lorentz resonance, which achieves good agreement within the energy range between 1.0 and 6.0 eV (see section S1.3.2 and fig. S3B). The fitted oscillator parameters (see section S9 and table S5) are used to model the transmission of the unpumped (equilibrium) NiO thin film and remain fixed when fitting the transient signal. The same oscillator model is applied to fit the pumped (excited-state) transmission using a set of only a few free parameters from the two oscillators contributing at the CT gap. Details of the fitting procedure can be found in section S9.1. The fits achieved good agreement with the transient transmission and reflection at 2-ps time delay (see section S9 and figs. S33 and S35). Figure 4 shows the evolution at 2 ps with the excitation density of the CT gap energy change ( ${\Delta \Delta }_{\text{CT}}$; Fig. 4A), the broadening change ( ΔΓ; Fig. 4B), and the change in relative oscillator strength ( $\mathrm{\Delta A}\;/\;A_{0}$; Fig. 4C) given the parameters of the Tauc-Lorentz oscillator contributing the most to the absorption at the CT gap for the measurements in transmission (red circles) and in reflection (blue circles), yielding consistent results. The CT gap energy is found to decrease linearly with the excitation density, whereas the broadening Γ displays an opposite trend (see linear fittings in Fig. 4, A and B). The CT gap reduction is in line with theoretical predictions that photodoped carriers contribute to the screening of the electronic correlations, which renormalizes the correlation gap (*1*, *8*) or the CT gap (*15*, *30*). The broadening increase ΔΓ is a many-body dynamical screening effect of the density of states at the bandgap coupling with low energy plasmon modes (*15*, *30*) (illustrated in Fig. 1A). We find that the photoinduced renormalization of $\Delta_{\text{CT}}$ and the spectral broadening are quantitatively consistent with results recently reported by femtosecond XAS in NiO thin films (*10*) (detail in section S10). The relative oscillator strength decreases linearly with the excitation density (Fig. 4C), which is assigned to phase-space filling by photoexcited carriers at the band edges commonly observed in semiconductors (*31*).

Previous theory works on the photodoping of CT insulators have suggested that the CT gap ( $\Delta_{\text{CT}}$ ) renormalizes with the excitation density due to either the renormalization of the electronic correlation energy through dynamical screening (using TDDFT+*U*) (*1*, *8*) or the combined effects of a Hartree shift and dynamical screening [using nonequilibrium dynamical mean-field theory (DMFT)] (*15*, *30*). In both cases, an explicit treatment of dynamic correlations is essential to model the effect of light excitation on the electronic structure of materials. In the TDDFT+*U* framework, the $\Delta_{\text{CT}}$ reduction is due to the renormalization of the Hubbard *U*, which implies that the reduction of $\Delta_{\text{CT}}$ between the oxygen p and UHB is given by −ΔU/2 (Fig. 1A) (*8*). The results in Fig. 4A show a change in electronic correlations ΔU∼100meV at an excitation density of $∼1.5\times 10^{20}\;{\text{cm}}^{-3}$ (~0.011 eh/uc), which is in contrast with previous measurements with nonresonant strong field excitation despite a small amount of photodoping across the CT gap (*32*), for different laser conditions. Photodoping in cuprates achieves up to ΔU∼140meV for an excitation density of a few percent per unit cell (*9*), a few times higher than the renormalization in NiO at similar excitation densities. We investigate further the screening of *U* with ab initio calculations in the next section.

### Ab initio calculations

To understand the origin and the scaling of the bandgap reduction with the excitation density, we performed ab initio constrained density functional theory (cDFT) and real-time TDDFT calculations including fully self-consistent and parameter-free (*8*, *33*) treatments of electronic correlations with an effective Hubbard *U* term using the ACBN0 functional (*34*) that was found to reproduce the linear and nonlinear responses of NiO. The calculated reduction of the CT gap upon photodoping with both theoretical methods is displayed in Fig. 4A, which corresponds to half the reduction of the Hubbard *U* in excellent approximation (section S11 and fig. S39B), showing that the observed changes in the electronic structure are mostly due to the renormalization of electronic correlations. Both methods display a nearly linear reduction of $\Delta_{\text{CT}}$ with the excitation density, consistent with the experimental results. The cDFT+*U* calculations illustrate the effect of thermalized carrier populations over the bandgap. It shows a correct prediction of the magnitude of the CT gap renormalization but does not exhibit a saturation of this effect at high excitation densities, which is expected for many-body effects and previously predicted by DMFT (*10*). TDDFT+*U* instead shows that a dynamic treatment of the excitation (and of the Hubbard *U* in particular) is required to model the saturation of electronic changes in out-of-equilibrium NiO at high excitation densities, which implies that the electronic states hereby observed are transient metastable states with no achievable equivalent under continuous light illumination. The gap reduction predicted by real-time TDDFT+*U*, estimated from the change in *U*, underestimates the gap reduction by ~40% in this model. The missing part of the calculated bandgap reduction is likely to come from the dynamical screening of the energy states, as well as nonlocal correlations, which are not evaluated accurately with TDDFT with the approximated ACBN0 functional (*35*), in contrast to the spectral dependence of the screened interaction implemented in DMFT (*36*). An additional contribution may come from the formation of polarons, which has been used to interpret the ultrafast energy renormalization of the optical gap in cuprates (*18*) and the linearity of the x-ray absorption changes with the excitation density in NiO using ultrafast x-ray absorption spectroscopy (*20*, *22*). These results show that, for our laser excitation conditions, most of the NiO bandgap reduction comes from the reduction of the local Hubbard *U*, with a key contribution from nonthermal distributions of carriers and that this reduction is linearly controlled by the excitation density achieved with the optical pump pulse. Details of the numerical simulations can be found in Materials and Methods and in section S11.

## DISCUSSION

Previous works reported bandgap renormalization in photodoped uncorrelated insulators recovering on subpicosecond timescales (*31*, *37*, *38*), whereas others claim that it persists as long as photoexcited carriers are present (*39*–*41*). An important aspect of the bandgap reduction is that lattice heating also contributes, which originates from a combination of lattice expansion and phonon-induced atomic vibrations in polar crystals (*42*). Strongly correlated materials in particular exhibit a very efficient energy transfer between charge carriers and the lattice, the latter usually heating up on subpicosecond timescales (*43*). Hence, the longest time necessary to recover the photoinduced bandgap renormalization is due to heat diffusion and heat dissipation away from the excitation volume. The target analysis performed in this work (section S3) shows that lattice heating is the largest contribution to the transient signal at ~100 ps and that a nonthermal reduction of the CT gap is dominant in NiO on <10-ps timescales but persists over a timescale of several hundreds of picoseconds. In other correlated materials, electronic correlations lead to a very efficient electron-hole recombination, usually on subpicosecond timescales, which leads to materials with purely thermal responses in a few picoseconds. It implies that correlated carriers in NiO are likely stabilized to increase their lifetime, for instance, by forming polarons (*20*, *22*), leading to reduced interactions between carriers or due to the coupling between the spin and the lattice degrees of freedom. Structural deformations have been reported upon excitation of NiO with nonthermal atomic displacements emerging in ~0.3 ps and persisting for at least 25 ps, assigned to photoinduced lattice distortions associated with changes in the antiferromagnetic exchange striction coupling (*19*). The quick emergence and persistence of these nonthermal structural changes are compatible with the current results, leading us to conclude that the NiO nonthermal metastable state is due to structural deformations. In this respect, optical studies below the NiO CT gap should reveal the existence of quasiparticles, similar to the previous works on cuprates (*16*–*18*, *44*).

This work demonstrates simultaneous control over the electronic structure and the dynamics of NiO. Together with the long-lived metastable electronic state with renormalized electron correlations, the results are a promising avenue to ultrafast optoelectronics in correlated CT insulators, which requires the combined control shown here. Future studies should focus on the effect of the initial photodoping and the lattice temperature on subpicosecond timescales, where the effect of the NiO antiferromagnetic ordering should play a role in the dynamics of carriers (*45*, *46*) and the magnitude of the renormalization of electronic correlations (*47*).

## MATERIALS AND METHODS

### Deposition of the NiO thin film

The NiO (001) thin film was grown by pulsed laser deposition. Double-sided MgO substrates from MTI Corporation were solvent cleaned and then affixed to a sample plate with silver paint before being transferred into the vacuum chamber. The substrates were held at a temperature of 400°C, and the NiO film was deposited from a stoichiometric target in a background pressure of 0.14-mtorr O_2_ at a gas flow of 2.2 SCCM (standard cubic centimeter per minute). A KrF excimer laser, at a repetition rate of 10 Hz and a fluence of 1 J cm^−2^, ablated the material from the NiO target onto the substrate plate with a separation between target and substrate of 46 mm. To ensure proper oxygenation of the NiO film, the sample was cooled to room temperature in a 300-torr O_2_ atmosphere.

### Transient transmission/reflection setup

The ultrafast broadband UV experiments have been performed with a setup providing narrowband UV pump and broadband UV probe pulses between 3.35 and 4.8 eV (illustrated in Fig. 1B). The setup has been extensively described in refs. (*11*, *12*). We hereby briefly summarize the main parameters of the setup. First, a 20-kHz Ti:sapphire laser and cryocooled regenerative amplifier (KMLabs, Halcyon oscillator and Wyvern500 amplifier) provide 50-fs FWHM pulses at 1.55 eV with typically ~0.7 mJ per pulse (12-W average power). Around 7 W is used to pump a noncollinear optical parametric amplifier (NOPA; TOPAS-White, Light Conversion), which provides <100-fs pulses with a very broad spectral coverage typically between 1.65 and 2.5 eV with ~7 μJ per pulse in this broadband configuration. Around 60% of the NOPA output power is used to generate the narrowband pump pulses. In this optical line, the visible pulse goes through a chopper operating at 10 kHz, synchronized to the laser system and phase-locked manually via the detection of the transmitted intensity with a photodiode. The pump pulse goes through an interference filter to select the fundamental of the pump photon energy and then through a phase-matched β-barium borate (BBO) crystal to provide the UV pump pulse. The typical BBO thickness is less than 1 mm to conserve the temporal width of the pump pulse. The typical bandwidth of the UV pump pulse is 20 meV with a pulse energy of the order of 100 nJ. The pump pulse power density is recorded on a shot-to-shot basis by a calibrated photodiode for each pump photon energy, which allows for the normalization of the transient data based on the pump pulse energy. A half waveplate is used to set the relative polarization between the pump and the probe pulse at the magic angle (54.74°) to get rid of photoselection effects.

The remaining NOPA power is used to generate broadband UV probe pulses with ~1.7-eV bandwidth through an achromatic doubling scheme (sketched in Fig. 1B), which has been developed in the Riedle group (*48*). It comprises two fused silica prisms that spatially disperse and recollimate the visible beam coming from the NOPA. The resulting spatially chirped beam is focused with a 90° off-axis parabolic mirror on a 200-μm-thick BBO crystal. The frequency doubling of such broadband visible pulse is complex because of a spectrally dependent phase-matching condition. Hence, the spatial chirp of the visible beam and the different incident angles achieved by the parabola onto the BBO need to be phase-matched at every probe photon energy simultaneously. The prisms also induce a temporal chirp required to avoid frequency mixing at the BBO. The frequency-doubled beam is subsequently recollimated with another 90° off-axis parabola, recombined and recompressed with two additional CaF_2_ prisms.

The pump and probe pulses are focused onto the sample (the pump is at normal incidence and the probe at ~7° in the refraction convention) where they are spatially and temporally overlapped. The beam waists are typically ~80 μm for the pump and ~20 μm for the probe, which result in a homogeneous probing of the excited sample volume. The reflected beam is steered, collimated, and focused to couple into a multimode optical fiber (100 μm), which is connected to the entrance slit of a 0.25-m imaging spectrograph (Chromex 250is). The beam is dispersed by a holographic grating of 150 grooves/mm and imaged onto a multichannel detector consisting of a 512-pixel CMOS (complementary metal-oxide semiconductor) linear sensor (Hamamatsu S11105, 12.5 μm–by–250 μm pixel size) with up to 50-MHz readout. The maximum readout rate per spectrum (almost 100 kHz) allows for easy shot-to-shot detection. The setup typically offers a time resolution of ~150 fs, but it can be improved to ~80 fs with a set of chirp mirrors or a prism compressor in the pump line. Additional details about the computation of signals are provided in section S12.

### TDDFT simulations

All the calculations presented here were performed for bulk NiO, which is a type II antiferromagnetic material below its Néel temperature [ $T_{N}=523\;K$ (*49*)]. We neglected the small rhombohedral distortions and considered NiO in its cubic rock-salt structure, which does not affect the result of calculated optical spectra. Calculations were performed neglecting spin-orbit coupling using fully norm-conserving pseudopotentials. We used a lattice parameter of 4.1704 Å, a real-space spacing of Δr=0.31 bohr, and a 16×16×8 k-point grid to sample the Brillouin zone. The driving field is taken along the [100] crystallographic direction in all the calculations. We consider a laser pulse of 55-fs duration (FWHM), with a sin-square envelope for the vector potential. The experimental carrier wavelength λ=266.06nm was used, corresponding to the experimental carrier photon energy of 4.66 eV. In all calculations, we set the carrier envelope phase to zero. The time-dependent wave functions, number of excited electrons, and $U_{\text{eff}}$ are computed by propagating generalized Kohn-Sham equations within real-time TDDFT+*U*, as provided by the Octopus code (*50*). We used the LDA (local density approximation) functional (*51*) for describing the semilocal DFT part, and we computed the effective $U_{\text{eff}}=U-J$ for the O 2p ( $U_{\text{eff}}^{2p}$ ) and Ni 3d orbitals ( $U_{\text{eff}}^{3d}$ ), using localized atomic orbitals from the corresponding pseudopotentials (*34*). All calculations are propagated for 15 fs after the end of the pulse to avoid spurious numerical effects. We also performed constrained DFT+*U* simulations. For this, we used the constrained DFT method proposed in ref. (*52*), in which we constrained the number of excited electrons from the experimental values and obtained the ground state and the self-consistent effective Hubbard *U* for each values of the number of excited electrons.

## Supplementary Materials

### This PDF file includes:

Sections S1 to S12 Figs. S1 to S40 Tables S1 to S11 References
