# Supplementary material for: Dynamic control of electron correlations in photodoped charge-transfer insulators
Source: Sci Adv. 2025 Sep 5;11(36):eadx5676. doi: 10.1126/sciadv.adx5676 (PMC12412645; doi:10.1126/sciadv.adx5676)
Supplement: Supplementary file 1 — Sections S1 to S12 Figs. S1 to S40 Tables S1 to S11 References [file sciadv.adx5676_sm.pdf]

Science Advances
AAAS

Supplementary Materials for

Dynamic control of electron correlations in photodoped charge-transfer insulators

Thomas C. Rossi *et al.*

Corresponding author: Thomas C. Rossi, thomas.rossi@helmholtz-berlin.de;
Majed Chergui, majed.chergui@epfl.ch

Sci. Adv. **11**, eadx5676 (2025)
DOI: 10.1126/sciadv.adx5676

This PDF file includes:

Sections S1 to S12
Figs. S1 to S40
Tables S1 to S11
References

1 Sample characterization

1.1 Sample growth

The NiO single crystal with (001) surface orientation was purchased from Mateck GmbH. It is grown with a furnace method, is black in color, fully opaque and electrically insulating. The NiO single crystal with (101) surface orientation was grown by the Chemical Vapor Transport (CVT) technique. In this process, NiO powder and $800\,$mbar of electronic grade HCl were encapsulated in a quartz ampoule. The sealed ampoule was then positioned in a two temperature zones horizontal furnace. Optimal temperatures for the source and growth sides of the ampoule were determined to be $960°$C and $800°$C, respectively. After two weeks, dark green crystals, measuring a few millimeters, were obtained. A subset of these crystals was crushed into powder and subjected to X-Ray Diffraction (XRD) analysis, confirming their composition as NiO without the presence of any detectable impurities.

1.2 Stoichiometry

The relative concentration between nickel and oxygen was characterized using energy dispersive X-ray analysis (EDX) from a JEOL JXA-8530F HyperProbe instrument. The technique can provide a quantitative ratio between the nickel and oxygen stoichiometry using standard reference materials (*54*). Figure S1a shows a profile of oxygen to nickel stoichiometric ratio over a distance of $10\,\mu$m. A weighted average with the uncertainty of the measurement indicates that the relative oxygen to nickel stoichiometry is $99.4(2)\,\%$, which shows that NiO single crystals in this work are nearly stoichiometric with a small oxygen deficiency. A microprobe picture of the sample fragment is shown in Figure S1b.

1.3 Optical properties by spectroscopic ellipsometry

The spectroscopic ellipsometry measurements were performed with a SE-2000 instrument from Semilab equipped with microfocus optics and a CCD array. For each sample, spectra were recorded at 9 independent points arranged in a square-shaped grid (with a spacing that depends on the sample dimension). The spectra were compared to estimate the site-to-site fluctuations of the sample optical properties. Since the measurements were fully reproducible with small standard deviations, we only report the average spectra. Both thin film and single crystal samples were measured with the same instrument. For NiO single crystal, the conversion between the elliptical light parameters (Ψ, Δ) and

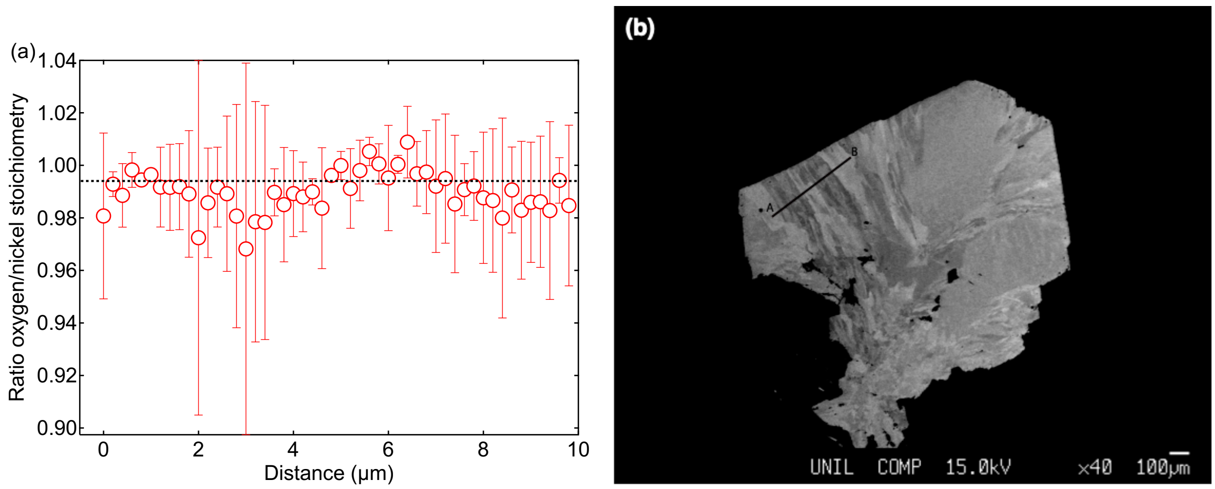

Fig. S1: **Spatial profile of NiO stoichiometry.** (a) Profile of stoichiometry ratio between oxygen and nickel in NiO (101) single crystal grown by chemical vapor transport. The vertical error bars represent propagated uncertainties from the estimate of the intensity profiles in the EDX patterns. An horizontal dashed line represents the weighted average of the stoichiometry by the uncertainties. (b) NiO (101) single crystal image from the field emission electron probe. The black line represents the line along which the stoichiometry profile is measured (the line is made longer than the actual dimension of the probed profile for clarity).

the NiO optical constants (ϵ_1, ϵ_2 for instance) were performed with our own script. The modeling of the optical parameters was performed with the SpectraRay/4 software for NiO thin film and with the Reffit software for NiO single crystal (*55*).

1.3.1 NiO single crystal

In the spectroscopic ellipsometry measurement of NiO single crystals, the complex refractive index $\tilde{n}$ is computed from the ellipsometry parameters Ψ and Δ using the equation for the light reflection on a semi-infinite medium,

$$\tilde{n} = \sin\theta_0 \sqrt{1 + \left(\frac{1-\rho}{1+\rho}\right)^2 \tan^2\theta_0} \tag{S1}$$

with $\rho = e^{i\Delta}\tan\Psi$ and θ_0 the angle of incidence. Figure S2a shows the absorption coefficient α computed from $\alpha = 4\pi\Im(\tilde{n})/\lambda_0$ with $\Im$ the imaginary part operator and λ_0 the vacuum wavelength of the light. The absorption profile is similar to previously reported measurements (*19, 56*) (although slightly red shifted) with an absorption edge rising between 3.5 and $4.0\,$eV followed by a decrease at higher photon energies. A substantial absorption is displayed below the rising edge, which has been

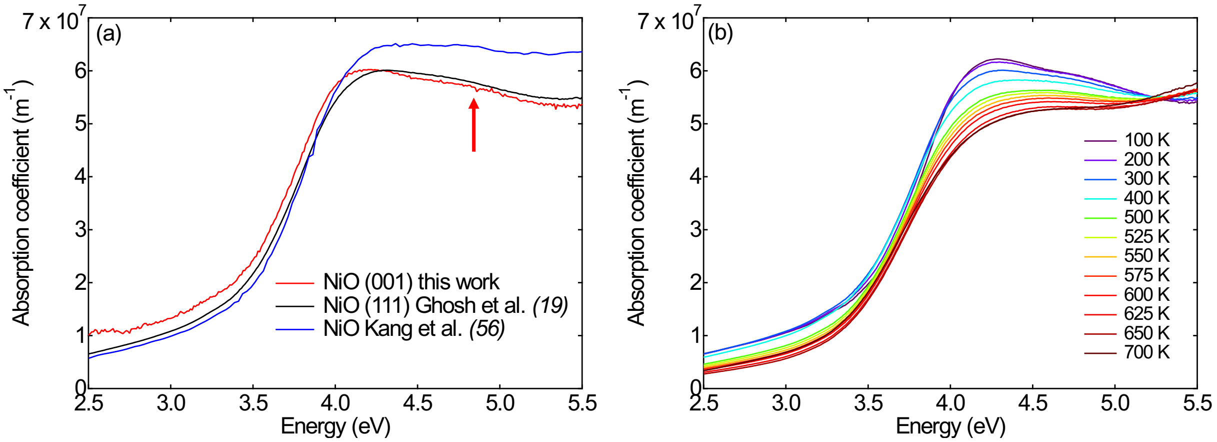

Fig. S2: **Absorption spectra.** (a) Absorption coefficient calculated from ellipsometry measurements on NiO (001) single crystal (red curve). Data from Kang *et al.* (blue curve, (*56*)) and Ghosh *et al.* (black curve, (*19*)) are reproduced for comparison. (b) Temperature dependence of NiO (001) single crystal absorption coefficient (obtained from ellipsometry measurements with original data in (*19*)).

pump energy (eV)	absorption coefficient (m^{-1})	penetration depth (nm)	reflectivity
3.54	2.58×10^7	38.8	0.22
3.97	5.60×10^7	17.9	0.18
4.28	6.01×10^7	16.6	0.11
4.51	6.06×10^7	16.5	0.09

Tab. S1: **Optical constants of NiO single crystal at pump photon energy.** Absorption coefficient, penetration depth, and reflectivity at the pump photon energies for the pump-probe measurements on NiO (001) single crystal. The reflectivity is at normal incidence.

previously reported (*58, 59*). It is mainly due to transitions from the valence band to a conduction band derived from $4s$ nickel orbitals (*57*), overlapping with sharp absorptions from crystal field multiplets observed in transmission measurements (*58*). A weak resonance can be seen at ~ 4.8 eV, which is also present in previous measurements (*19, 56, 60*) (red arrow in Figure S2a). This weak transition is between two bands derived from Ni 3d(t_{2g}) and Ni 3d(e_g) orbitals, which is dipole forbidden in a centrosymmetric coordination geometry (*60*). The NiO absorption coefficient, penetration depth and reflectivity (Figure S3a) at the pump photon energies of the measurements on NiO single crystals are displayed in Table S1.

The permittivity of the NiO single crystal is shown in Figure S3b. The real and imaginary parts are

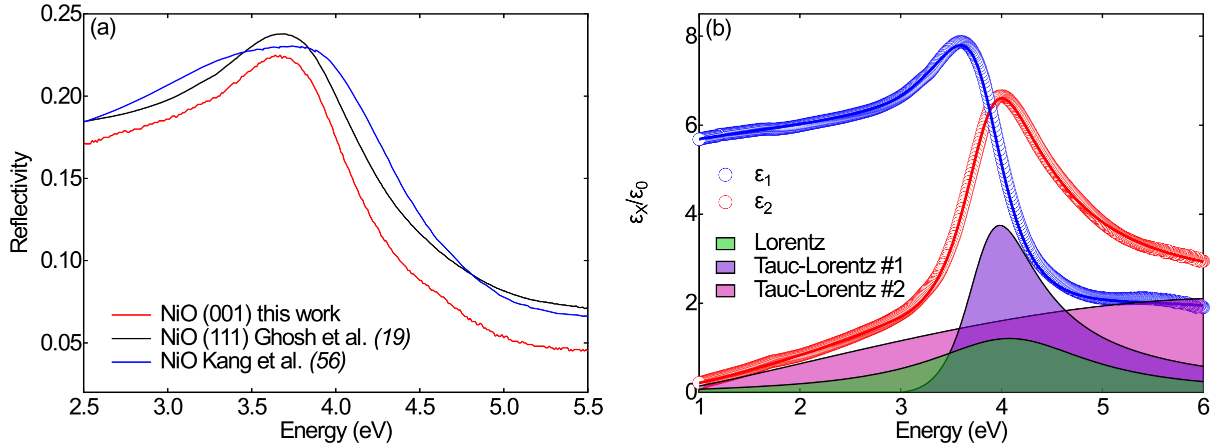

Fig. S3: **Reflectivity and permittivity.** (a) Comparison between the reflectivity of NiO (001) single crystal computed from our ellipsometry data with the reflectivity from other ellipsometry studies (*19,56*). (b) Real part (blue circles) and imaginary part (red circles) of the permittivity of NiO (001) single crystal. Fittings of the permittivity using a model involving two Tauc-Lorentz oscillators, one Lorentz oscillator and a UV pole are also shown (continuous curves). The decomposition into individual oscillators is shown for the Lorentz oscillator (shaded green), Tauc-Lorentz oscillator 1 (shaded purple), and Tauc-Lorentz oscillator 2 (shaded pink) curves.

fitted simultaneously with a model-independent Kramers-Kronig constrained variational algorithm (*55*). The model is based on a combination of two Tauc-Lorentz oscillators (*61*), one Lorentz oscillator and one UV pole. Tauc-Lorentz oscillators are popular choices to reproduce the NiO permittivity in the energy range 3.3-4.5 eV (*19, 62, 63*). The fitting results are displayed with continuous curves in Figure S3b and the fitted parameters are given in Table S2.

This work makes an extensive use of previously reported temperature-dependent spectroscopic ellipsometry measurements on NiO single crystal (*19*). The temperature dependence of the absorption coefficient is shown in Figure S2b. Upon lattice temperature increase, the absorption coefficient in the above edge region decreases and broadens at the absorption edge. The analysis of the pseudodielectric function near the rising optical absorption edge indicates a reduction of the charge-transfer gap from 3.92 to 3.73 eV between 100 and 700 K as well as a broadening from 240 to 325 meV (see Figure 7 in reference (*19*)). The evolution of both the charge-transfer gap and the broadening is linear with the lattice temperature above room temperature. The permittivity of NiO with the lattice temperature between 0.7 and 6.2 eV and between 200 and 525 K is fitted with a model involving two Tauc-Lorentz oscillators, two Lorentz oscillators and one UV pole (selected fits are displayed in Figure S4). The fitted

oscillator	plasma frequency (eV)	E_g (eV)	E_0 (eV)	Γ (eV)
Tauc-Lorentz 1	53.4	2.95	3.86	0.86
Lorentz	3.12		4.19	1.95
Tauc-Lorentz 2	27.4	0.51	8.38	12.65

Tab. S2: **Oscillator parameters of NiO (100) single crystal at equilibrium.** Parameters of the oscillators in the fitted permittivity of NiO (001) single crystal with two Tauc-Lorentz oscillators and one Lorentz oscillator. The fitted real part of the optical permittivity is $\epsilon_{1\infty} = 1.8$.

parameters are then linearly interpolated to generate the oscillator parameters at the room temperature of the pump-probe experiments in this work. The list of here-obtained oscillator parameters which are located in the spectral window of the probe is provided in Tables S3 and S4.

The temperature-dependent spectroscopic ellipsometry data is used to simulate transient optical spectra of hot NiO lattices. The permittivity between $100\,\mathrm{K}$ and $700\,\mathrm{K}$ is interpolated using a cubic spline function to provide a two-dimensional function of the permittivity with the energy and the lattice temperature. The transient signal for NiO at the time delay t and the probe photon energy E with the lattice temperature $\mathcal{T}$ in the pumped state and $300\,\mathrm{K}$ in the ground state is calculated following,

$$-\frac{\Delta T}{T_0}(\mathcal{T}, E, t) = -\frac{T(\mathcal{T}, E, t) - T_0(300\,\mathrm{K})}{T_0(300\,\mathrm{K})} \tag{S2}$$

for a measurement in transmission at room temperature ($300\,\mathrm{K}$) with T the transmitted probe intensity and,

$$\frac{\Delta R}{R_0}(\mathcal{T}, E, t) = \frac{R(\mathcal{T}, E, t) - R_0(300\,\mathrm{K})}{R_0(300\,\mathrm{K})} \tag{S3}$$

for a measurement in reflectivity at room temperature with R the reflected probe intensity. The Fresnel equations, the incidence angle of the probe ($\sim 7°$ in the refraction convention) and the material thickness (for the measurement on NiO thin films see SI §1.4) are used to evaluate the reflectivity coefficient at each lattice temperature.

1.3.2 NiO thin film

Raw data from spectroscopic ellipsometry measurement of NiO (001) thin film on top of MgO (001) substrate at various incidence angles are displayed in Figure S5. The optical parameters are obtained by fitting with a model consisting of a NiO dielectric slab on a MgO semi-infinite substrate. The fitting is between 0.9 and $4.8\,\mathrm{eV}$. The model includes two Tauc-Lorentz oscillators, one Lorentz oscillator

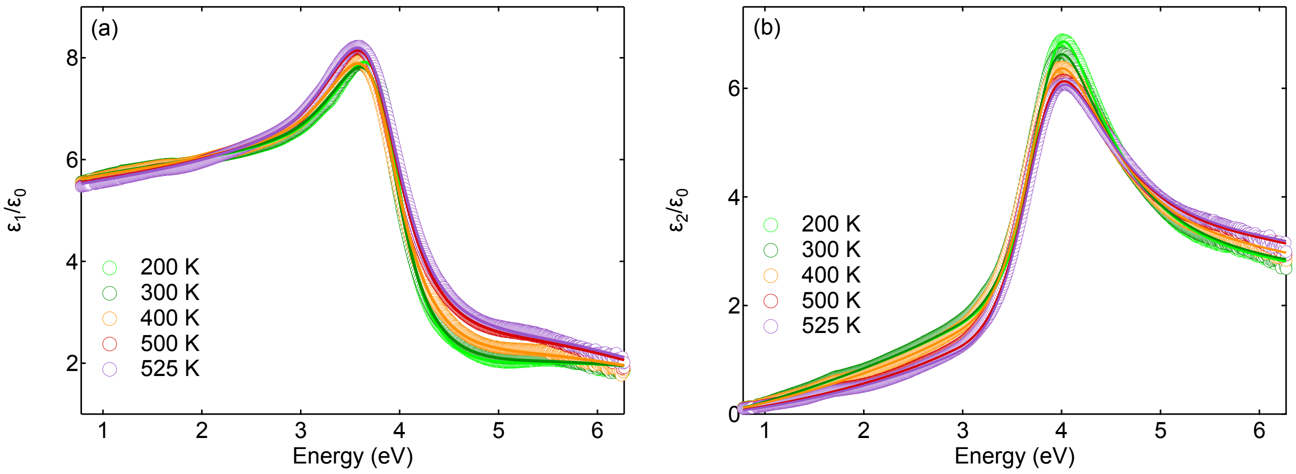

Fig. S4: **Temperature-dependent permittivity.** Relative permittivity of NiO single crystal as a function of lattice temperature from reference (*19*) (colored circles). (a) real part, and (b) imaginary part. Continuous curves are fittings using a model consisting of two Tauc-Lorentz, two Lorentz oscillators and one UV pole. The parameters of a few important oscillators for the analysis of the data are reported in Tables S3 and S4.

**lattice temperature (K)**	$\mathbf{A_1}$ (eV)	$\mathbf{A_2}$ (eV)	$\mathbf{A_3}$ (eV)	$\mathbf{E_{01}}$ (eV)	$\mathbf{E_{02}}$ (eV)	$\mathbf{E_{03}}$ (eV)
300	5.07×10^1	3.25	2.67×10^1	3.88	4.30	8.32
350	5.30×10^1	3.23	2.43×10^1	3.87	4.29	8.03
450	5.75×10^1	3.20	1.97×10^1	3.84	4.27	7.45
495	5.96×10^1	3.18	1.76×10^1	3.83	4.26	7.19
600	6.43×10^1	3.15	1.26×10^1	3.80	4.24	6.58

Tab. S3: **Temperature-dependent oscillator parameters (1).** Evolution of the amplitude ($\mathbf{A}$) and the resonance energy ($\mathbf{E_0}$) of the oscillators at selected lattice temperatures. Oscillators 1 and 3 are Tauc-Lorentz oscillators, oscillator 2 is a Lorentz oscillator.

**lattice temperature (K)**	$\mathbf{E_{g1}}$ (eV)	$\mathbf{E_{g3}}$ (eV)	$\mathbf{\Gamma_1}$ (eV)	$\mathbf{\Gamma_3}$ (eV)	$\epsilon_{\mathbf{1\infty}}$
300	2.93	4.76×10^{-1}	8.51×10^{-1}	1.89	1.85
350	2.93	4.77×10^{-1}	8.80×10^{-1}	1.91	1.94
450	2.93	4.80×10^{-1}	9.39×10^{-1}	1.97	2.12
495	2.94	4.81×10^{-1}	9.66×10^{-1}	1.99	2.21
600	2.94	4.83×10^{-1}	1.03	2.04	2.40

Tab. S4: **Temperature-dependent oscillator parameters (2).** Evolution of the band gap energy ($\mathbf{E_g}$) of the Tauc-Lorentz oscillators and optical permittivity ($\epsilon_{\mathbf{1\infty}}$) at selected lattice temperatures.

and a UV pole for the NiO dielectric slab and a Sellmeier dispersion law for the MgO substrate with parameters given in (*64*) (the fittings are displayed with continuous lines in Figure S5). Tauc-Lorentz oscillators have been successfully used in a number of previous studies to model the permittivity of NiO close to the CT gap (*19, 62, 63*). A roughness layer with a 50/50 composition is introduced at the interface between the thin film surface and air. The fitted parameters are given in Table S5. The parameters of the Tauc-Lorentz oscillator in the region of the charge-transfer gap are in good agreement with those obtained from previous spectroscopic ellipsometry measurements (Table S6). There is also an excellent agreement for the parameters of the Tauc-Lorentz oscillator 1 with the fitted parameters from the spectroscopic ellipsometry data by Ghosh and coworkers at room temperature (Table S3 with the measurement on our NiO thin film in Table S4). This Tauc-Lorentz oscillator is critical for the analysis of the pump-probe measurements since it contributes the most to the absorption at the CT gap. Hence, the analysis shows the robustness of the model, independent of the sample morphology. The thickness of the film is fitted at $17.5(3)$ nm, in excellent agreement with the thickness obtained from X-ray diffraction (see SI §1.4). The modeled optical properties of NiO give an absorption coefficient of 5.77×10^7 m^{-1} and a reflectivity of 0.076 at the pump photon energy of 4.66 eV. The penetration depth of the pump is thus 17.3 nm, which is comparable to the film thickness.

We additionally measured the UV-Vis spectrum of the NiO thin film in transmission. Measurements are performed on an Agilent Cary 8454 UV-Vis spectrophotometer equipped with a combination of deuterium and tungsten lamps to cover the spectral range between 190 and 1100 nm (red curve in Figure S6). The maximum of the optical absorption corresponding to the charge-tranfer gap is at ~ 4.1 eV, in good agreement with previous measurements on NiO thin films (*19*) and previous measurements on NiO single crystals (*58*) and in this work (SI Figure S3b).

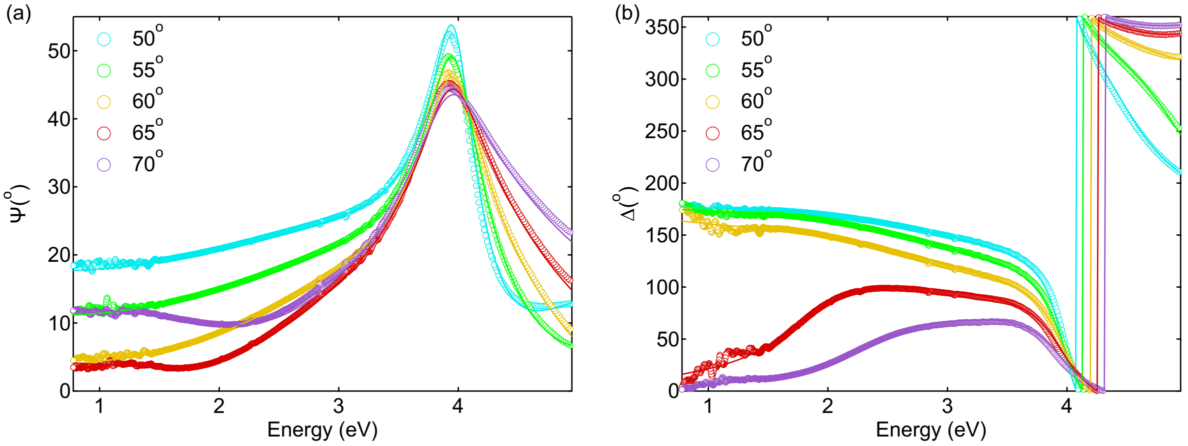

Fig. S5: **Ellipsometry constants.** Evolution of the ellipsometry parameters (a) Ψ and (b) Δ with the angle of incidence (colored circles). Continuous lines are fittings to the measurements using a model based on a Sellmeier dispersion equation for the optical properties of the MgO substrate, and two Tauc-Lorentz oscillators, one Lorentz oscillator and a UV pole for the optical properties of the NiO thin film.

oscillator	plasma frequency (eV)	E_g (eV)	E_0 (eV)	Γ (eV)
Tauc-Lorentz 1	49.2	2.93	3.87	0.84
Lorentz	3.30		4.26	1.96
Tauc-Lorentz 2	28.4	0.47	8.69	12.99

Tab. S5: **Oscillator parameters of NiO (001) thin film at equilibrium.** Parameters of the fitting of the dielectric constant of NiO (001) thin film with two Tauc-Lorentz oscillators and one Lorentz oscillator. Γ refers to the oscillator broadening, E_0 to the oscillator energy and E_g to the band gap. The real part of the optical dielectric constant is $\epsilon_{1\infty} = 1.75$.

	A (eV)	E_0 (eV)	E_g (eV)	Γ (eV)	$\epsilon_{1\infty}$
this work	49.2	3.87	2.93	0.84	1.75
(*62*)	111.89	3.96	3.30	0.82	2.50
(*63*)	39.778	4.048	2.679	1.354	2.251

Tab. S6: **Comparison of oscillator parameters of NiO (001) thin film with literature values.** Comparison between the parameters of the Tauc-Lorentz oscillator used to reproduce the optical permittivity of NiO thin films near the charge-transfer gap at room temperature in this work and in previous works (*62, 63*).

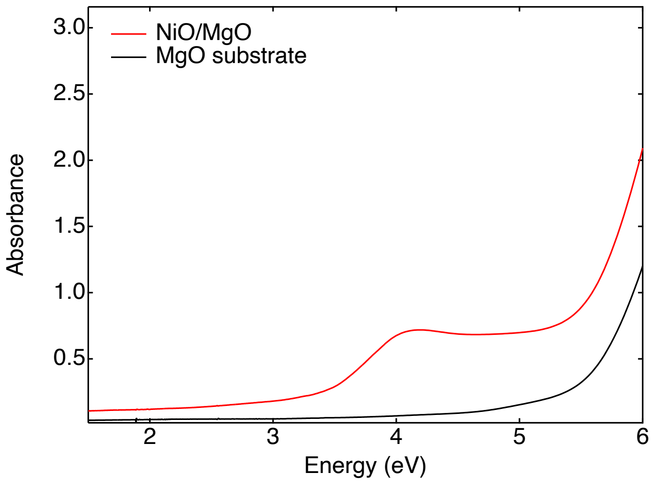

Fig. S6: **UV-Vis spectrum of NiO (001) thin film.** UV-Vis spectrum of NiO (001) thin film on MgO substrate (red curve). The spectrum of the substrate is shown with a black curve for reference.

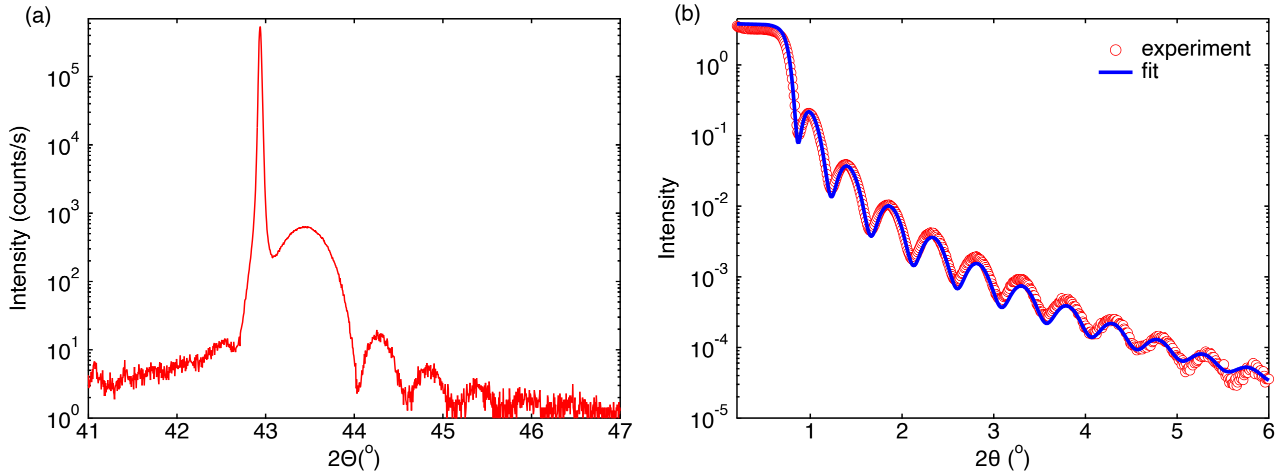

Fig. S7: **Epitaxial film orientation and film thickness.** (a) High-resolution XRD pattern of the NiO thin film near the 002 reflection of MgO. (b) X-ray reflectivity of the NiO thin film.

1.4 X-ray diffraction

X-ray diffraction (XRD) data of NiO (001) single crystal from the same crystal grower (Mateck GmbH) has been previously published in reference (*59*). The diffraction pattern exhibits narrow reflexes related to the (100) Miller planes. The position of the 200 diffraction peak gives a spacing of $d_{200} = 2.09$ Å between Miller planes, corresponding to a lattice parameter of 4.18 Å, close to the reported bulk values between 4.17 and 4.18 Å for antiferromagnetic NiO at room temperature (*65–69*).

High-resolution XRD and XRR of the NiO thin film were measured on a Bruker D8 Discover equipped with high resolution optics in a symmetric scattering geometry. Diffraction pattern near the NiO 002 reflection is shown in Figure S7a. The finite thickness Pendellosung oscillations around the 002 MgO diffraction peak indicate the high quality of the epitaxial NiO thin film. XRR is shown in Figure S7b (red circles). The fitting of the Kiessig fringes provides a film thickness of 17.6 nm, a surface roughness of 0.015 nm and a substrate roughness of 0.45 nm (blue curve in Figure S7b).

1.5 Photoluminescence

Photoluminescence spectra of NiO (001) single crystal used in this work have been previously recorded at 10 K (*59*). Upon excitation below the charge-transfer gap (3.81 eV), two emission peaks are observed at 2.65 and 3.2 eV whereas excitation above the charge-transfer gap (5.04 eV) gives a low energy emission at 2.45 eV and two components at 3.24 and 3.33 eV. The emission lines are assigned to different defect-assisted recombination processes involving trapped carriers and bound excitons.

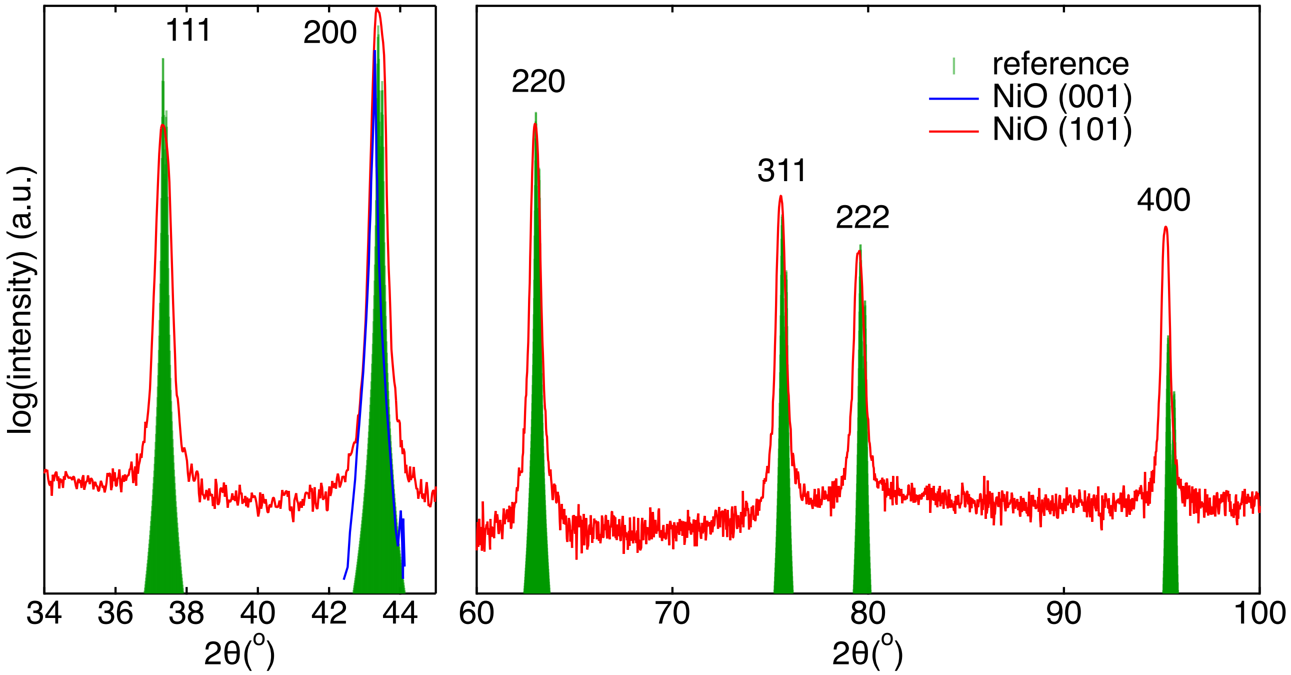

Fig. S8: **XRD patterns of NiO single crystals.** XRD pattern of NiO (100) (blue curve, adapted from (*59*)) and a crushed powder of NiO (101) single crystal (red curve). Comparison is with a standard diffraction pattern from reference (*70*) (green shaded area).

1.6 Calculations of point defect concentrations

In this section, we estimate the point defect concentration of the different samples investigated in this work, and compare it with the excitation density achieved in the transient optical measurements.

1.6.1 NiO thin film

Resistivity measurements from a simple resistance meter maxed out at $\rho > 10\,\mathrm{M\Omega}$, corresponding to a minimum resistivity of $10\,\Omega\,\mathrm{cm}$. This value is an order of magnitude lower than slightly non-stoichiometric NiO thin films deposited on glass substrates by radiofrequency sputtering under oxygen flow (*71*). Assuming that NiO is unintentionally p-doped, and using the reported hole mobility of $\mu = 3 \times 10^{-4}\,\mathrm{cm^2\,V^{-1}\,s^{-1}}$ in NiO thin films (*72*), we find an estimate of the point defect concentration $p = 1/(\rho q \mu)$ with q the electric charge of $p = 2.1 \times 10^{15}\,\mathrm{cm^{-3}}$, which is three orders of magnitude lower than the minimum excitation density achieved in the transient optical measurements on NiO thin films (Figures 3 and 4 of the main text). It means that point defects cannot significantly influence the dynamics of charge carriers as these states become saturated by a small fraction of the photoexcited carrier density.

1.6.2 NiO single crystals

The NiO (001) single crystal was purchased from Mateck GmbH and has been extensively characterized in the literature as a reference NiO sample with a low concentration of point defects. The reported electric conductivity is $2.4 \times 10^{-6}\,\mathrm{S\,m^{-1}}$ (*59*). To estimate the point defect concentration in the crystal, we assume that NiO is unintentionally p-doped due to nickel vacancies, leading to electrical conductivity primarily due to hole conduction. Reported experimental hole mobilities in NiO single crystals are $\mu = 0.3\,\mathrm{cm^2\,V^{-1}\,s^{-1}}$ (*73*), which is usually in the range 0.1–$1\,\mathrm{cm^2\,V^{-1}\,s^{-1}}$. We calculate the density of hole donor sites given by $p = \sigma/(q\mu)$ with q the electric charge, yielding $p = 5 \times 10^{17}\,\mathrm{cm^{-3}}$, representing $0.3\,\%$ of the minimum excitation density used in the measurements in NiO single crystals (Table S8). This defect concentration being significantly lower than the excitation density, it is unlikely to affect the dynamics of charge carriers.

The same arguments hold for NiO (101) single crystals grown by CVT, with no observable spectral signature of long-lived trapped carriers below the NiO CT gap (Figure S31).

2 Interfacial heat transfer, and heat diffusion

2.1 Interfacial heat transfer dynamics

Interfacial heat transfer between the NiO layer and the substrate is simulated for the transient optical measurements on NiO thin films. The evolution of the film temperature T_f with a thickness l in contact with the MgO substrate at the temperature T_s is modeled using the differential equation (*74*),

$$l \cdot C(T_f) \cdot \frac{dT_f}{dt} = -\sigma_K \cdot (T_f - T_s) + \dot{S} \tag{S4}$$

with $C(T_f)$ the specific heat capacity of NiO, σ_K the Kapitza interface conductance (*75*), and $\dot{S}$ the differential of the source term from the laser pump pulse. The substrate lattice temperature is kept constant at room temperature (298 K) due to its large volume and its large thermal conductance ($\sim 50\,\mathrm{W\,m^{-1}\,K^{-1}}$). The source term is made of a gaussian pulse with a FWHM of 1 ps, which corresponds to the typical time of energy transfer between the charge carriers and the lattice. The amplitude of the pulse is made such that the maximum temperature reaches 420 K, corresponding to an estimate of the lattice temperature at 500 fs in the transient optical experiment of Figure 2b in the main text (red curve) and the magnitude of difference spectra upon lattice heating obtained by spectroscopic ellipsometry (light grey). There is no reported value for the Kapitza interface conductance of NiO/MgO(001) and very few at the interface between metal oxides in general. We took, as a starting point, the reported value of $\sigma_K \sim 500\,\mathrm{MW\,m^{-2}\,K^{-1}}$ at the TiN/MgO(001) interface (*76*), since it corresponds to the interface between two materials with rock-salt structures (but with metallic-covalent bonds). We reduced this value by a factor 5 to become $\sigma_K \sim 100\,\mathrm{MW\,m^{-2}\,K^{-1}}$, which is based on the fact that the NiO/MgO interface is between two ionic materials and with a larger mismatch of Debye temperatures than with the TiN layer. The heat capacity of NiO is modeled using a Shomate-type polynomial function $C(T) = a + bT + cT^2 + dT^3$, with $a = 35\,\mathrm{J\,mol^{-1}\,K^{-1}}$, $b = 1.1 \times 10^{-2}\,\mathrm{J\,mol^{-1}\,K^{-2}}$, $c = 2 \times 10^{-6}\,\mathrm{J\,mol^{-1}\,K^{-3}}$, and $d = 5 \times 10^{-10}\,\mathrm{J\,mol^{-1}\,K^{-4}}$. The differential equation S4 is solved with the finite difference method. The evolution of the lattice temperature with the time delay is shown in Figure S9.

The simulation shows that the lattice temperature reached at ~ 1 ns is ~ 310 K, in line with the lattice heating retrieved by transient absorption (Figure 2b in the main text).

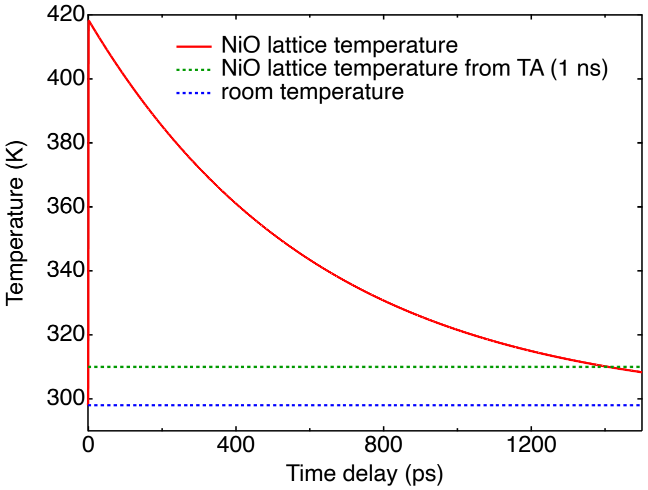

Fig. S9: **Heat diffusion simulation.** Simulation of the NiO lattice temperature in NiO/MgO(001) thin film as a function of time delay due to interfacial heat transfer (red curve). Room temperature (horizontal blue dashed line) and lattice temperature at $1\,\text{ns}$ retrieved from transient absorption in Figure 2b in the main text (green horizontal dashed line) are shown for reference.

2.2 Heat diffusion time

Heat diffusion has been extensively investigated in NiO single crystals. Literature values of the thermal conductivity (λ) are reported in Table S7. The thermal diffusivity D is calculated following,

$$D = \frac{\lambda}{\rho c_p} \tag{S5}$$

with λ the thermal conductivity, ρ the density, and c_p the specific heat capacity. The thermal diffusivity together with a characteristic length scale from the probe beam waist L yield a characteristic timescale τ of heat diffusion away from the excitation volume following $\tau = L^2/D$. The characteristic length scale is taken as the half width at half maximum of the probe beam ($L = 10\,\mu\text{m}$). Depending on the value of the thermal conductivity, the characteristic time of heat diffusion is in the range 3–$11\,\mu\text{s}$.

reference	λ (W m^{-1} K^{-1})	D (m^2 s^{-1})	τ (μs)
(*77*) (experiment)	36.7	9.2×10^{-6}	10.9
(*78*) (experiment)	40	1.0×10^{-5}	10.0
(*79*) (experiment)	22	5.5×10^{-6}	3.0
(*80*) (experiment)	33.8	8.5×10^{-6}	7.2
(*81*) (theory)	24.9	6.2×10^{-6}	3.8
(*82*) (experiment)	29.5	7.4×10^{-6}	5.5

Tab. S7: **Literature values of heat diffusion constants.** Heat diffusion physical parameters of NiO single crystals at room temperature (300 K): thermal conductivity (λ), thermal diffusivity (D). Calculations are performed with tabulated values of the NiO density ($\rho = 6.72$ g cm^{-3}, (*83*)), molecular weight ($M = 74.69$ g mol^{-1}), and specific heat capacity ($c_p = 44.4$ J mol^{-1} K^{-1}, (*84*)). The characteristic length scale for the calculation of τ is $L = 10$ μm.

3 Target analysis

Target analysis of the transient signal is performed with the `Glotaran` code (*85*). We use its python implementation (`PyGlotaran` version 0.7.0) (*86*). The dynamics in NiO can be well reproduced by using two exponential decays (corresponding to the electronic response of NiO) and one slow process with an asymptotically exponential rise (of the type $1 - \exp(-t/\tau)$, corresponding to pure lattice heating) and a fixed 100 ns decay time (corresponding to a timescale of heat diffusion away from the probe volume). The three exponential decays correspond to species 1, 2, and 3 with a maximum concentration reached after the pump pulse has been delivered. These species decay in parallel with the time constants τ_1, τ_2, and τ_3, respectively. The exponential rise corresponds to the formation of specie 4, forming by decay of specie 3 in a sequential model with a rise time τ_3 and a decay time constant τ_4. A coherent artifact of order 3 is used to reproduce the self-phase modulation at time delays < 100 fs but is ignored in the discussion. Its FWHM is set equal to the instrument response function, fixed at 220 fs, corresponding to the cross-correlation between the pump and probe pulses. Due to the lattice heating, NiO is expected to recover to the ground state on a longer timescale than the experimental time window (hence the fixed $\tau_4 = 100$ ns lifetime), which means that some of the heating contribution undergoes a negligible decay over the temporal window of the experiment (~ 1.4 ns). The instrument response function (IRF) is simulated with a gaussian lineshape with a fixed FWHM of 220 fs. No IRF dispersion is included in the fitting since the correction of the probe pulse group velocity dispersion is performed directly on the raw data. The "TrustReflectiveRegion" optimization method is used for the fitting. the convergence criteria of `ftol`, `gtol` and `xtol` are all set to 1×10^{-8}.

4 NiO thin film – Transient transmission

All measurements in this section are performed at room temperature ($300\,\mathrm{K}$). The method used to calculate the excitation density is provided in SI §5.

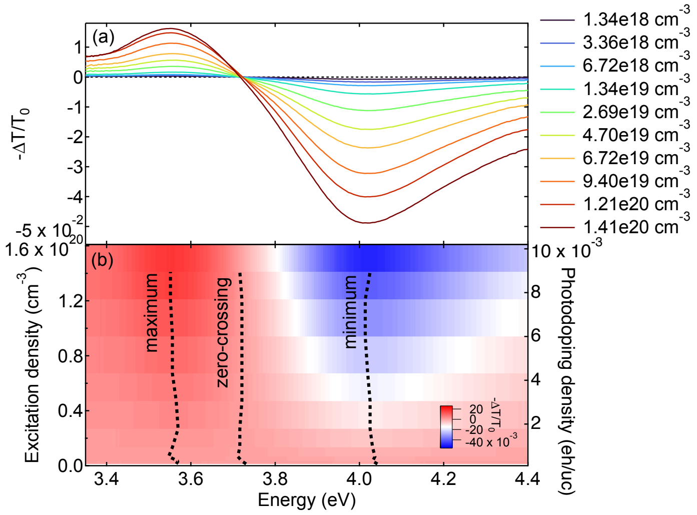

Fig. S10: **Spectral traces with excitation density.** (a,b) Evolution of the spectral traces with the excitation excitation density. In panel (b), the position of the minimum, the zero-crossing, and the maximum amplitudes of the spectral traces are displayed with dashed lines.

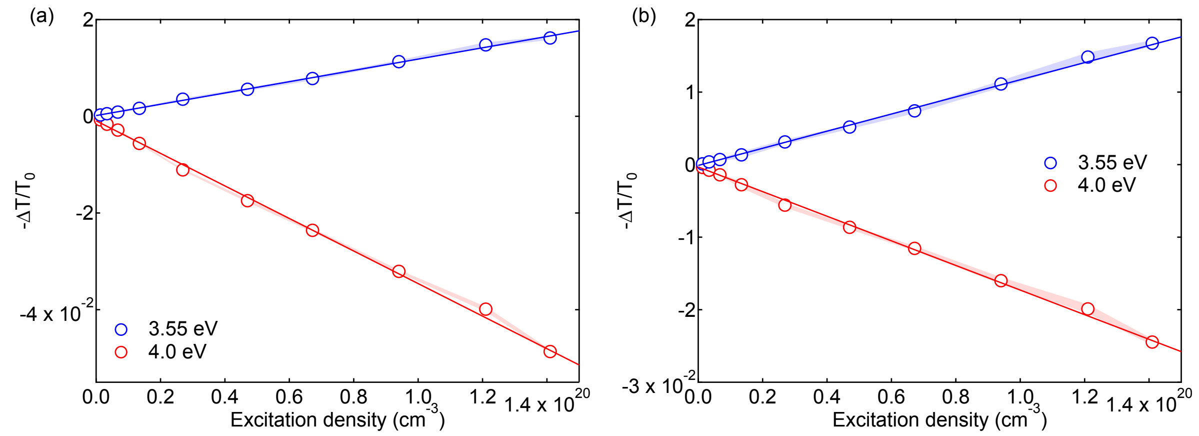

Fig. S11: **Transient amplitude with excitation fluence.** Evolution of the transient amplitude upon excitation at $4.66\,\mathrm{eV}$ at (a) $0.5\,\mathrm{ps}$, and (b) $100\,\mathrm{ps}$ time delay with probe photons of $3.55\,\mathrm{eV}$ (blue circles) and $4.0\,\mathrm{eV}$ (red circles). Shaded areas represent the standard deviation between individual measurements. Linear fits are shown with continuous lines.

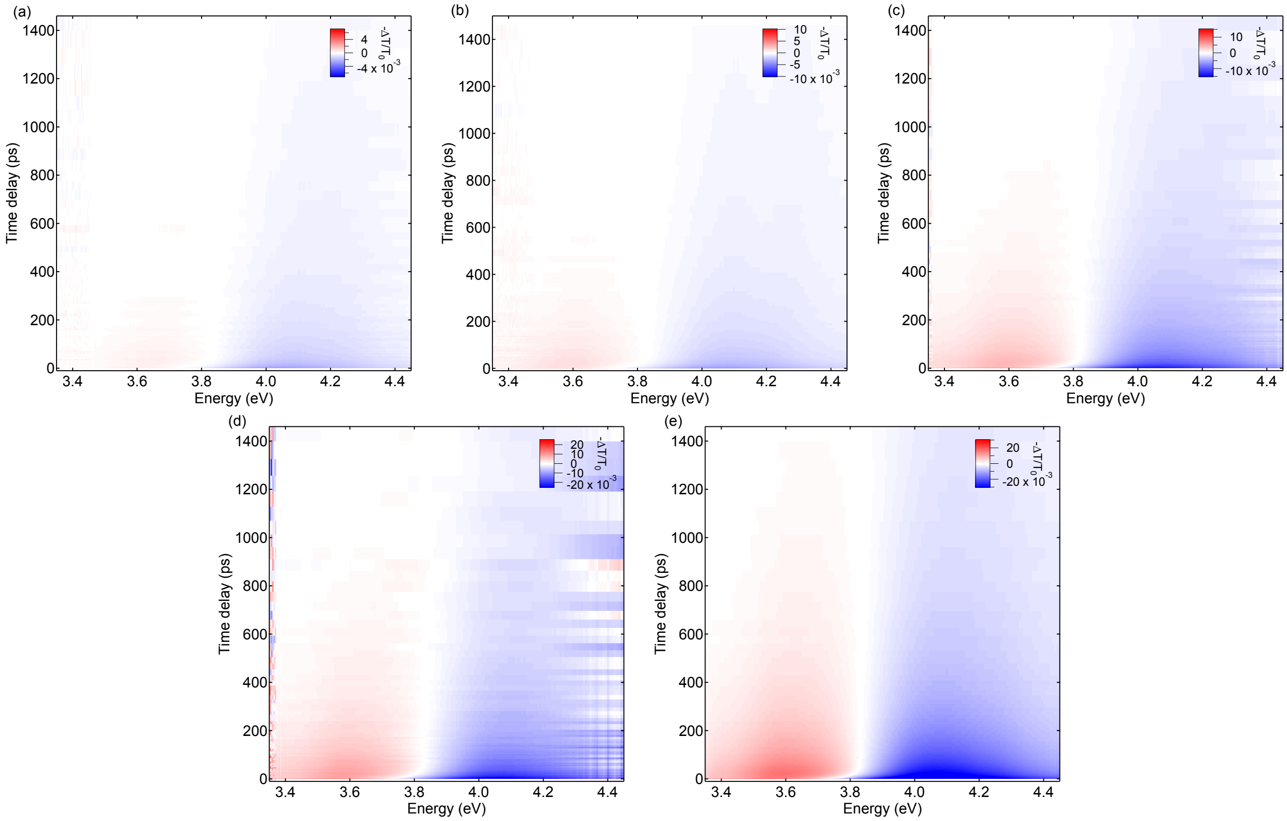

Fig. S12: **Transient transmission with excitation density.** Color-coded maps of the transient transmission with 4.66 eV pump at various excitation densities (a) 5.02×10^{18} cm^{-3} (0.0004 eh/uc), (b) 1.05×10^{19} cm^{-3} (0.0008 eh/uc), (c) 3.77×10^{19} cm^{-3} (0.0027 eh/uc), (d) 7.53×10^{19} cm^{-3} (0.0055 eh/uc), and (e) 1.26×10^{20} cm^{-3} (0.0091 eh/uc).

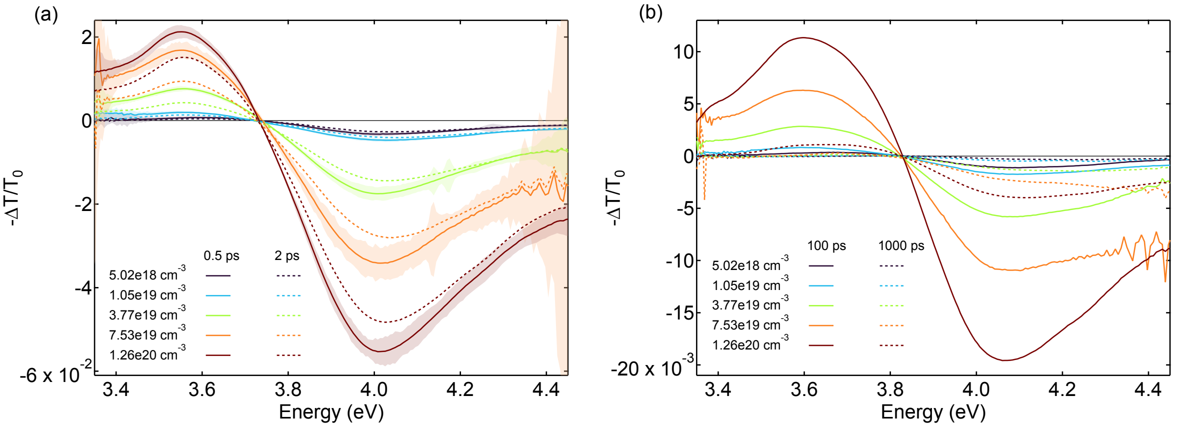

Fig. S13: **Spectral traces with excitation density and time delays.** Spectral traces upon 4.66 eV excitation with various excitation densities: (a) femtosecond/picosecond, and (b) nanosecond time delays. Shaded areas represent standard deviations between individual measurements.

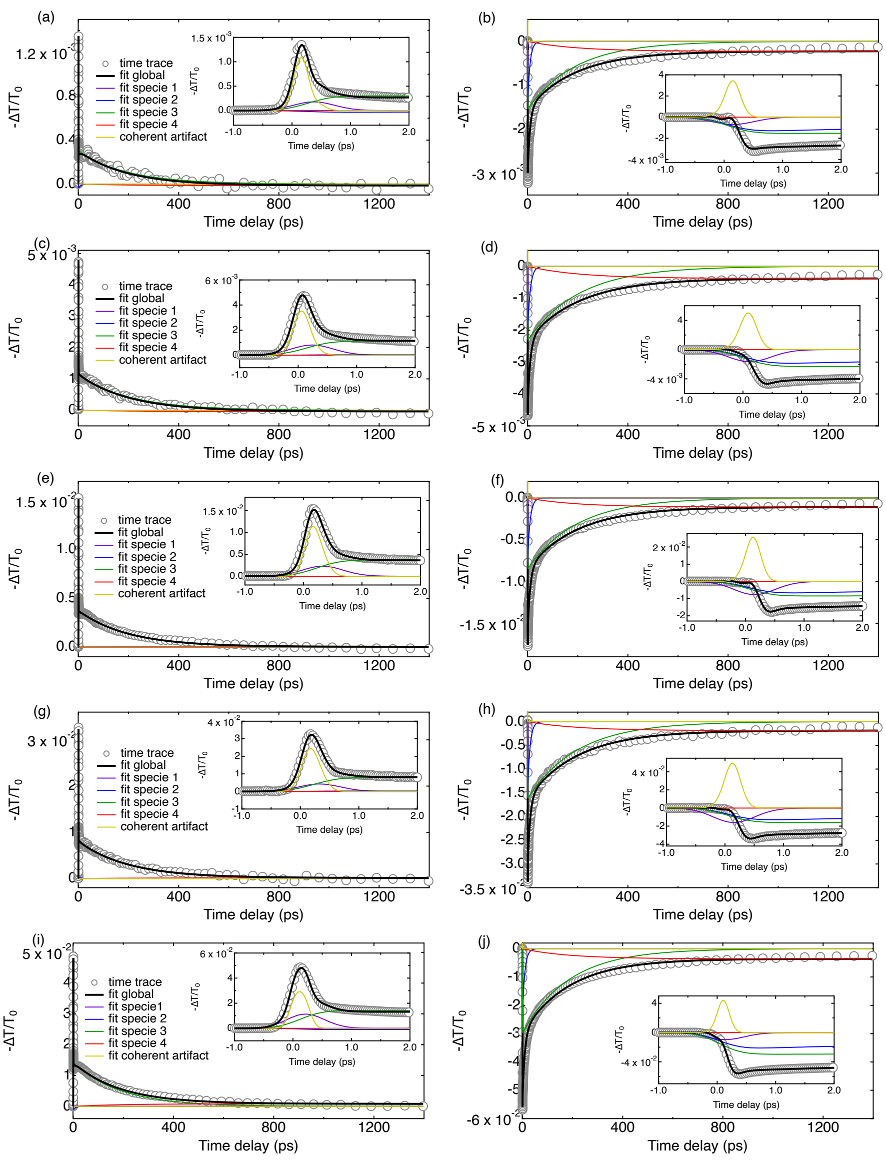

Fig. S14: **Kinetic decomposition of target analysis.** Time traces upon $4.66\,\text{eV}$ photoexcitation at (a,c,e,g,i) $3.55\,\text{eV}$, and (b,d,f,h,j) $4.0\,\text{eV}$ probe photon energies with excitation density (a,b) $5.02\times10^{18}\,\text{cm}^{-3}$, (c,d) $1.05\times10^{19}\,\text{cm}^{-3}$, (e,f) $3.77\times10^{19}\,\text{cm}^{-3}$, (g,h) $7.53\times10^{19}\,\text{cm}^{-3}$, and (i,j) $1.26\times10^{20}\,\text{cm}^{-3}$ (grey circles). The fitted time traces by target analysis (black curves) are decomposed into individual components: coherent artifact (yellow curve), specie associated decay 1 with time constant τ_1 (purple curve), 2 with time constant τ_2 (blue curve), 3 with time constant τ_3 (green curve), and 4 with rise time constant τ_3 and decay time constant τ_4 (red curve).

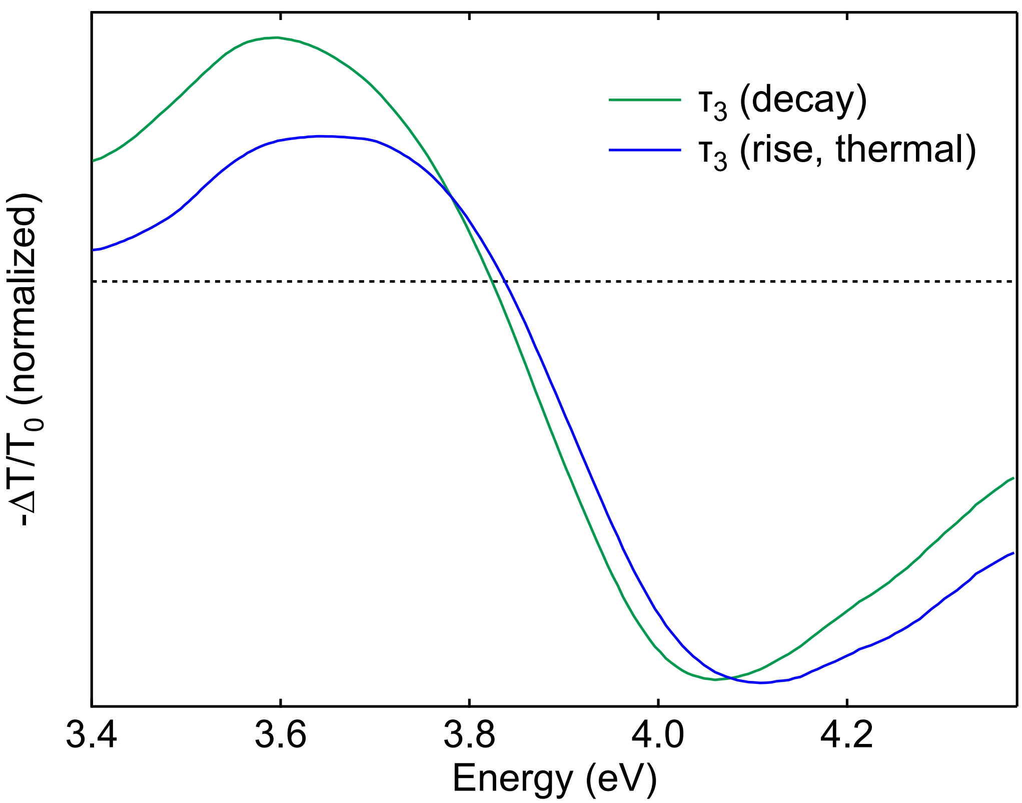

Fig. S15: **Decay associated spectra of long-lived states.** Normalized decay associated spectra (DAS) at the amplitude minimum for the decaying and rising components with time constant τ_3 upon photoexcitation at 4.66 eV with a density 1.26×10^{20} cm^{-3} (0.0091 eh/uc).

Excitation density (cm^{-3})	Excitation density (eh/uc)	τ_1 (ps)	τ_2 (ps)	τ_3 (ps)
5.02×10^{18}	0.0004	$0.10(5)$	$8.6(8)$	$190(32)$
1.05×10^{19}	0.0008	$0.25(4)$	$9.4(7)$	$201(21)$
3.77×10^{19}	0.0027	$0.11(5)$	$8.9(9)$	$189(37)$
7.53×10^{19}	0.0055	$0.12(8)$	$8.2(7)$	$192(24)$
1.26×10^{20}	0.0091	$0.11(1)$	$9.0(7)$	$186(25)$

Tab. S8: **Time constants with excitation density.** Time constants retrieved from the target analysis of NiO (001) thin film measurements in transmission at various excitation densities. Confidence intervals of 95% are calculated from the standard error of the fitting parameters.

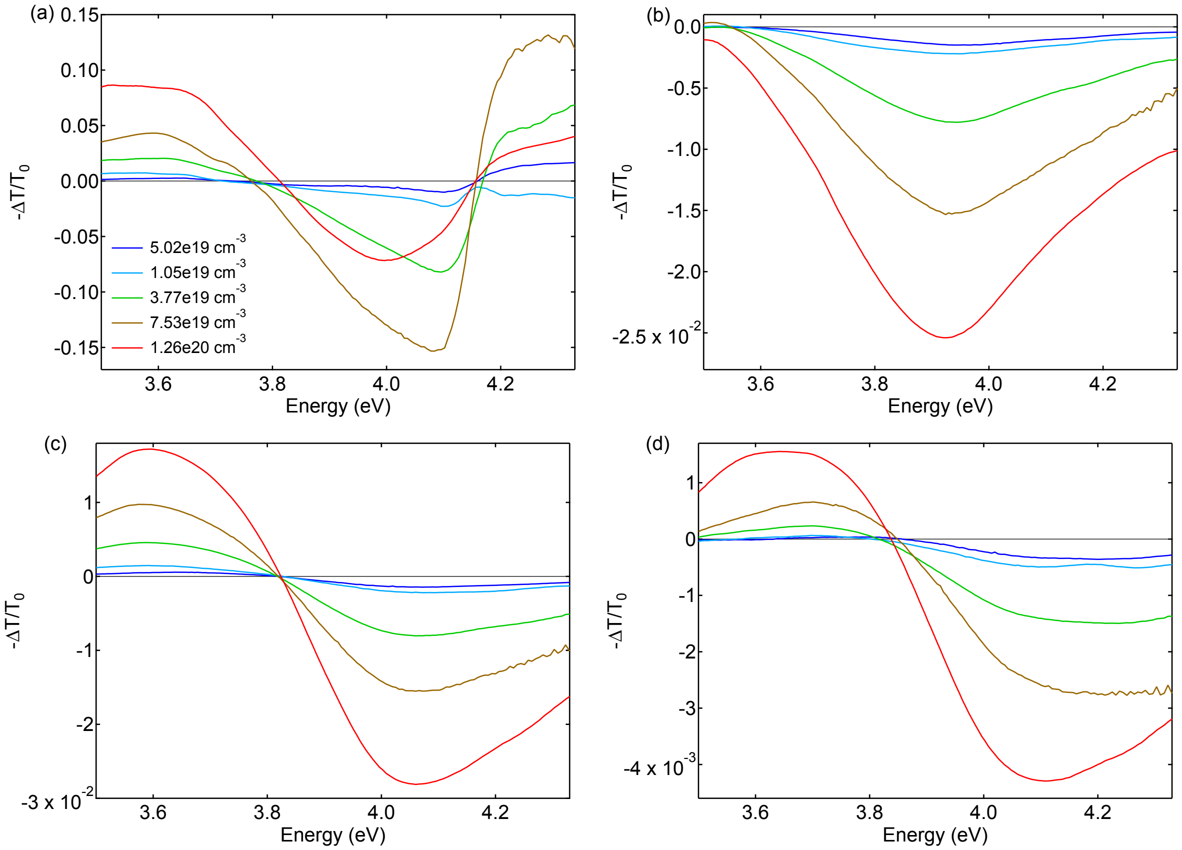

Fig. S16: **Decay associated spectra with excitation density.** Decay associated spectra (DAS) from the target analysis of NiO thin film transient optical measurements in transmission at various excitation densities (pump energy $4.66\,\mathrm{eV}$). The DAS are for the time constants (a) τ_1, (b) τ_2, (c) τ_3 (decay component), and (d) τ_3 (rise component, pure heat signal).

5 Calculation of the initial excitation density

For a bulk sample, the initial excitation density n achieved after the pump laser pulse is delivered to the sample and in the absence of charge-carrier recombination is calculated as,

$$n = (1 - R)\frac{F}{h\nu\lambda_p} \tag{S6}$$

where R is the reflectivity at the pump photon energy, F the laser fluence, $h\nu$ the pump photon energy and λ_p the penetration depth at the pump photon energy. The fluence is given in FWHM unit of the beam waist. The reflectivity and the penetration depth are provided by spectroscopic ellipsometry (see SI §1.3). The absorption of the laser fluence is assumed linear with increasing average pump powers. For a thin sample, when the penetration depth of the pump is similar to the sample thickness, the partial absorption of the laser needs to be considered to calculate the excitation density. In this case, the incident fluence (F_0) is corrected to an effective absorbed fluence (F) following the relation $F = F_0(1 - e^{-t/\lambda_p})$ with t the sample thickness determined by the Laue fringes in XRD (see SI §1.4).

The conversion between the experimental average pump powers, the excitation density and the number of electron-hole pair excitations per unit cell is given in Table S9 for the NiO thin film and Table S10 for the NiO single crystals. For the calculation of the number of excitations per unit cell, a lattice parameters of $4.1684\,\text{Å}$ is assumed at room temperature, which corresponds to the bulk value (*67*).

Pump power (μW)	Excitation density (cm^{-3})	Excitation density (eh/uc)
20	1.34×10^{18}	> 0.0001
50	3.36×10^{18}	0.0002
100	6.72×10^{18}	0.0005
200	1.34×10^{19}	0.0010
300	2.01×10^{19}	0.0015
400	2.69×10^{19}	0.0019
600	4.03×10^{19}	0.0029
700	4.70×10^{19}	0.0034
800	5.37×10^{19}	0.0039
1000	6.72×10^{19}	0.0049
1250	8.40×10^{19}	0.0061
1400	9.40×10^{19}	0.0068
1500	1.01×10^{20}	0.0073
1800	1.21×10^{20}	0.0088
2100	1.41×10^{20}	0.0102
2150	1.44×10^{20}	0.0104
2450	1.65×10^{20}	0.0120
2500	1.68×10^{20}	0.0122

Tab. S9: **Excitation levels for NiO thin film.** Conversion between the average pump power, the initial excitation density and the number of excitation per unit cell for the pump-probe measurements on NiO thin film (pump photon energy is 4.66 eV).

Pump power (μW)	Excitation density (cm^{-3})	Excitation density (eh/uc)
90	1.5×10^{20}	0.0109
135	2.1×10^{20}	0.0152
195	3.0×10^{20}	0.0217

Tab. S10: **Excitation levels for NiO single crystals.** Conversion between the average pump power, the initial excitation density and the number of excitation per unit cell for the pump-probe measurements on NiO single crystals. The pump photon energy is 4.28 eV.

6 NiO thin film – Transient reflectivity

In this section, we investigate the transient reflectivity of NiO thin films upon photodoping above the CT gap. Figure S17 displays color-coded map of the transient reflectivity at various excitation densities. Spectral traces at $500\,\text{fs}$ are shown in Figure S18a. Similarly to the measurements in transmission, the transient amplitude displays a linear increase with the excitation density (Figure S18b). The spectral traces in transmission and reflectivity are qualitatively related by Kramers-Kronig transformation (Figure S19). The normalized kinetics remain unchanged between the minimum ($1.13 \times 10^{20}\,\text{cm}^{-3}$, 0.0008 eh/uc) and maximum ($1.84 \times 10^{20}\,\text{cm}^{-3}$, 0.013 eh/uc) excitation densities (Figure S20). The same target analysis model is used as for the measurement in transmission. No self-phase modulation appears in transient reflectivity and thus this contribution is removed from the model. and the DAS at a selected excitation density is shown in Figure S21. The DAS of τ_2 and τ_3 (decay and rise) have resonance lineshapes with a negative amplitude centered at ~ 3.65 and $\sim 3.95\,\text{eV}$, respectively. The time constants obtained at other excitation densities are given in Table S11. Since the DAS of τ_2 and τ_3 (decay) are spectrally distinct with different time constants, it shows that the two local minima in the spectral traces of Figure S18a decay at different rates with a faster decay on the low energy side of the CT gap. The DAS of τ_3 (rising component) can be reproduced with simulated transient reflectivity signals of NiO hot lattices (black circles in Figure S21). Since τ_3 yields consistent values in the range 180–$230\,\text{ps}$ in transmission and in reflectivity, lattice heating can be considered as the dominant contribution to the transient signal for time delays $\gtrsim 100\,\text{ps}$. We note that the confidence intervals in the target analysis of the transient reflectivity signals of the thin films are slightly worse than for the measurements in transmission, which is a combination of larger standard deviations in the reflectivity measurements (higher noise levels) and because the target analysis would likely require a more complex model to account for the simultaneous changes in the real and imaginary parts of the refractive index (*87, 88*).

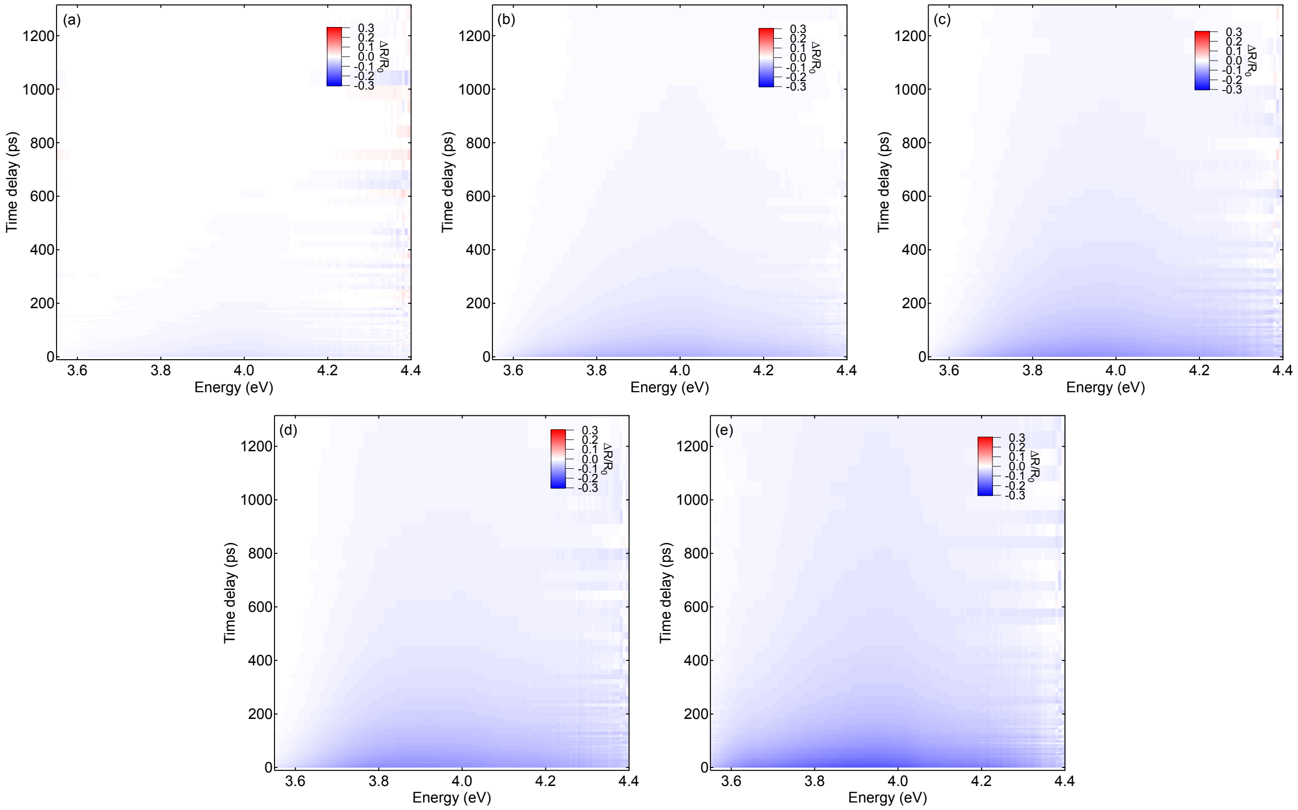

Fig. S17: **Transient reflectivity on NiO thin film.** Color-coded maps of the transient reflectivity on NiO thin film after excitation at $4.66\,$eV with excitation densities: (a) 1.13×10^{19} cm^{-3} (0.0008 eh/uc), (b) 7.37×10^{19} cm^{-3} (0.0053 eh/uc), (c) 1.13×10^{20} cm^{-3} (0.0082 eh/uc), (d) 1.23×10^{20} cm^{-3} (0.0089 eh/uc), and (e) 1.84×10^{20} cm^{-3} (0.0133 eh/uc).

Excitation density (cm^{-3})	Excitation density (eh/uc)	τ_1 (ps)	τ_2 (ps)	τ_3 (ps)
7.37×10^{19}	0.005	0.7(1)	10.2(5)	220(42)
1.13×10^{20}	0.008	0.7(1)	9.8(8)	190(32)
1.23×10^{20}	0.009	0.7(1)	9.4(7)	201(21)
1.84×10^{20}	0.013	0.6(1)	9.0(7)	186(25)

Tab. S11: **Time constants of target analysis on NiO thin film.** Time constants retrieved from the target analysis of NiO (001) thin film measurements in reflectivity at various excitation densities (pump excitation at $4.66\,$eV). Confidence intervals in the 95% range are calculated from the standard error of the fitting parameters.

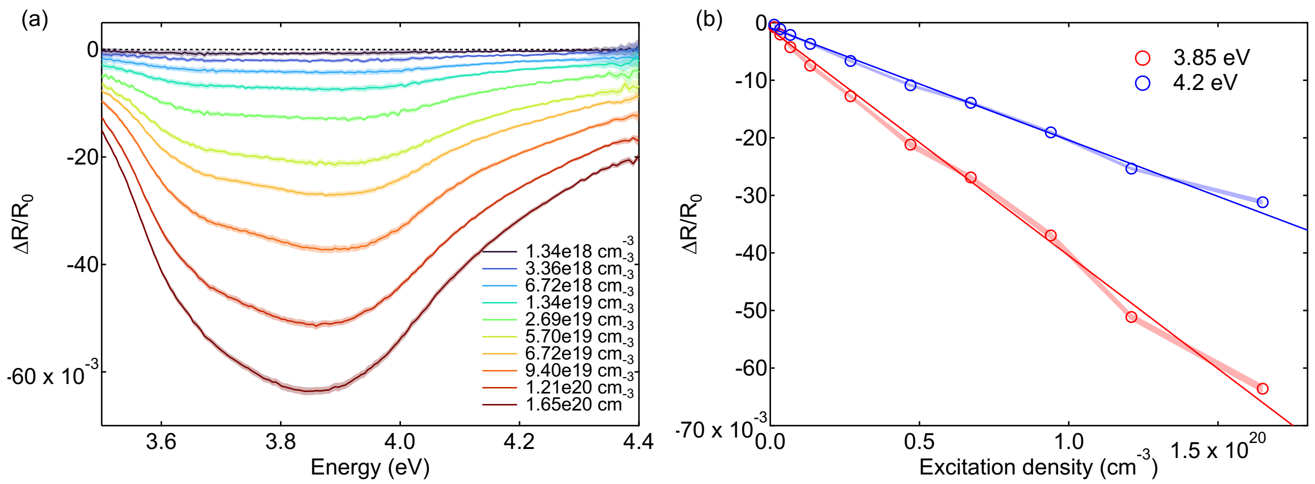

Fig. S18: **Spectral traces and amplitude of transient reflectivity on NiO thin film.** (a) Spectral traces in transient reflectivity at $500\,$fs at different excitation densities (pump excitation at $4.66\,$eV). (b) Linearity of the transient reflectivity amplitude at probe energies $3.85\,$eV (red circles) and $4.2\,$eV (blue circles) and at $500\,$fs time delay. Continuous lines are linear fittings to the experimental points. Shaded areas represent standard deviations between individual measurements.

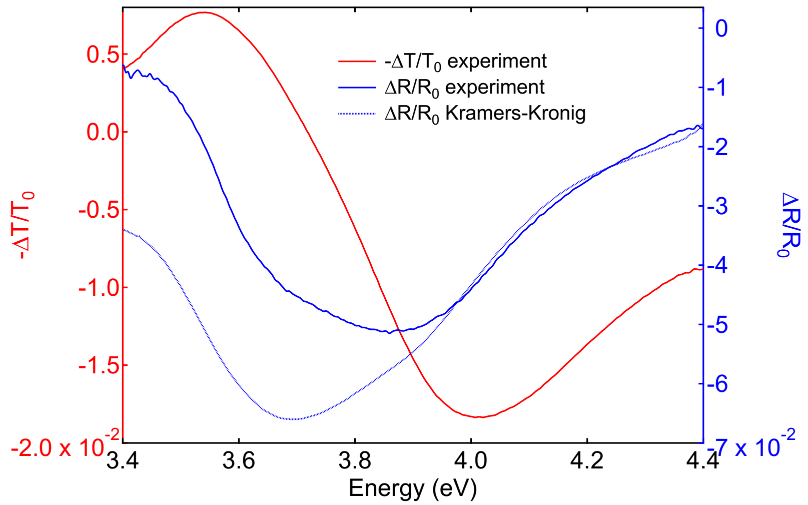

Fig. S19: **Transient transmission/reflectivity and Kramers-Kronig transform.** Transient transmission (continuous red curve) and transient reflectivity (continuous blue curve) of NiO thin film (pump $4.66\,$eV, time delay $2\,$ps, excitation density $\sim 4\times10^{19}\,\mathrm{cm}^{-3}$). Result of the Kramers-Kronig transformation of the transient transmission is shown with a dotted blue line.

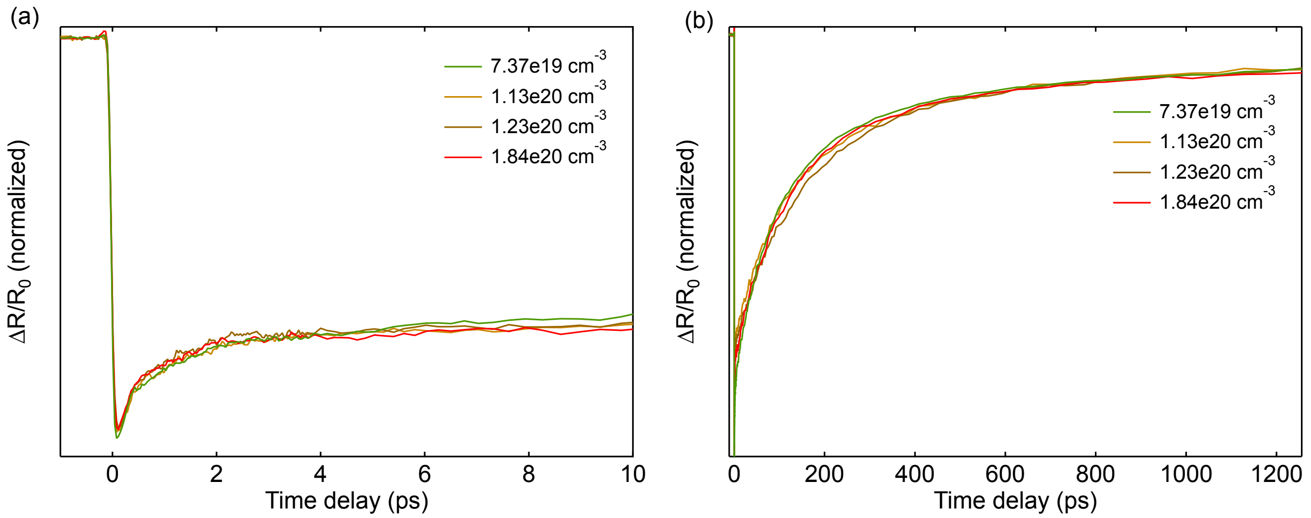

Fig. S20: **Normalized transient reflectivity kinetics of NiO thin film.** Normalized time traces measured on NiO thin film in transient reflectivity at 3.9 eV probe energy on the (a) femtosecond/picosecond timescale, and (b) on the nanosecond timescale at various excitation densities (pump 4.66 eV). The excitation densities are 7.37×10^{19} cm^{-3} (0.0053 eh/uc), 1.13×10^{20} cm^{-3} (0.0082 eh/uc), 1.23×10^{20} cm^{-3} (0.0089 eh/uc), and 1.84×10^{20} cm^{-3} (0.0133 eh/uc)

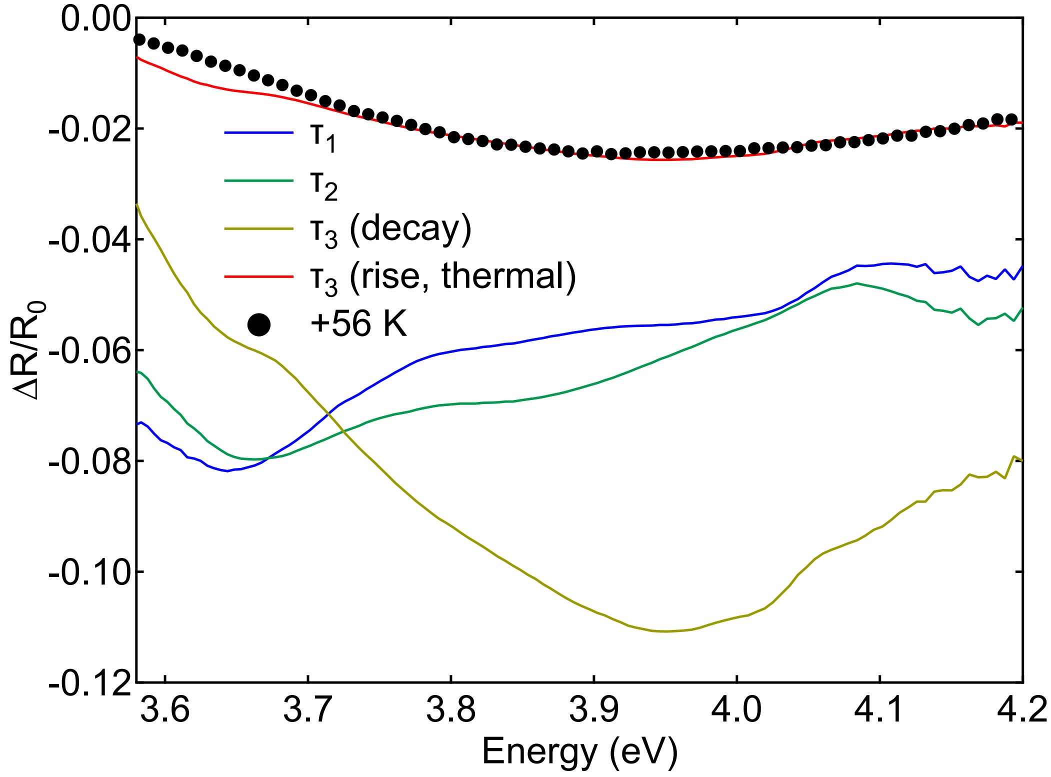

Fig. S21: **Decay associated spectra of target analysis.** Decay associated spectra (DAS) from the target analysis of the transient reflectivity with 4.66 eV photoexcitation at an excitation density of 1.84×10^{20} cm^{-3} (0.013 eh/uc). The decay time constants are $\tau_1 = 0.7(1)$ ps, $\tau_2 = 9.8(8)$ ps, and $\tau_3 = 229(65)$ ps. The black circles show a simulated transient reflectivity spectrum for a NiO lattice heated up by 56 K from room temperature.

7 NiO single crystals – Transient reflectivity

The effect of the excitation density with 4.28 eV pump photon energy on NiO (100) single crystals is investigated by transient reflectivity (Figure S22). The transient displays a main negative signal centered at 3.9–4.0 eV, similar to the transient reflectivity measurements on NiO thin films (Figure S17). Akin, the kinetics do not depend on the excitation density between 1.5×10^{20} $(0.011$ eh/uc$)$ and 3.0×10^{20} cm^{-3} $(0.022$ eh/uc$)$ with 4.28 eV pump photons (Figure S23, measurements at long time delay are shown in Figure S24). The spectral traces exhibit slightly different lineshapes than for the transient reflectivity measurements on NiO thin films (Figure S25a and S18a), which is attributed to optical effects related to the sample thickness in the measurement on thin films. Upon lattice heating, a negative transient signal is expected on the high energy side of the probed spectral window based on previous thermoreflectance measurements on NiO single crystals (*89–91*), consistent with the spectral traces at long time delays (SI Figure S25b). A detailed comparison between spectral traces and simulated transient reflectivity signals from lattice heating (*19*) (details in SI §1.3) shows that the latter fully reproduces the transient signal at 1.4 ns (red curve and black squares in Figure S26a). Thermal contributions are likely present in the transient signal at shorter time delays, similarly to the thin film measurements. However, large deviations are found between the transient and the hot lattice simulation at 0.5 ps (purple curve and black triangles in Figure S26a), which indicates that at short time delays, the transient is most likely non-thermal. A negative transient signal centered at ~ 4.0 eV at $\lesssim 100$ ps time delays agrees with previous electroreflectance measurements (*92*) (Figure S26b), indicating that the lineshape is compatible with an electronic response.

The effect of the pump photon energy, between 3.54 and 4.28 eV, is also investigated on NiO (001) single crystals with comparable excitation densities ($\sim 2.5 \times 10^{20}$ cm^{-3}, 0.018 eh/uc). Color-coded maps of the measurements are shown in Figure S27 for pump excitations at 3.54 and 3.97 eV. The transient reflectivity conserves a similar lineshape, essentially negative at < 10 ps time delays, which is similar to the measurement with 4.28 eV pump photon energy (Figure S22). The kinetics show only a weak dependence on the pump photon energy between 3.97 and 4.28 eV (Figure S28). However, the decay becomes much slower at a pump photon energy of 3.54 eV, which corresponds to the onset of the optical absorption edge (see absorption coefficient in Figure S2a). A stronger negative signal on the high energy side of the probe window seems to correlate with an increase of the pump photon energy (Figure S29a). The orientation of the NiO single crystal surface ((001) or (100)) has no clear effect on either the transient spectral lineshape (Figure S29b) or the kinetics (Figure S30) within the

standard deviation at a pump photon energy of $4.28\,$eV. Transient reflectivity measurements with a Visible probe below the charge-transfer gap show a short-lived signal decaying with time time constants $\tau_1 = 0.5(1)$ ps and $\tau_2 = 3(1)$ ps (Figure S31). The time constants τ_2 is in line with the decay time of photoexcited carriers stabilized at shallow trap states (*93*).

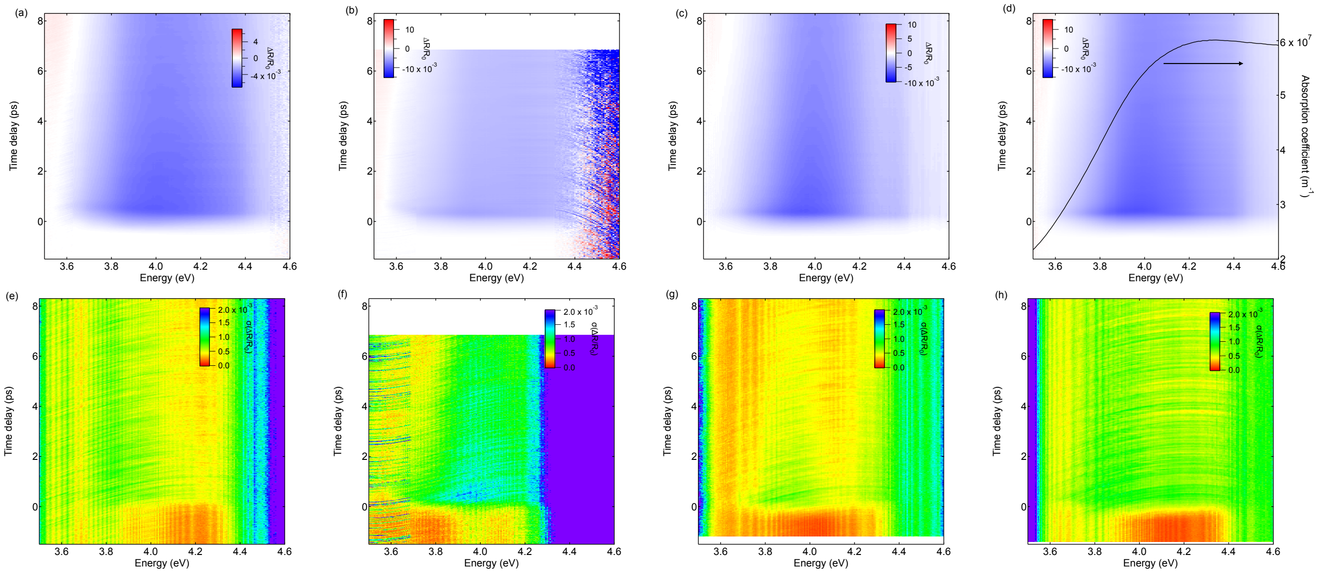

Fig. S22: **Transient reflectivity of NiO single crystal.** Transient reflectivity $\Delta R/R_0$ of NiO (100) single crystal after photoexcitation at $4.28\,$eV with excitation densities of (a) $1.5 \times 10^{20}\,$cm^{-3} (0.011 eh/uc), (b) $1.7 \times 10^{20}\,$cm^{-3} (0.012 eh/uc), (c) $2.1 \times 10^{20}\,$cm^{-3} (0.015 eh/uc), and (d) $3.0 \times 10^{20}\,$cm^{-3} (0.022 eh/uc). (e,f,g,h) Standard deviations of the transient reflectivity signals ($\sigma(\Delta R/R_0)$) plotted directly above. The absorption coefficient is shown in (d) for reference (black curve, right axis).

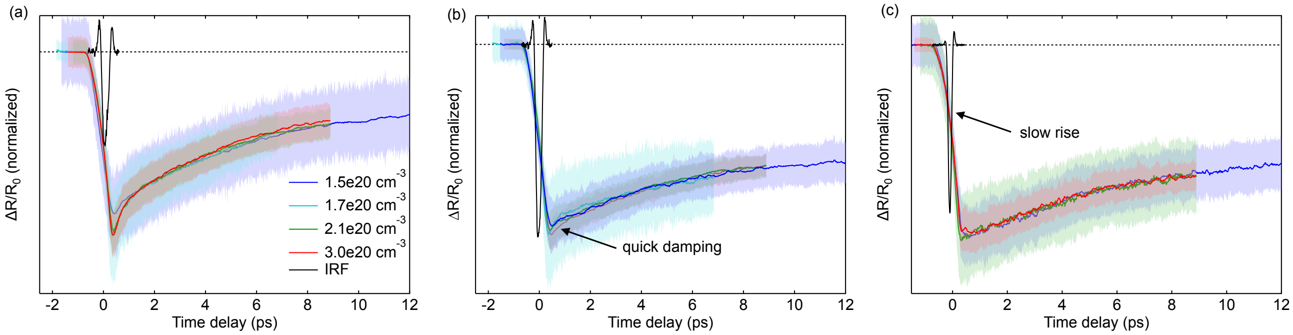

Fig. S23: **Normalized transient reflectivity kinetics of NiO single crystal.** Normalized time traces after photoexcitation at 4.28 eV with (a) 3.8 eV, (b) 4.0 eV, and (c) 4.3 eV probe photon energy at increasing excitation densities: 1.5×10^{20} cm^{-3} (0.011 eh/uc, blue curve), 1.7×10^{20} cm^{-3} (0.012 eh/uc, cyan curve), 2.1×10^{20} cm^{-3} (0.015 eh/uc, green curve), and 3.0×10^{20} cm^{-3} (0.022 eh/uc, red curve). The instrument response function (IRF) at each probe photon energy is illustrated with a self-phase modulation signal (black curve). Shaded areas represent standard deviations between individual measurements.

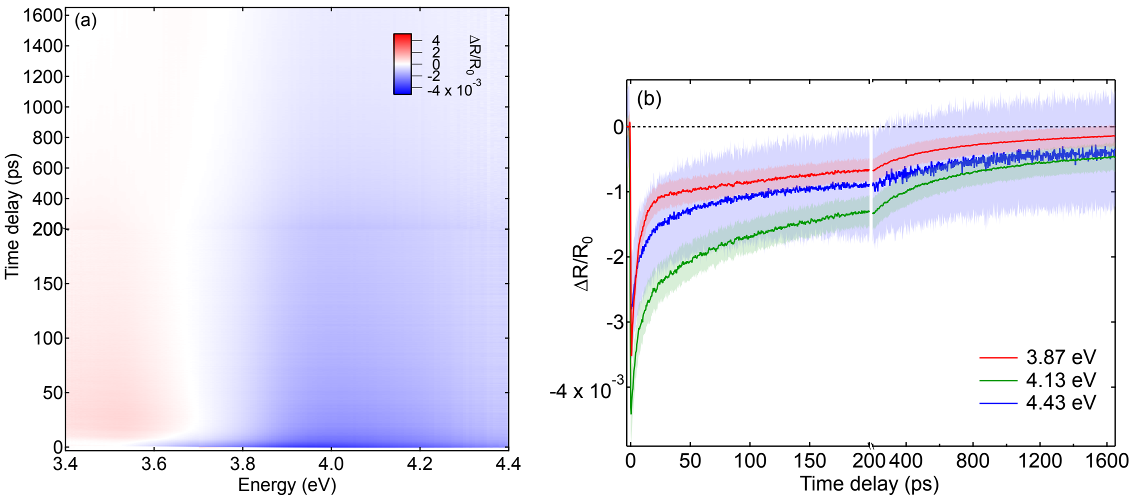

Fig. S24: **Transient reflectivity and kinetics at various probe photon energies.** (a) Color-coded map of transient reflectivity and (b) time traces on the several 100 ps and nanosecond timescale in NiO (001) with 4.28 eV photodoping with excitation density 2.5×10^{20} cm^{-3} (0.018 eh/uc) at probe photon energy 3.87 eV (red curve), 4.13 eV (green curve), and 4.43 eV (blue curve).

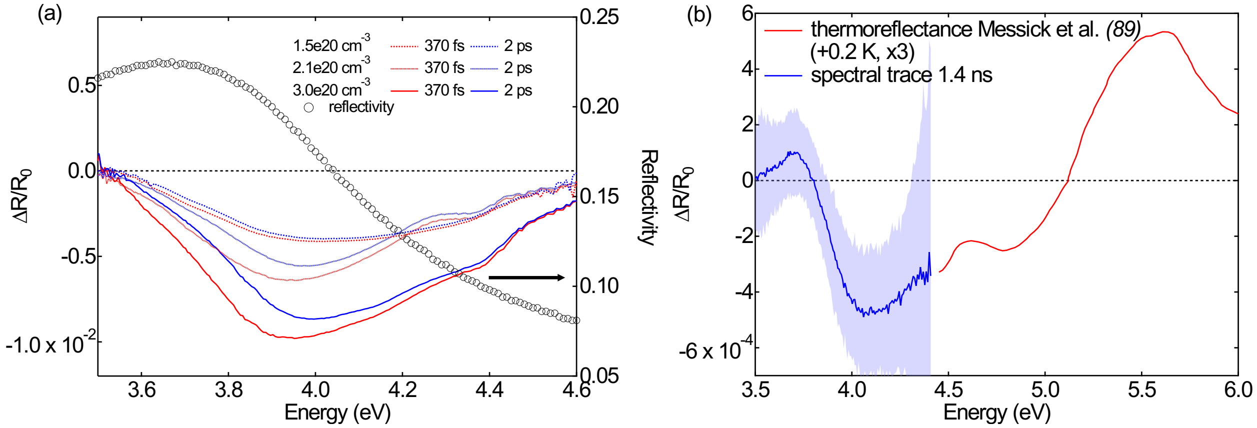

Fig. S25: **Spectral traces and thermoreflectance.** (a) Spectral traces upon photodoping at $4.28\,\mathrm{eV}$ with increasing excitation densities at $370\,\mathrm{fs}$ (red curves, left axis) and $2\,\mathrm{ps}$ (blue curves, left axis). The reflectivity (black circles, right axis) is shown for reference. (b) Comparison between a spectral trace at $1.4\,\mathrm{ns}$ time delay in transient reflectivity (pump $4.28\,\mathrm{eV}$, excitation density $2.5 \times 10^{20}\,\mathrm{cm}^{-3}$, $0.018\,\mathrm{eh/uc}$, blue curve) and scaled relative reflectivity difference from thermoreflectance (red curve), adapted from (*89–91*). The shaded area represents the standard deviation between individual measurements.

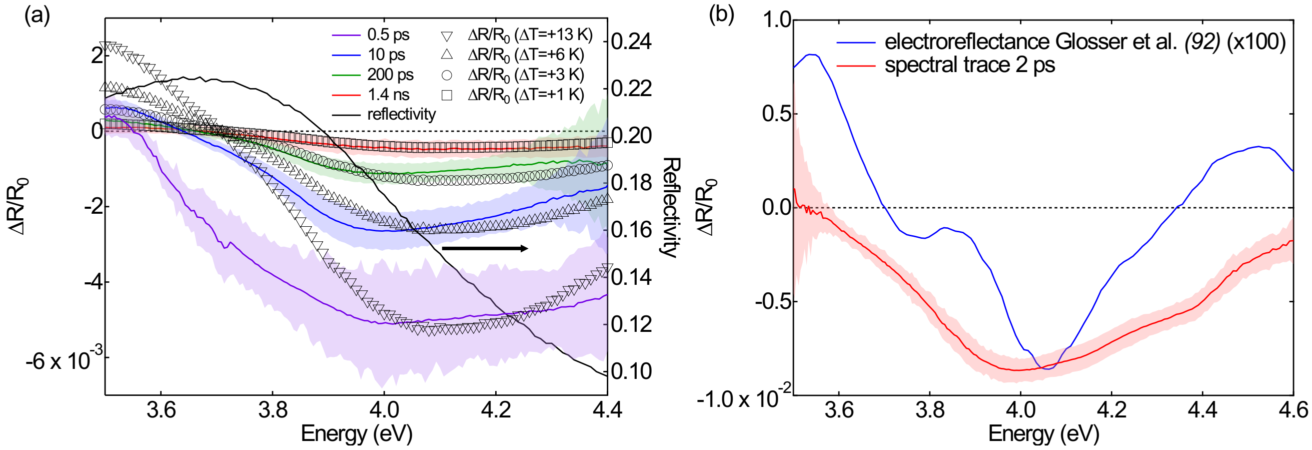

Fig. S26: **Spectral traces and electroreflectance.** (a) Transient reflectivity spectral traces of NiO (001) single crystal with $4.28\,\mathrm{eV}$ pump photons at an excitation density of $2.5 \times 10^{20}\,\mathrm{cm}^{-3}$ (0.018 eh/uc, colored curves, left axis). Simulated transient reflectivity upon lattice heating are shown (black markers, adapted from (*19*), left axis). Shaded areas represent the standard deviation between individual transient reflectivity measurements. The reflectivity (black curve, right axis) is shown for reference. (b) Comparison between a spectral trace at $2\,\mathrm{ps}$ time delay in transient reflectivity (pump $4.28\,\mathrm{eV}$, excitation density $3.0 \times 10^{20}\,\mathrm{cm}^{-3}$, 0.022 eh/uc, red curve) and scaled relative reflectivity difference from electroreflectance (blue curve), original data from (*92*). The shaded are represents the standard deviation between individual measurements.

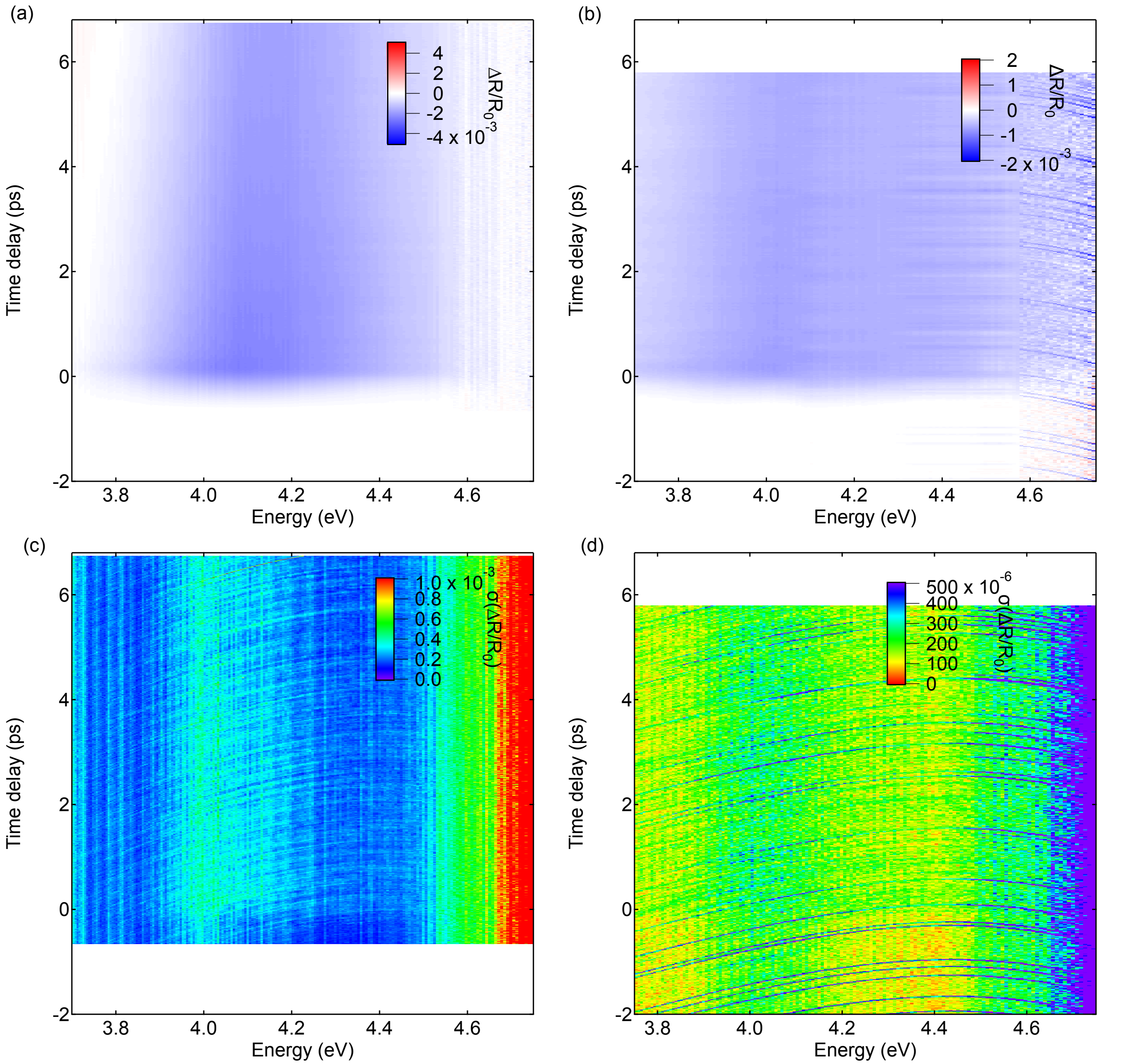

Fig. S27: **Effect of pump photon energy on transient reflectivity of NiO (001) single crystal.** Transient reflectivity of NiO (001) single crystal following excitation with (a) $3.97\,$eV, and (b) $3.54\,$eV pump photon energies (excitation density $\sim 2.5 \times 10^{20}\,$cm^{-3}, ~ 0.018 eh/uc). Standard deviation for the corresponding transient reflectivity signals ($\sigma(\Delta R/R_0)$) are given in (c) and (d).

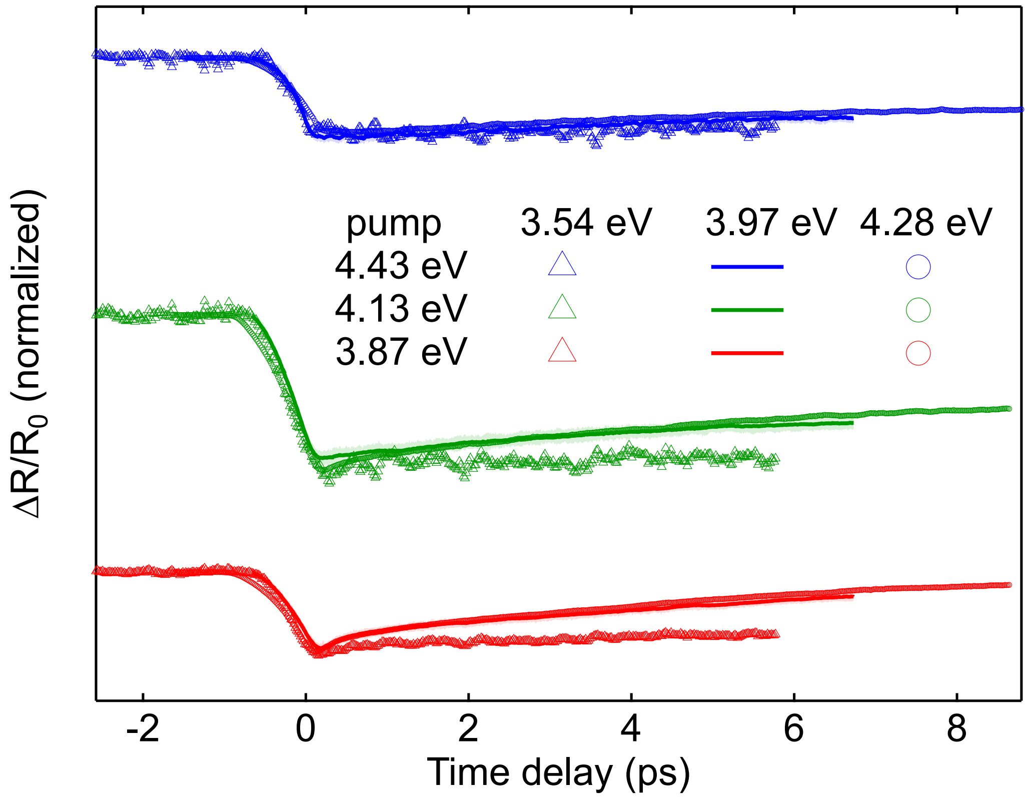

Fig. S28: **Effect of pump photon energy on transient reflectivity kinetics of NiO (001) single crystal.** Time traces with pump photon energy at $3.54\,$eV (triangles), $3.97\,$eV (continuous curves) and $4.28\,$eV (circles) pump photon energy. The probe photon energies are $3.87\,$eV (red), $4.13\,$eV (green) and $4.43\,$eV (blue). The excitation density is $\sim 2.5 \times 10^{20}\,\mathrm{cm}^{-3}$ (~ 0.018 eh/uc).

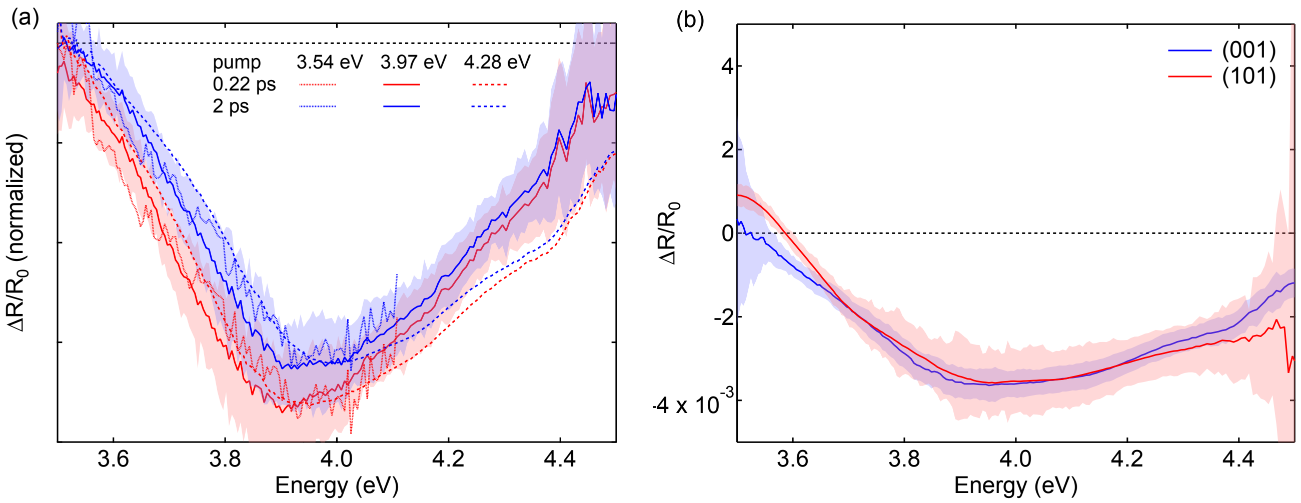

Fig. S29: **Effect of pump photon energy and surface orientation on transient reflectivity spectral traces of NiO single crystals.** (a) Normalized spectral traces at $0.22\,$ps (red curves) and $2\,$ps (blue curves) upon photodoping of NiO (001) single crystal at $3.54\,$eV (dotted curve), $3.97\,$eV (continuous curves), and $4.28\,$eV (dashed curves). The excitation density is $\sim 2.5 \times 10^{20}\,$cm^{-3} ($\sim 0.018$ eh/uc). (b) Effect of the NiO single crystal surface orientation on the spectral traces at $300\,$fs (pump $4.28\,$eV, excitation density $2.1 \times 10^{20}\,$cm^{-3}, 0.015 eh/uc). Shaded areas are standard deviations between individual measurements.

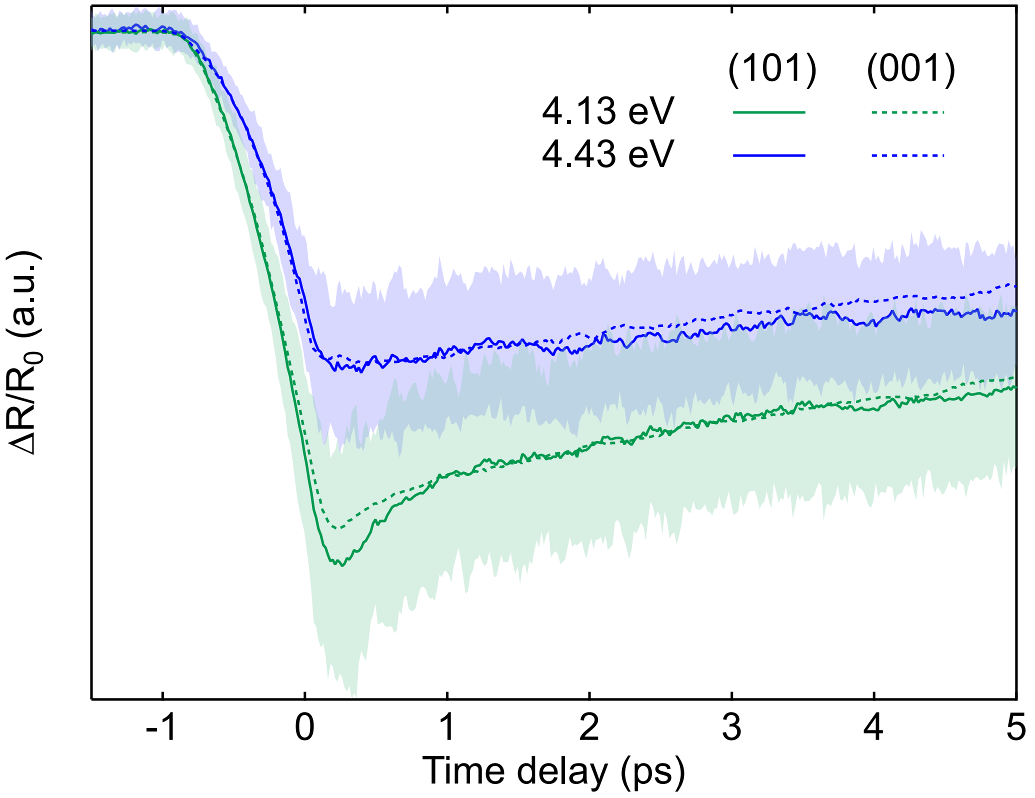

Fig. S30: **Effect of surface orientation on transient reflectivity kinetics.** Effect of the NiO single crystal surface orientation on the time traces at 4.13 eV (green curves) and 4.43 eV (blue curves) probe photon energies. Transient reflectivity are on NiO (101) (continuous curves) and NiO (001) (dashed curves). The pump photon energy is 4.28 eV and the excitation density is 2.1×10^{20} cm^{-3} (0.015 eh/uc). Shaded areas are standard deviations between individual measurements.

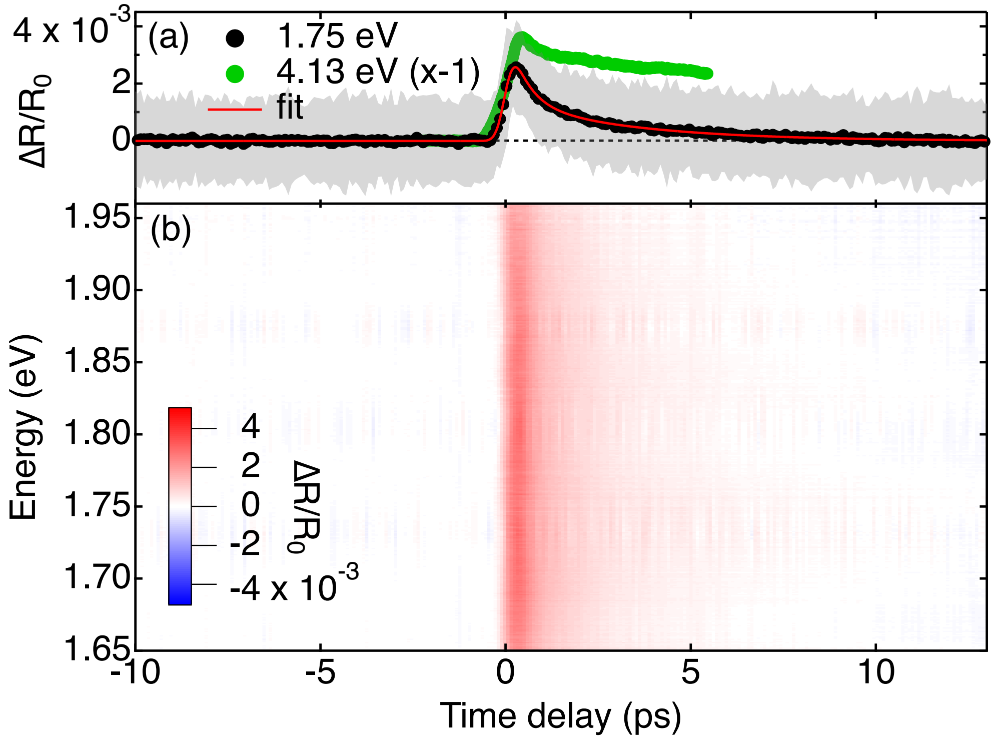

Fig. S31: **Transient reflectivity of NiO single crystal below the charge-transfer gap.** Transient reflectivity of NiO (101) single crystal under 4.51 eV pump excitation in the Visible (excitation density 1.58×10^{20} cm^{-3}). (a) Time trace at 1.75 eV (black circles) with a biexponential decay fitting (red curve, $\tau_1 = 0.5(1)$ ps, $\tau_2 = 3(1)$ ps) compared with a time trace at a probe photon energy of 4.13 eV (green circles). The black shaded area corresponds to the standard deviation between individual measurements. (b) Color-coded map of transient reflectivity.

8 Simulation spectral shift and spectral broadening

Figure S32 shows simulated relative differential transmission spectra upon spectral shifts (panel a) or spectral broadening (panel b) of the equilibrium spectrum by different amounts. A spectral shift displays a positive feature over most of the spectral window, incompatible with the wavelet lineshape in the experimental transient spectrum (see Figure S10b in the main text). A spectral broadening shows a wavelet lineshape with comparable positive and negative amplitudes, also incompatible with the experimental transient spectra displaying asymmetric positive and negative amplitudes (see Figure S10b in the main text).

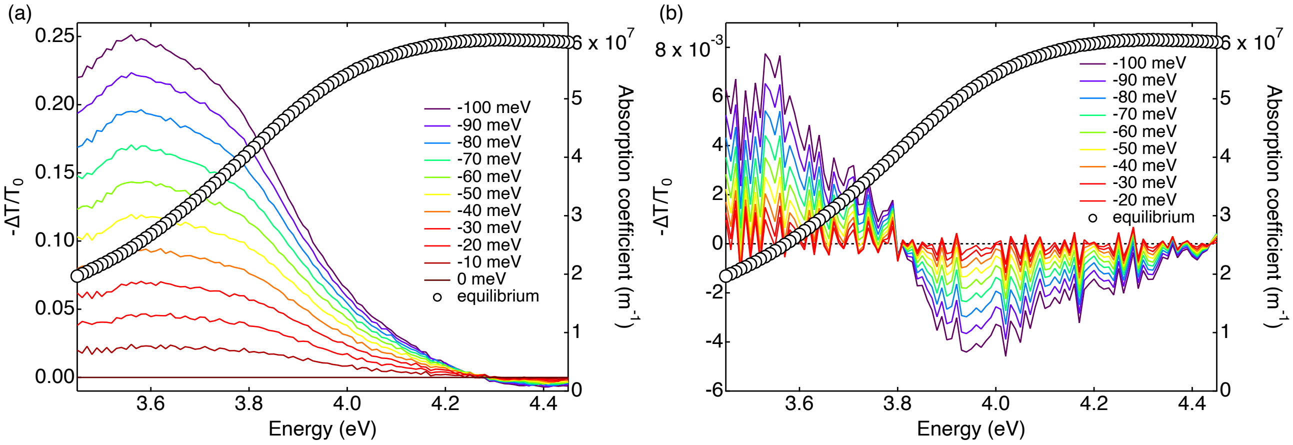

Fig. S32: **Simulated spectral energy shift and broadening on transient transmission.** Simulation of differential transmission upon (a) spectral energy shift or (b) spectral broadening of the equilibrium spectrum (colored curves, left axis). The equilibrium spectrum is shown with black circles for reference (right axis).

9 Spectral lineshape analysis

9.1 Modeling of the NiO optical properties

To extract physical parameters from the transient optical spectra of NiO upon photodoping, we perform a spectral lineshape analysis of the transient (in transmission and in reflectivity). First, the optical permittivity of the NiO thin film is modeled with a linear combination of oscillators to model the equilibrium (unpumped) optical properties of the film (detail in SI §1.3.2). A combination of two Tauc-Lorentz and one Lorentz oscillators are necessary to fit the permittivity between 0.9 and 4.8 eV (SI Figure S5). The fitted parameters are summarized in Table S5. The thickness of the thin film (17.6 nm) is provided by X-ray reflectivity measurements (see SI §1.4). The substrate is modeled as transparent MgO with a thickness of 500 µm. Second, the fitted parameters by spectroscopic ellipsometry are used to generate optical quantities such as the transmitted or reflected probe intensity from the NiO thin film. This requires computing the Fresnel equations for a dielectric slab, which is performed with the PyLlama package (*94*). The NiO substrate roughness is neglected as well as any intermediate layer of mixed composition between the film and the substrate. The input and output media of the light across the film and substrate are assumed to be air with a constant real refractive index of 1.0. Third, a fitting of the transient transmission or reflectivity is performed using the same optical model for the NiO excited (pumped) state as in the ground (unpumped) state, with the difference that the parameters of some oscillators in the excited state are left free to vary to model the sample response to the excitation. Since the transient measurements cover the spectral range between ~ 3.45 eV and ~ 4.3 eV, only the parameters of the *Tauc Lorentz 1* and *Lorentz* oscillators (see Table S5) are left free to vary since they have resonances in the spectral window of the probe. A parameter constrain is that the energy difference between E_0 and E_g is kept constant since we aim at modeling a global energy shift of *Tauc Lorentz 1* oscillator. The optical dielectric constant $\epsilon_{1\infty}$ is also kept constant. The fitting is performed with the `lmfit` python package (*95*) with the Levenberg-Marquardt algorithm. The spectral traces are weighted by the inverse of the standard deviation between individual measurements for greater statistical significance of the fitting. The fitting aims at minimizing the difference between the computed transient transmission or reflectivity with the Fresnel equations and the experimental one.

Since some oscillators are defined from their refractive index and others from their permittivity, conversions between these two quantities are often required. The complex refractive index is related to

the complex permittivity with the relations,

$$n = \sqrt{\frac{\sqrt{\epsilon_1^2 + \epsilon_2^2}}{2} + \frac{\epsilon_1}{2}} \tag{S7}$$

$$k = \sqrt{\frac{\sqrt{\epsilon_1^2 + \epsilon_2^2}}{2} - \frac{\epsilon_1}{2}}. \tag{S8}$$

Conversely, the complex permittivity is related to the complex refractive index with the relations,

$$\epsilon_1 = n^2 - k^2 \tag{S9}$$

$$\epsilon_2 = 2nk. \tag{S10}$$

9.2 Fitting results

Fitted spectral traces for the fluence dependence at 2 ps at room temperature in transmission and in reflectivity are displayed in Figure S33 and S35, respectively.

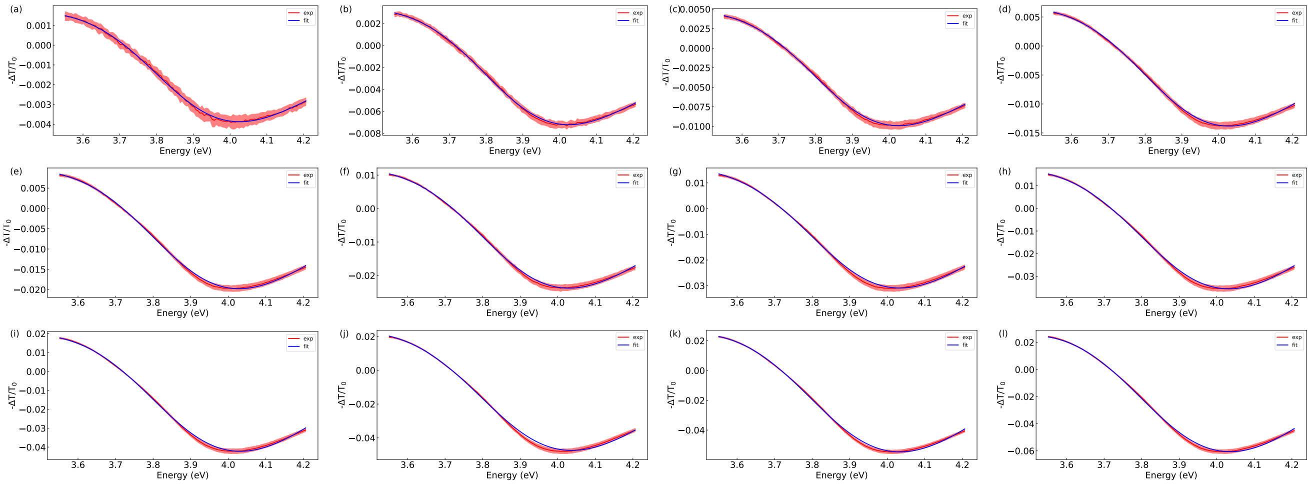

Fig. S33: **Fitting of spectral traces in transmission with phenomenological oscillator model.** Results of the fitting of the spectral traces at 2 ps ps in transmission (fluence dependence) for excitation densities: (a) 6.72×10^{18} cm^{-3} (0.0005 eh/uc), (b) 1.34×10^{19} cm^{-3} (0.001 eh/uc), (c) 2.01×10^{19} cm^{-3} (0.0015 eh/uc), (d) 2.69×10^{19} cm^{-3} (0.0019 eh/uc), (e) 4.03×10^{19} cm^{-3} (0.0029 eh/uc), (f) 5.37×10^{19} cm^{-3} (0.0039 eh/uc), (g) 6.72×10^{19} cm^{-3} (0.0049 eh/uc), (h) 8.40×10^{19} cm^{-3} (0.0061 eh/uc), (i) 1.01×10^{20} cm^{-3} (0.0073 eh/uc), (j) 1.21×10^{20} cm^{-3} (0.0088 eh/uc), (k) 1.44×10^{20} cm^{-3} (0.010 eh/uc), and (l) 1.68×10^{20} cm^{-3} (0.012 eh/uc). Experimental transients are in red, fittings are in blue. Shaded areas represent standard deviations between individual measurements.

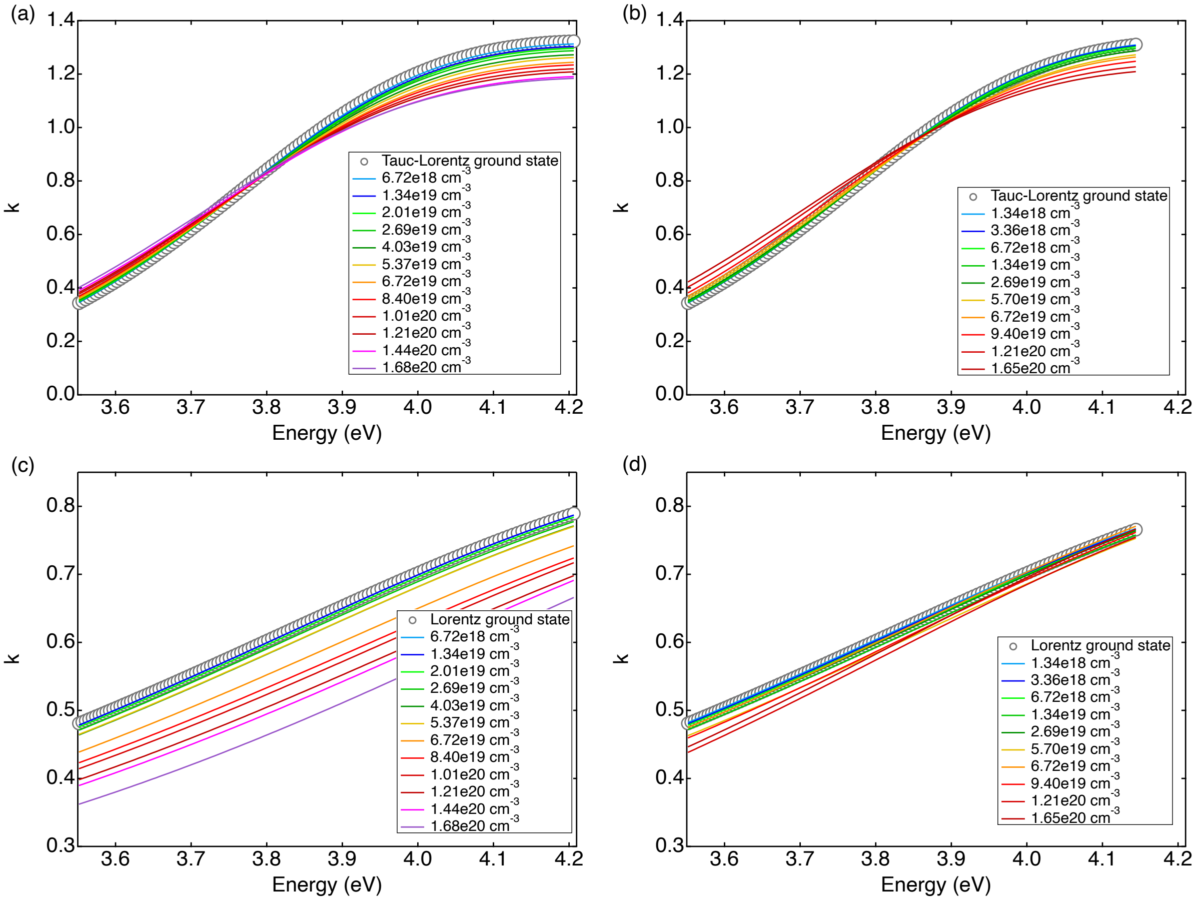

Fig. S34: **Fitted imaginary part of refractive index with phenomenological oscillator model.** Evolution of the fitted imaginary part of the refractive index (k) of the excited state (pumped) (a,b) Tauc-Lorentz oscillator at the charge-transfer gap and (c,d) Lorentz oscillator above the gap with increasing excitation densities (colored curves) at 2 ps for the measurement in (a,c) transmission, and (b,d) reflectivity. The imaginary part of the refractive index of the ground state (unpumped) Tauc-Lorentz oscillator is shown with grey circles for reference.

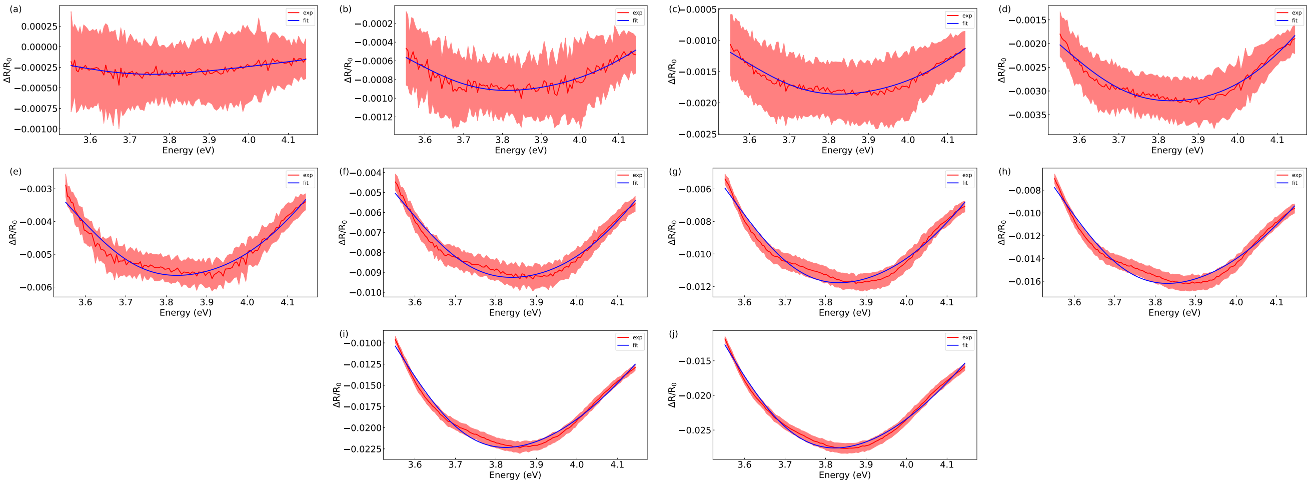

Fig. S35: **Fitted of spectral traces in reflectivity with phenomenological oscillator model.** Results of the fitting of the spectral traces at $2\,\mathrm{ps}$ in reflectivity (fluence dependence) for excitation densities: (a) 1.34×10^{18} cm^{-3} (<0.0001 eh/uc), (b) 3.36×10^{18} cm^{-3} (0.0002 eh/uc), (c) 6.72×10^{18} cm^{-3} (0.0005 eh/uc), (d) 1.34×10^{19} cm^{-3} (0.0010 eh/uc), (e) 2.69×10^{19} cm^{-3} (0.0019 eh/uc), (f) 4.70×10^{19} cm^{-3} (0.0034 eh/uc), (g) 6.72×10^{19} cm^{-3} (0.0049 eh/uc), (h) 9.40×10^{19} cm^{-3} (0.0068 eh/uc), (i) 1.21×10^{20} cm^{-3} (0.0088 eh/uc), and (j) 1.65×10^{20} cm^{-3} (0.0120 eh/uc). Experimental transients are in red, fittings are in blue. Shaded areas represent standard deviations between individual measurements.

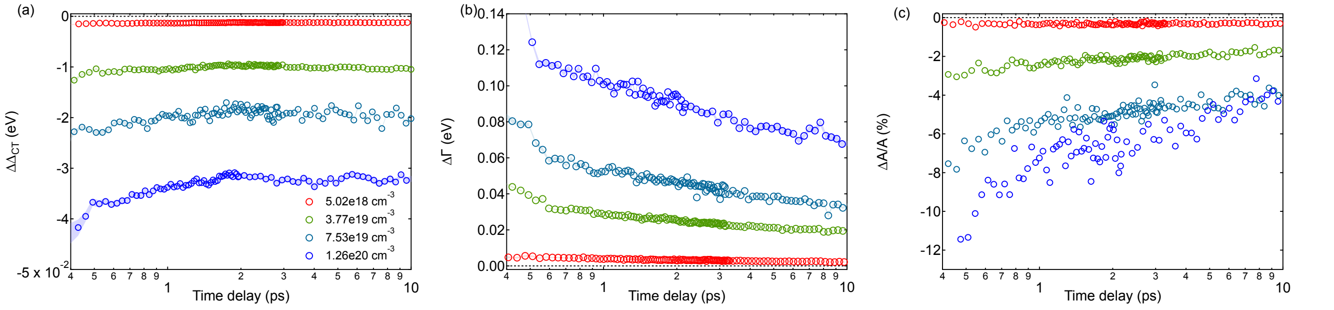

Fig. S36: **Evolution of oscillator parameters with time.** Fitted temporal evolution of the change in (a) CT gap (ΔE_g), (b) broadening ($\Delta\Gamma$), and (c) relative oscillator strength ($\Delta A/A$) of the Tauc-Lorentz oscillator at the CT gap upon excitation of NiO thin film at $4.66\,\mathrm{eV}$ in transmission. The excitation density is 5.02×10^{18} cm^{-3} (0.0004 eh/uc, red circles), 3.77×10^{19} cm^{-3} (0.0027 eh/uc, green circles), 7.53×10^{19} cm^{-3} (0.0055 eh/uc, light blue circles), and 1.26×10^{20} cm^{-3} (0.0091 eh/uc, dark blue circles).

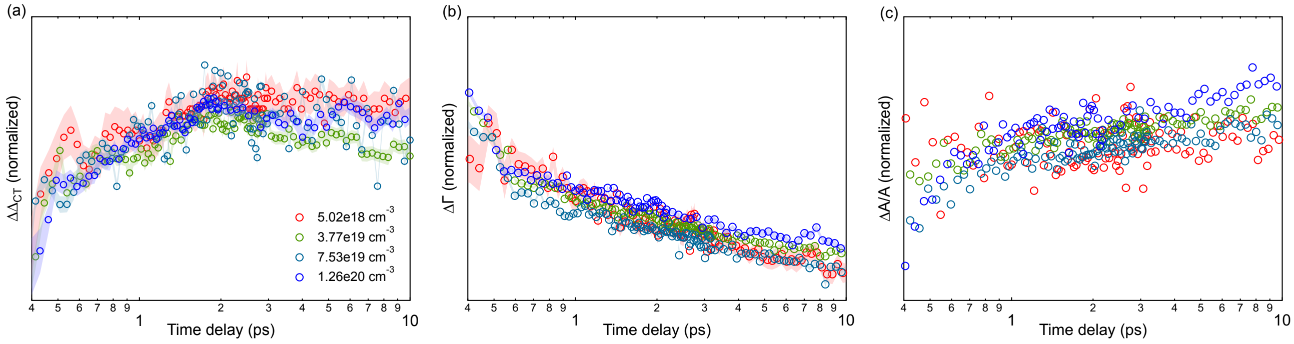

Fig. S37: **Evolution of normalized oscillator parameters with time.** Normalized fitted temporal evolution of the change in (a) CT gap (ΔE_g), (b) broadening ($\Delta\Gamma$), and (c) relative oscillator strength ($\Delta A/A$) of the Tauc-Lorentz oscillator at the CT gap upon excitation of NiO thin film at 4.66 eV in transmission. The excitation density is 5.02×10^{18} cm^{-3} (0.0004 eh/uc, red circles), 3.77×10^{19} cm^{-3} (0.0027 eh/uc, green circles), 7.53×10^{19} cm^{-3} (0.0055 eh/uc, light blue circles), and 1.26×10^{20} cm^{-3} (0.0091 eh/uc, dark blue circles).

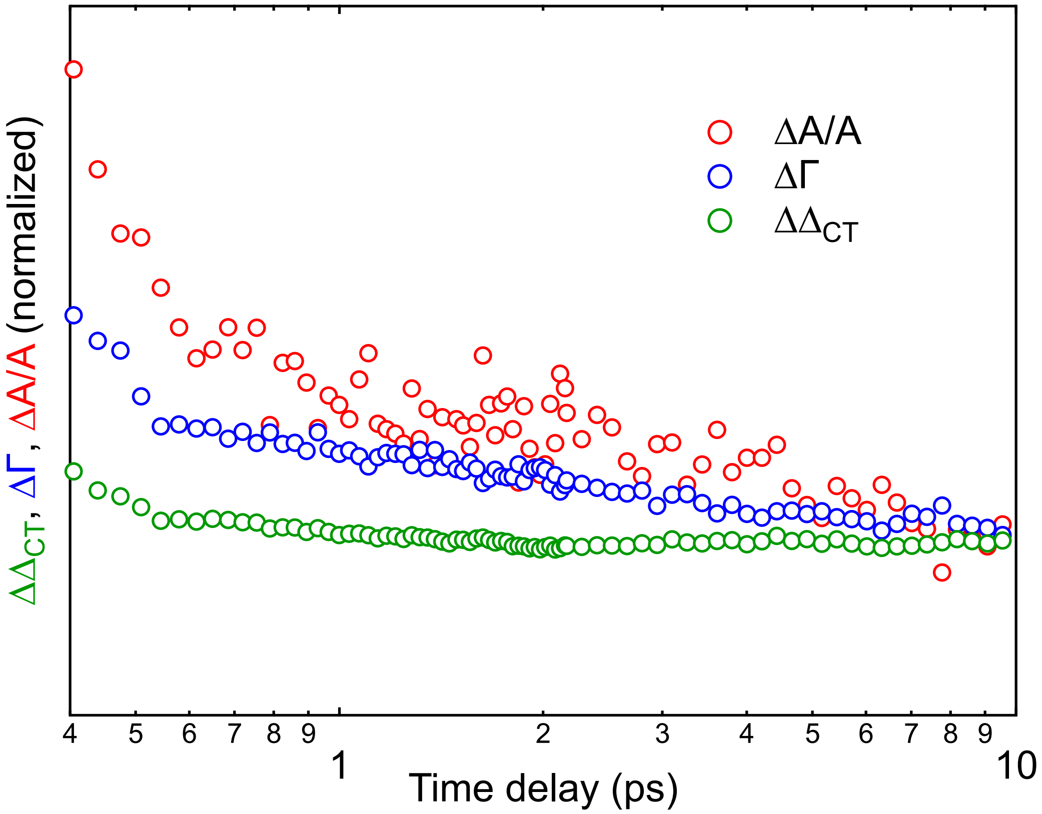

Fig. S38: **Evolution of normalized oscillator parameters with time at a fixed excitation density.** Temporal evolution of the normalized parameters of the lineshape analysis of the transient. Pump energy is 4.66 eV, excitation density is 1.26×10^{20} cm^{-3} (0.0091 eh/uc).

10 Comparison with the results of Lojewski et al.

The results on the Δ_{CT} renormalization and broadening change at the CT gap can be compared with the recent femtosecond XAS study at the Ni $L_{2,3}$- and O K-edge upon pumping above the CT gap of a NiO thin film (*10*). In the present work, Δ_{CT} renormalization reaches ~ 50 meV at an excitation density of $\sim 1.8 \times 10^{20}$ cm^{-3} (~ 0.013 eh/uc), which is larger than the $\sim 10 - 20$ meV and $\sim 30 - 40$ meV red shifts observed by femtosecond XAS at the Ni $L_{2,3}$- and O K-edge, respectively (excitation density $\sim 0.01 - 0.03$ eh/uc). However, the red shift observed by transient optical spectroscopy is sensitive to the sum of the self-energy renormalizations in the p- and d-bands at the CT gap, whereas femtosecond XAS at the Ni $L_{2,3}$- and O K-edge is separately sensitive to the self-energy renormalization in the d- and p-bands, respectively, a consequence of the selection rules for dipole transitions. This difference can explain the larger red shift here observed since the optical density is proportional to the joint density of states of the p- and d-bands, both of which undergo self-energy renormalization upon photodoping (*15*). In fact, Δ_{CT} renormalization is $40(2)$ meV at 0.01 eh/uc, which is in excellent agreement with the value of $40(5)$ meV reported in ref. (*10*) at the same photodoping level by summing up the energy renormalization at the Ni $L_{2,3}$- and O K-edge, assuming the occupied O $2p$ band at the top of the valence band shifts by the same amount as the unoccupied one at the bottom of the conduction band. The broadening at the CT gap ($\Delta\Gamma$) finds its origin in the dynamic screening induced by nonlocal charge fluctuations (*15*), which reaches a maximum of ~ 120 meV at the maximum excitation density (~ 0.013 eh/uc). Femtosecond XAS reveals different broadenings of ~ 120 and ~ 50 meV at the O K- and Ni $L_{2,3}$-edge, respectively, at a comparable excitation density. The larger broadening at the O K-edge is assigned to the itinerant p-electrons, which leads to a larger lifetime broadening (*96*). We expect the broadening in this work ($\Delta\Gamma$) to be a convolution of the broadening of the spectral density at the top of the valence band ($\Delta\Gamma_p$) and the bottom of the UHB ($\Delta\Gamma_d$) such that $\Delta\Gamma \sim \sqrt{\Delta\Gamma_p^2 + \Delta\Gamma_d^2}$. In this approximation, the total broadening from femtosecond XAS is ~ 130 meV, in excellent agreement with the broadening observed in the present work (~ 120 meV).

11 TDDFT simulations

All the calculations presented here were performed for bulk NiO, which is a type-II antiferromagnetic material below its Néel temperature ($T_N = 523$ K (*50*)). We neglected the small rhombohedral distortions and considered NiO in its cubic rock-salt structure, which does not affect the result of calculated

optical spectra. Calculations were performed neglecting spin-orbit coupling using fully norm-conserving pseudo-potentials. We employed a lattice parameter of $4.1704\,\text{Å}$, a real-space spacing of $\Delta r = 0.31$ Bohr, and a $16 \times 16 \times 8$ $\mathbf{k}$-point grid to sample the Brillouin zone. The driving field is taken along the [100] crystallographic direction in all the calculations. We consider a laser pulse of $55\,$fs duration (FWHM), with a sin-square envelope for the vector potential. The experimental carrier wavelength $\lambda = 266.06\,$nm was employed, corresponding to the experimental carrier photon energy of $4.66\,$eV. In all calculations, we set the carrier envelope phase (CEP) to zero. The time-dependent wavefunctions, number of excited electrons, and $U_{\rm eff}$ are computed by propagating generalized Kohn-Sham equations within real-time TDDFT+U, as provided by the Octopus code (*51*). We employed the LDA functional (*52*) for describing the semilocal DFT part, and we computed the effective $U_{\rm eff} = U - J$ for the O $2p$ ($U_{\rm eff}^{2p}$) and Ni $3d$ orbitals ($U_{\rm eff}^{3d}$), using localized atomic orbitals from the corresponding pseudopotentials (*35*). All calculations are propagated for $15\,$fs after the end of the pulse, to avoid spurious numerical effects.

The time-dependent generalized Kohn-Sham equation within the adiabatic approximation reads (note that the nonlocal part of the pseudopotential is omitted for conciseness)

$$i\frac{\partial}{\partial t}|\psi_{n,\mathbf{k}}(t)\rangle = \Big[\frac{(\hat{\mathbf{p}} - \mathbf{A}_{\rm ext}(t)/c)}{2} + \hat{v}_{\rm ext} + \hat{v}_{\rm H}[n(\mathbf{r},t)] + \hat{v}_{\rm xc}[n(\mathbf{r},t)] + \hat{V}_U[n(\mathbf{r},t),\{n_{mm'}\}]\Big]|\psi_{n,\mathbf{k}}(t)\rangle, \tag{S11}$$

where $|\psi_{n,\mathbf{k}}\rangle$ is a Bloch state with a band index n, at the point $\mathbf{k}$ in the Brillouin zone, $\hat{v}_{\rm ext}$ is the ionic potential, $\mathbf{A}_{\rm ext}(t)$ is the vector potential describing the laser field, $\hat{v}_{\rm H}$ is the Hartree potential, $\hat{v}_{\rm xc}$ is the exchange-correlation potential. $\hat{V}_U$ is the non-local operator for DFT+U that depends on also on the occupation matrix of the localized subspace $\{n_{mm'}\}$, see Ref. (*35*) for more details, including the definition of U, J, and $\hat{V}_U$.

The total number of excited electron is defined by projecting the time-evolved wavefunctions ($|\psi_n(t)\rangle$) on the basis of the ground-state wavefunctions ($|\psi_{n'}^{\rm GS}(t)\rangle$)

$$N_{\rm ex}(t) = N_e - \frac{1}{N_{\mathbf{k}}}\sum_{n,n'}^{\rm occ.}\sum_{\mathbf{k}}^{\rm BZ}|\langle\psi_{n,\mathbf{k}}(t)|\psi_{n',\mathbf{k}}^{\rm GS}\rangle|^2, \tag{S12}$$

where N_e is the total number of electrons in the system, and $N_{\mathbf{k}}$ is the total number of $\mathbf{k}$-points used to sample the BZ. The sum over the band indices n and n' run over all occupied states.

We also performed constrained DFT+U simulations. For this, we employed the constrained DFT method proposed in Ref. (*53*), in which we constrained the number of excited electrons from the

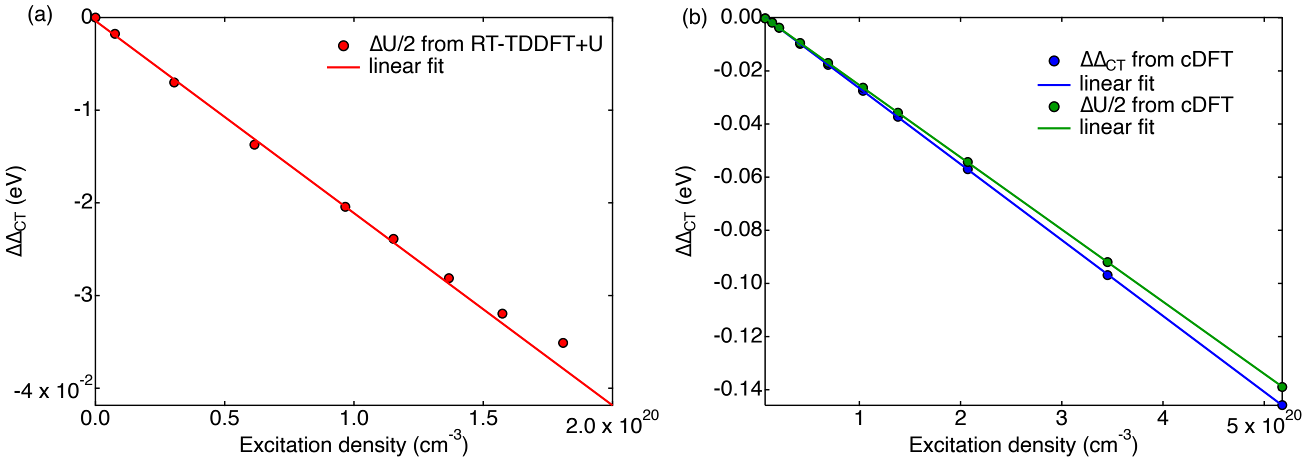

Fig. S39: **Charge-transfer gap renormalization calculated by RT-TDDFT+U and cDFT+U.** (a) Evolution of the CT gap renormalization ($\Delta\Delta_{\mathrm{CT}}$) as a function of the excitation density obtained form RTTDFT+U (red circles) and linear fit constrained to the data points at excitation densities lower than 1.2×10^{20} cm^{-3} to show the saturation of $\Delta\Delta_{\mathrm{CT}}$ with the excitation density (red curve). (b) Evolution of $\Delta\Delta_{\mathrm{CT}}$ as a function of the excitation density obtained by cDFT+U (blue circles). Comparison with the renormalization of electronic correlations ($\Delta U/2$, green circles). Linear fits are also shown.

experimental values and obtained the ground state and the self-consistent effective Hubbard U for each values of the number of excited electrons.

Figure S39a shows a saturation of the renormalization of Δ_{CT} with the excitation density in RT-TDDFT+U. Figure S39b shows the magnitude of the renormalization of Δ_{CT} calculated by cDFT+U matches half the renormalization of electronic correlations ($\Delta U/2$).

12 Pump-probe setup

The ultrafast broadband UV experiments have been performed with a setup providing narrowband UV pump and broadband UV probe pulses between 3.35 and 4.8 eV. The setup has been extensively described in references (*11, 12*). We hereby briefly summarize the main parameters of the setup. First, a 20 kHz Ti:Sapphire laser and cryo-cooled regenerative amplifier (KMLabs, Halcyon oscillator and Wyvern500 amplifier) provide 50 fs FWHM pulses at 1.55 eV with typically ~ 0.7 mJ per pulse (12 W average power). Around 7 W are used to pump a non-collinear optical parametric amplifier (NOPA, TOPAS White - Light Conversion) which provides < 100 fs pulses with a very broad spectral coverage

typically between 1.65 and $2.5\,\text{eV}$ with $\sim 7\,\mu\text{J}$ per pulse in this broadband configuration. Around $60\,\%$ of the NOPA output power is used to generate the narrowband pump pulses. In this optical line, the Visible pulse goes through a chopper operating at $10\,\text{kHz}$, synchronized to the laser system and phase-locked manually via the detection of the transmitted intensity with a photodiode. The pump pulse goes through an interference filter to select the fundamental of the pump photon energy and then through a phase-matched BBO to provide the UV pump pulse. Typical BBO thickness is less than $1\,\text{mm}$ to conserve the temporal width of the pump pulse. The typical bandwidth of the UV pump pulse is $20\,\text{meV}$ with a pulse energy of the order of $100\,\text{nJ}$. The pump pulse power density is recorded on a shot-to-shot basis by a calibrated photodiode for each pump photon energy, which allows for the normalization of the transient data based on the pump pulse energy. A half waveplate is used to set the relative polarization between the pump and the probe pulse at the magic angle ($54.74°$) to get rid of photoselection effects.

The remaining NOPA power is used to generate broadband UV probe pulses with $\sim 1.7\,\text{eV}$ bandwidth through an achromatic doubling scheme, which has been developed in the Riedle group (*49, 97*). It comprises two fused silica prisms that spatially disperse and recollimate the Visible beam coming from the NOPA. The resulting spatially chirped beam is focused with a $90°$ off-axis parabolic mirror on a $200\,\mu\text{m}$ thick BBO crystal. The frequency doubling of such broadband Visible pulse is complex because of a spectrally dependent phase-matching condition. Hence, the spatial chirp of the Visible beam and the different incident angles achieved by the parabola onto the BBO need to match the phase-matching at every probe photon energy simultaneously. The prisms also induce a temporal chirp required to avoid frequency mixing at the BBO. The frequency-doubled beam is subsequently recollimated with another $90°$ off-axis parabola, recombined and recompressed with two additional CaF_2 prisms.

The pump and probe pulses are focused onto the sample (the pump is at normal incidence and the probe at $\sim 7°$ in the refraction convention) where they are spatially and temporally overlapped. The beam waists are typically $\sim 80\,\mu\text{m}$ for the pump and $\sim 20\,\mu\text{m}$ for the probe, which results in a homogeneous probing of the excited sample volume. The reflected beam is steered, collimated and focused to couple into a multi-mode optical fiber ($100\,\mu\text{m}$), which is connected to the entrance slit of a $0.25\,\text{m}$ imaging spectrograph (Chromex 250is). The beam is dispersed by a 150 grooves/mm holographic grating and imaged onto a multichannel detector consisting of a 512 pixel CMOS linear sensor (Hamamatsu S11105, $12.5{\times}250\,\mu\text{m}$ pixel size) with up to $50\,\text{MHz}$ readout. The maximum readout rate per spectrum (almost $100\,\text{kHz}$) allows for easy shot-to-shot detection. The setup typically offers a time resolution of $\sim 150\,\text{fs}$, but it can be improved to $\sim 80\,\text{fs}$ with a set of chirp mirrors or a

prism compressor in the pump line.

The transient reflectivity is computed as,

$$\frac{\Delta R(\omega,t)}{R(\omega)} = \frac{R_{\text{pumped}}(\omega,t) - R_{\text{unpumped}}(\omega)}{R_{\text{unpumped}}(\omega)}. \tag{S13}$$

where $R(\omega,t)$ is the reflected intensity of the probe pulse from the sample surface at the optical frequency ω and time delay t between the pump and the probe. The transient transmission is computed as,

$$-\frac{\Delta T(\omega,t)}{T(\omega)} = -\frac{I_{\text{pumped}}(\omega,t) - I_{\text{unpumped}}(\omega)}{I_{\text{unpumped}}(\omega)}. \tag{S14}$$

where $I(\omega)$ is the transmitted intensity of the probe pulse through the sample at the optical frequency ω and time delay t.

The pump and probe beam waists are measured at low average powers with a Beamage-4M beam profiler from Gentec-EO without neutral density filter to reproduce the beam profile with high fidelity.

Every pump-probe measurements reported in this work have a pump photon energy exceeding the CT gap by ~ 0.7 eV at most, which means that impact ionization (generation of additional doublon-hole pairs by two-particle scattering processes) can be neglected.

Since a significant portion of the probe spectral range is above the CT gap of NiO, we checked the linearity of the transient signal with the probe pulse energy. Figure S40 shows that the kinetics upon photodoping of NiO thin film does not strongly depend on the probe power between 5 and 25 nJ. In this work, the probe pulse energy is fixed at 10 nJ.

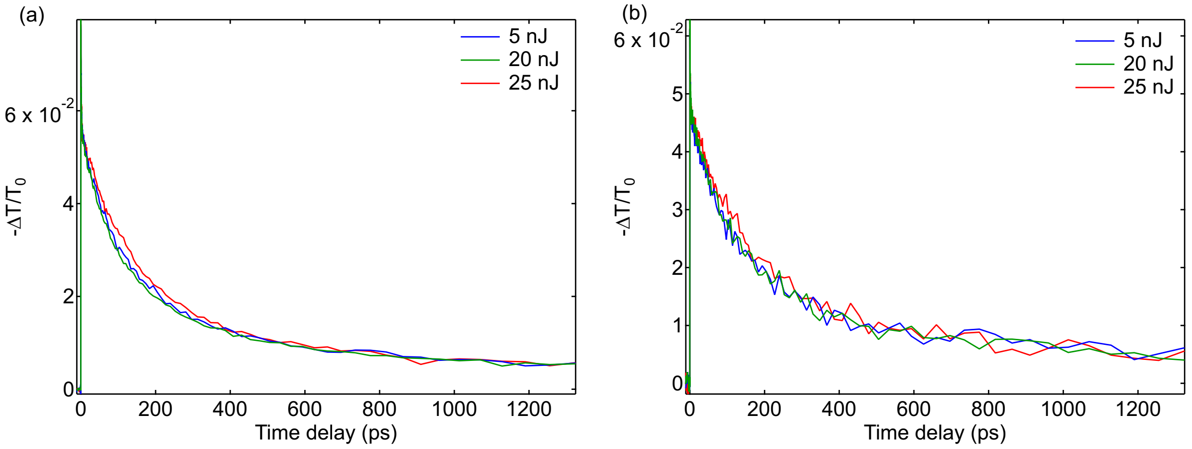

Fig. S40: **Probe pulse energy effect on kinetics.** Effect of the probe pulse energy on the time traces upon photodoping of NiO thin film at $4.28\,$eV (excitation density $1.26 \times 10^{20}\,$cm^{-3}, $0.009\,$eh/uc). The probe photon energy is (a) $3.8\,$eV, and (b) $4.2\,$eV.

REFERENCES AND NOTES

1. N. Tancogne-Dejean, M. A. Sentef, A. Rubio, Ultrafast modification of Hubbard *U* in a strongly correlated material: Ab initio high-harmonic generation in NiO. *Phys. Rev. Lett.* **121**, 097402 (2018).

2. L. Stojchevska, I. Vaskivskyi, T. Mertelj, P. Kusar, D. Svetin, S. Brazovskii, D. Mihailovic, Ultrafast switching to a stable hidden quantum state in an electronic crystal. *Science* **344**, 177–180 (2014).

3. J. Zhang, X. Tan, M. Liu, S. W. Teitelbaum, K. W. Post, F. Jin, K. A. Nelson, D. N. Basov, W. Wu, R. D. Averitt, Cooperative photoinduced metastable phase control in strained manganite films. *Nat. Mater.* **15**, 956–960 (2016).

4. M. Buzzi, D. Nicoletti, M. Fechner, N. Tancogne-Dejean, M. Sentef, A. Georges, T. Biesner, E. Uykur, M. Dressel, A. Henderson, T. Siegrist, J. Schlueter, K. Miyagawa, K. Kanoda, M.-S. Nam, A. Ardavan, J. Coulthard, J. Tindall, F. Schlawin, D. Jaksch, A. Cavalleri, Photomolecular high-temperature superconductivity. *Phys. Rev. X* **10**, 031028 (2020).

5. A. Verma, D. Golez, O. Y. Gorobtsov, K. Kaj, R. Russell, J. Z. Kaaret, E. Lamb, G. Khalsa, H. P. Nair, Y. Sun, R. Bouck, N. Schreiber, J. P. Ruf, V. Ramaprasad, Y. Kubota, T. Togashi, V. A. Stoica, H. Padmanabhan, J. W. Freeland, N. A. Benedek, O. G. Shpyrko, J. W. Harter, R. D. Averitt, D. G. Schlom, K. M. Shen, A. J. Millis, A. Singer, Picosecond volume expansion drives a later-time insulator–metal transition in a nano-textured Mott insulator. *Nat. Phys.* **20**, 807–814 (2024).

6. C. Giannetti, M. Capone, D. Fausti, M. Fabrizio, F. Parmigiani, D. Mihailovic, Ultrafast optical spectroscopy of strongly correlated materials and high-temperature superconductors: A non-equilibrium approach. *Adv. Phys.* **65**, 58–238 (2016).

7. A. Zong, B. R. Nebgen, S.-C. Lin, J. A. Spies, M. Zuerch, Emerging ultrafast techniques for studying quantum materials. *Nat. Rev. Mater.* **8**, 224–240 (2023).

8. N. Tancogne-Dejean, M. A. Sentef, A. Rubio, Ultrafast transient absorption spectroscopy of the charge-transfer insulator NiO: Beyond the dynamical Franz-Keldysh effect. *Phys. Rev. B* **102**, 115106 (2020).

9. D. R. Baykusheva, H. Jang, A. A. Husain, S. Lee, S. F. R. TenHuisen, P. Zhou, S. Park, H. Kim, J.-K. Kim, H.-D. Kim, M. Kim, S.-Y. Park, P. Abbamonte, B. J. Kim, G. D. Gu, Y. Wang, M. Mitrano, Ultrafast renormalization of the on-site coulomb repulsion in a cuprate superconductor. *Phys. Rev. X* **12**, 011013 (2022).

10. T. Lojewski, D. Golež, K. Ollefs, L. Le Guyader, L. Kämmerer, N. Rothenbach, R. Y. Engel, P. S. Miedema, M. Beye, G. S. Chiuzbăian, R. Carley, R. Gort, B. E. Van Kuiken, G. Mercurio, J. Schlappa, A. Yaroslavtsev, A. Scherz, F. Döring, C. David, H. Wende, U. Bovensiepen, M. Eckstein, P. Werner, A. Eschenlohr, Photoinduced charge transfer renormalization in NiO. *Phys. Rev. B* **110**, 245120 (2024).

11. G. Auböck, C. Consani, R. Monni, A. Cannizzo, F. van Mourik, M. Chergui, Femtosecond pump/supercontinuum-probe setup with 20 kHz repetition rate. *Rev. Sci. Instrum.* **83**, 093105 (2012).

12. G. Auböck, C. Consani, F. van Mourik, M. Chergui, Ultrabroadband femtosecond two-dimensional ultraviolet transient absorption. *Opt. Lett.* **37**, 2337 (2012).

13. Y. Gong, S. Zhang, H. Gao, Z. Ma, S. Hu, Z. Tan, Recent advances and comprehensive insights on nickel oxide in emerging optoelectronic devices. *Sustain. Energy Fuels* **4**, 4415–4458 (2020).

14. S. S. Mao, F. Quere, S. Guizard, X. Mao, R. E. Russo, G. Petite, P. Martin, Dynamics of femtosecond laser interactions with dielectrics. *Appl. Phys. A* **79**, 1695–1709 (2004).

15. D. Golež, L. Boehnke, M. Eckstein, P. Werner, Dynamics of photodoped charge transfer insulators. *Phys. Rev. B* **100**, 041111(R) (2019).

16. H. Okamoto, T. Miyagoe, K. Kobayashi, H. Uemura, H. Nishioka, H. Matsizaki, A. Sawa, Y. Tokura, Ultrafast charge dynamics in photoexcited Nd_2CuO_4 and La_2CuO_4 cuprate

compounds investigated by femtosecond absorption spectroscopy. *Phys. Rev. B* **82**, 060513–060514 (2010).

17. H. Okamoto, T. Miyagoe, K. Kobayashi, H. Uemura, H. Nishioka, H. Matsuzaki, A. Sawa, Y. Tokura, Photoinduced transition from Mott insulator to metal in the undoped cuprates Nd_2CuO_4 and La_2CuO_4. *Phys. Rev. B* **83**, 125102–125110 (2011).

18. F. Novelli, G. De Filippis, V. Cataudella, M. Esposito, I. Vergara, F. Cilento, E. Sindici, A. Amaricci, C. Giannetti, D. Prabhakaran, S. Wall, A. Perucchi, S. D. Conte, G. Cerullo, M. Capone, A. Mishchenko, M. Grüninger, N. Nagaosa, F. Parmigiani, D. Fausti, Witnessing the formation and relaxation of dressed quasi-particles in a strongly correlated electron system. *Nat. Commun.* **5**, 5112 (2014).

19. Y. Windsor, D. Zahn, R. Kamrla, J. Feldl, H. Seiler, C.-T. Chiang, M. Ramsteiner, W. Widdra, R. Ernstorfer, L. Rettig, Exchange-striction driven ultrafast nonthermal lattice dynamics in NiO. *Phys. Rev. Lett.* **126**, 147202 (2021).

20. S. Biswas, J. Husek, S. Londo, L. R. Baker, Ultrafast electron trapping and defect-mediated recombination in NiO probed by femtosecond extreme ultraviolet reflection–absorption spectroscopy. *J. Phys. Chem. Lett.* **9**, 5047–5054 (2018).

21. G. Merzoni, L. Martinelli, S. Parchenko, S. F. R. TenHuisen, V. Lebedev, L. Adriano, R. Carley, N. Gerasimova, L. Mercadier, M. Teichmann, B. E. van Kuiken, Z. Yin, A. Alic, D. R. Baykusheva, S. G. Chiuzbaian, S. D. Conte, O. Dogadov, A. Föhlisch, M. W. Haverkort, M. Kusch, T. Laarmann, W. S. Lee, M. M. Sala, Y. Peng, Q. Z. Qiu, T. Schmitt, S. S. N. Lalithambika, S. Techert, G. Cerullo, M. Först, M. Mitrano, M. P. M. Dean, J. Schlappa, A. Scherz, G. Ghiringhelli, Photo-generated charge-transfer excitons in NiO revealed by ultrafast time-resolved resonant inelastic x-ray scattering. arXiv:2504.16653 [cond-mat.str-el] (2025).

22. S. Biswas, J. Husek, S. Londo, L. R. Baker, Highly localized charge transfer excitons in metal oxide semiconductors. *Nano Lett.* **18**, 1228–1233 (2018).

23. W. Lee, Y. D. Chuang, R. G. Moore, Y. Zhu, L. Patthey, M. Trigo, D. H. Lu, P. S. Kirchmann, O. Krupin, M. Yi, M. Langner, N. Huse, J. S. Robinson, Y. Chen, S. Y. Zhou, G. Coslovich, B. Huber, D. A. Reis, R. A. Kaindl, R. W. Schoenlein, D. Doering, P. Denes, W. F. Schlotter, J. J. Turner, S. L. Johnson, M. Först, T. Sasagawa, Y. F. Kung, A. P. Sorini, A. F. Kemper, B. Moritz, T. P. Devereaux, D.-H. Lee, Z. X. Shen, Phase fluctuations and the absence of topological defects in a photo-excited charge-ordered nickelate. *Nat. Commun.* **3**, 838 (2012).

24. X. Hu, P. Jiang, C. Ding, H. Yang, Q. Gong, Picosecond and low-power all-optical switching based on an organic photonic-bandgap microcavity. *Nat. Photonics* **2**, 185–189 (2008).

25. M. Ren, B. Jia, J. Y. Ou, E. Plum, J. Zhang, K. MacDonald, A. E. Nikolaenko, J. Xu, M. Gu, N. I. Zheludev, Nanostructured plasmonic medium for terahertz bandwidth all-optical switching. *Adv. Mater.* **23**, 5540–5544 (2011).

26. S. Bhakta, R. Panda, P. K. Sahoo, Third-order non-linear optical switching and threshold limiting of NiO thin films. *Sci. Rep.* **14**, 22767 (2024).

27. H. Ahmad, S. A. Reduan, N. Yusoff, Chitosan capped nickel oxide nanoparticles as a saturable absorber in a tunable passively Q-switched erbium doped fiber laser. *RSC Adv.* **8**, 25592–25601 (2018).

28. T. Palmieri, E. Baldini, A. Steinhoff, A. Akrap, M. Kollár, E. Horváth, L. Forró, F. Jahnke, M. Chergui, Mahan excitons in room-temperature methylammonium lead bromide perovskites. *Nat. Commun.* **11**, 850 (2020).

29. E. Baldini, M. A. Sentef, S. Acharya, T. Brumme, E. Sheveleva, F. Lyzwa, E. Pomjakushina, C. Bernhard, M. van Schilfgaarde, F. Carbone, A. Rubio, C. Weber, Electron–phonon-driven three-dimensional metallicity in an insulating cuprate. *Proc. Natl. Acad. Sci. U.S.A.* **117**, 6409–6416 (2020).

30. D. Golež, M. Eckstein, P. Werner, Multiband nonequilibrium *GW* + EDMFT formalism for correlated insulators. *Phys. Rev. B* **100**, 235117 (2019).

31. S. Richter, O. Herrfurth, S. Espinoza, M. Rebarz, M. Kloz, J. A. Leveillee, A. Schleife, S. Zollner, M. Grundmann, J. Andreasson, R. Schmidt-Grund, Ultrafast dynamics of hot charge carriers in an oxide semiconductor probed by femtosecond spectroscopic ellipsometry. *New J. Phys.* **22**, 083066 (2020).

32. O. Grånäs, I. Vaskivskyi, X. Wang, P. Thunström, S. Ghimire, R. Knut, J. Söderström, L. Kjellsson, D. Turenne, R. Y. Engel, M. Beye, J. Lu, D. J. Higley, A. H. Reid, W. Schlotter, G. Coslovich, M. Hoffmann, G. Kolesov, C. Schüßler-Langeheine, A. Styervoyedov, N. Tancogne-Dejean, M. A. Sentef, D. A. Reis, A. Rubio, S. S. P. Parkin, O. Karis, J.-E. Rubensson, O. Eriksson, H. A. Dürr, Ultrafast modification of the electronic structure of a correlated insulator. *Phy. Rev. Res.* **4**, L032030 (2022).

33. N. Tancogne-Dejean, A. Rubio, Parameter-free hybridlike functional based on an extended Hubbard model. *Phys. Rev. B* **102**, 155117 (2020).

34. N. Tancogne-Dejean, M. J. T. Oliveira, A. Rubio, Self-consistent DFT + U method for real-space time-dependent density functional theory calculations. *Phys. Rev. B* **96**, 245133 (2017).

35. L. A. Agapito, S. Curtarolo, M. Buongiorno Nardelli, Reformulation of DFT + U as a pseudohybrid Hubbard density functional for accelerated materials discovery. *Phys. Rev. X* **5**, 0110006 (2015).

36. P. Werner, A. J. Millis, Dynamical screening in correlated electron materials. *Phys. Rev. Lett.* **104**, 146401 (2010).

37. A. Chernikov, C. Ruppert, H. M. Hill, A. F. Rigosi, T. F. Heinz, Population inversion and giant bandgap renormalization in atomically thin WS_2 layers. *Nat. Photonics* **9**, 466–470 (2015).

38. L. Tian, L. di Mario, V. Zannier, D. Catone, S. Colonna, P. O'Keeffe, S. Turchini, N. Zema, S. Rubini, F. Martelli, Ultrafast carrier dynamics, band-gap renormalization, and optical properties of ZnSe nanowires. *Phys. Rev. B* **94**, 165442 (2016).

39. E. A. A. Pogna, M. Marsili, D. de Fazio, S. Dal Conte, C. Manzoni, D. Sangalli, D. Yoon, A. Lombardo, A. C. Ferrari, A. Marini, G. Cerullo, D. Prezzi, Photo-induced bandgap renormalization governs the ultrafast response of single-layer MoS_2. *ACS Nano* **10**, 1182–1188 (2016).

40. F. Liu, M. E. Ziffer, K. R. Hansen, J. Wang, X. Zhu, Direct determination of band-gap renormalization in the photoexcited monolayer MoS_2. *Phys. Rev. Lett.* **122**, 246803 (2019).

41. Y. Lin, Y.-h. Chan, W. Lee, L.-S. Lu, Z. Li, W.-H. Chang, C.-K. Shih, R. A. Kaindl, S. G. Louie, A. Lanzara, Exciton-driven renormalization of quasiparticle band structure in monolayer MoS_2. *Phys. Rev. B* **106**, L081117 (2022).

42. H. Y. Fan, Temperature dependence of the energy gap in semiconductors. *Phys. Rev.* **82**, 900–905 (1951).

43. D. Wegkamp, M. Herzog, L. Xian, M. Gatti, P. Cudazzo, C. McGahan, R. E. Marvel, R. F. Haglund Jr., A. Rubio, M. Wolf, J. Stähler, Instantaneous band gap collapse in photoexcited monoclinic VO_2 due to photocarrier doping. *Phys. Rev. Lett.* **113**, 216401 (2014).

44. T. Miyamoto, Y. Matsui, T. Terashige, T. Morimoto, N. Sono, H. Yada, S. Ishihara, Y. Watanabe, S. Adachi, T. Ito, K. Oka, A. Sawa, H. Okamoto, Probing ultrafast spin-relaxation and precession dynamics in a cuprate Mott insulator with seven-femtosecond optical pulses. *Nat. Commun.* **9**, 3948 (2018).

45. P. Werner, N. Tsuji, M. Eckstein, Nonthermal symmetry-broken states in the strongly interacting Hubbard model. *Phys. Rev. B* **86**, 205101 (2012).

46. N. Tsuji, M. Eckstein, P. Werner, Nonthermal antiferromagnetic order and nonequilibrium criticality in the Hubbard model. *Phys. Rev. Lett.* **110**, 136404 (2013).

47. X. Wang, R. Y. Engel, I. Vaskivskyi, D. Turenne, V. Shokeen, A. Yaroslavtsev, O. Grånäs, R. Knut, J. O. Schunck, S. Dziarzhytski, G. Brenner, R. P. Wang, M. Kuhlmann, F. Kuschewski, W. Bronsch, C. Schüßler-Langeheine, A. Styervoyedov, S. S. P. Parkin, F. Parmigiani, O.

Eriksson, M. Beye, H. A. Dürr, Ultrafast manipulation of the NiO antiferromagnetic order via sub-gap optical excitation. *Faraday Discuss.* **237**, 300–316 (2022).

48. P. Baum, S. Lochbrunner, E. Riedle, Generation of tunable 7-fs ultraviolet pulses: Achromatic phase matching and chirp management. *Appl. Phys. B* **79**, 1027–1032 (2004).

49. A. P. Cracknell, S. J. Joshua, The space group corepresentations of antiferromagnetic NiO. *Math. Proc. Camb. Philos. Soc.* **66**, 493–504 (1969).

50. N. Tancogne-Dejean, M. J. T. Oliveira, X. Andrade, H. Appel, C. H. Borca, G. Le Breton, F. Buchholz, A. Castro, S. Corni, A. A. Correa, U. De Giovannini, A. Delgado, F. G. Eich, J. Flick, G. Gil, A. Gomez, N. Helbig, H. Hübener, R. Jestädt, J. Jornet-Somoza, A. H. Larsen, I. V. Lebedeva, M. Lüders, M. A. L. Marques, S. T. Ohlmann, S. Pipolo, M. Rampp, C. A. Rozzi, D. A. Strubbe, S. A. Sato, C. Schäfer, I. Theophilou, A. Welden, A. Rubio, Octopus, a computational framework for exploring light-driven phenomena and quantum dynamics in extended and finite systems. *J. Chem. Phys.* **152**, 124119 (2020).

51. J. P. Perdew, A. Zunger, Self-interaction correction to density-functional approximations for many-electron systems. *Phys. Rev. B* **23**, 5048–5079 (1981).

52. G. Marini, M. Calandra, Lattice dynamics of photoexcited insulators from constrained density-functional perturbation theory. *Phys. Rev. B* **104**, 144103 (2021).

53. A. Ghosh, C. M. Nelson, L. S. Abdallah, S. Zollner, Optical constants and band structure of trigonal NiO. *J. Vac. Sci. Technol. A* **33**, 061203 (2015).

54. G. Remond, R. Myklebust, M. Fialin, C. Nockolds, M. Phillips, C. Roques-Carmes, Decomposition of wavelength dispersive X-ray spectra. *J. Res. Natl. Inst. Stand. Technol.* **107**, 509–529 (2002).

55. A. B. Kuzmenko, Kramers–Kronig constrained variational analysis of optical spectra. *Rev. Sci. Instrum.* **76**, 083108 (2005).

56. T. Dong, H. Suk, H. Hosun, Optical properties of black NiO and CoO single crystals studied with spectroscopic ellipsometry. *J. Korean Phys. Soc.* **50**, 632–637 (2007).

57. J.-L. Li, G.-M. Rignanese, S. G. Louie, Quasiparticle energy bands of NiO in the gw approximation. *Phys. Rev. B* **71**, 193102 (2005).

58. R. Newman, R. M. Chrenko, Optical properties of nickel oxide. *Phys. Rev.* **114**, 1507–1513 (1959).

59. R. Karsthof, Y. K. Frodason, A. Galeckas, P. M. Weiser, V. Zviagin, M. Grundmann, Light absorption and emission by defects in doped nickel oxide. *Adv. Photon. Res.* **3**, 2200138 (2022).

60. F. Wrobel, H. Park, C. Sohn, H.-W. Hsiao, J.-M. Zuo, H. Shin, H. N. Lee, P. Ganesh, A. Benali, P. R. C. Kent, O. Heinonen, A. Bhattacharya, Doped NiO: The mottness of a charge transfer insulator. *Phys. Rev. B* **101**, 195128 (2020).

61. G. E. Jellison, F. A. Modine, Parameterization of the optical functions of amorphous materials in the interband region. *Appl. Phys. Lett.* **69**, 371–373 (1996).

62. H. L. Lu, G. Scarel, M. Alia, M. Fanciulli, S.-J. Ding, D. W. Zhang, Spectroscopic ellipsometry study of thin NiO films grown on Si (100) by atomic layer deposition. *Appl. Phys. Lett.* **92**, 222907 (2008).

63. K. O. Egbo, C. P. Liu, C. E. Ekuma, K. M. Yu, Vacancy defects induced changes in the electronic and optical properties of NiO studied by spectroscopic ellipsometry and first-principles calculations. *J. Appl. Phys.* **128**, 135705 (2020).

64. R. Stephens, I. Malitson, Index of refraction of magnesium oxide. *J. Res. Natl. Bur. Stand.* **49**, 249–252 (1952).

65. J. Brentano, Focussing method of crystal powder analysis by X-rays. *Proc. Phys. Soc. Lond.* **37**, 184–193 (1924).

66. C. J. Ksanda, Comparison standards for the powder spectrum method; NiO and CdO. *Am. J. Sci.* **s5-22**, 131–138 (1931).

67. R. W. Cairns, E. Ott, X-ray studies of the system nickel—oxygen—water. I. Nickelous oxide and hydroxide. *J. Am. Chem. Soc.* **55**, 527–533 (1933).

68. L. Thomassen, An X-ray investigation of the system Cr_2O_3-NiO. *J. Am. Chem. Soc.* **62**, 1134–1136 (1940).

69. A. Leineweber, H. Jacobs, S. Hull, Ordering of nitrogen in nickel nitride Ni_3N determined by neutron diffraction. *Inorg. Chem.* **40**, 5818–5822 (2001).

70. H. P. Rooksby, A note on the structure of nickel oxide at subnormal and elevated temperatures. *Acta Crystallogr.* **1**, 226–226 (1948).

71. P. Salunkhe, M. A. A. V., D. Kekuda, Investigation on tailoring physical properties of nickel oxide thin films grown by dc magnetron sputtering. *Mater. Res. Express* **7**, 016427 (2020).

72. H. Lee, Y.-T. Huang, M. W. Horn, S.-P. Feng, Engineered optical and electrical performance of rf–sputtered undoped nickel oxide thin films for inverted perovskite solar cells. *Sci. Rep.* **8**, 5590 (2018).

73. S. Handley, G. Bradberry, Estimates of charge carrier mobility and lifetime in nickel oxide. *Phys. Lett. A* **40**, 277–278 (1972).

74. A. Singer, M. J. Marsh, S. H. Dietze, V. Uhlíř, Y. Li, D. A. Walko, E. M. Dufresne, G. Srajer, M. P. Cosgriff, P. G. Evans, E. E. Fullerton, O. G. Shpyrko, Condensation of collective charge ordering in chromium. *Phys. Rev. B* **91**, 115134 (2015).

75. R. J. Stoner, H. J. Maris, Kapitza conductance and heat flow between solids at temperatures from 50 to 300 K. *Phys. Rev. B* **48**, 16373–16387 (1993).

76. R. M. Costescu, M. A. Wall, D. G. Cahill, Thermal conductance of epitaxial interfaces. *Phys. Rev. B* **67**, 054302 (2003).

77. G. A. Slack, R. Newman, Thermal conductivity of MnO and NiO. *Phys. Rev. Lett.* **1**, 359–360 (1958).

78. V. Zhuze, O. Novruzov, A. Shelykh, Thermal conductivity near a continuous phase ttransition (experimental, with sodium nitrate, barium titanate, mangasnese telluride, nickel oxide, cobalt oxide). *Inst. Semicond. Leningrad. Sov. Phys. Solid State* **11**, 1044–1051 (1969).

79. V. Shchelkotunov, V. Danilov, Effect of magnetic state of ferrimagnetics and anti-ferromagnetics of basic structural type on mechanism of heat energy spreading. *Izv. Akad. Nauk SSSR Ser. Fiz.* **35**, 1158 (1971).

80. F. B. Lewis, N. H. Saunders, The thermal conductivity of NiO and CoO at the Neel temperature. *J. Phys. C Solid State Phys.* **6**, 2525–2532 (1973).

81. J. Linnera, A. J. Karttunen, Lattice dynamical properties of antiferromagnetic MnO, CoO, and NiO, and the lattice thermal conductivity of NiO. *Phys. Rev. B* **100**, 144307 (2019).

82. Q. Sun, S. Hou, B. Wei, Y. Su, V. Ortiz, B. Sun, J. Y. Y. Lin, H. L. Smith, S. Danilkin, D. L. Abernathy, R. Wilson, C. Li, Spin-phonon interactions induced anomalous thermal conductivity in nickel (II) oxide. *Mater. Today Phys.* **35**, 101094 (2023).

83. D. R. Lide, *CRC Handbook of Chemistry and Physics* (CRC Press, 2004), vol. 85.

84. H. Seltz, B. J. DeWitt, H. J. McDonald, The heat capacity of nickel oxide from 68-298°K. and the thermodynamic properties of the oxide. *J. Am. Chem. Soc.* **62**, 88–89 (1940).

85. J. J. Snellenburg, S. P. Laptenok, R. Seger, K. M. Mullen, I. H. M. v. Stokkum, Glotaran: A Java-based graphical user interface for the R package TIMP. *J. Stat. Softw.* **49**, 1–22 (2012).

86. I. H. M. van Stokkum, J. Weißenborn, S. Weigand, J. J. Snellenburg, Pyglotaran: A lego-like Python framework for global and target analysis of time-resolved spectra. *Photochem. Photobiol. Sci.* **22**, 2413–2431 (2023).

87. M. B. Price, J. Butkus, T. C. Jellicoe, A. Sadhanala, A. Briane, J. E. Halpert, K. Broch, J. M. Hodgkiss, R. H. Friend, F. Deschler, Hot-carrier cooling and photoinduced refractive index changes in organic–inorganic lead halide perovskites. *Nat. Commun.* **6**, 8420 (2015).

88. R. R. Tamming, R. R. Tamming, J. Butkus, M. B. Price, P. Vashishtha, S. K. K. Prasad, J. E. Halpert, K. Chen, J. M. Hodgkins, Ultrafast spectrally resolved photoinduced complex refractive index changes in $CsPbBr_3$ perovskites. *ACS Photonics* **6**, 345–350 (2019).

89. R. Glosser, W. Walker, L. Messick, Thermoreflectance of NiO. *Phys. Lett. A* **39**, 163–164 (1972).

90. L. Messick, W. C. Walker, R. Glosser, Direct and temperature-modulated reflectance spectra of MnO, CoO, and NiO. *Phys. Rev. B* **6**, 3941–3949 (1972).

91. L. Messick, W. Walker, R. Glosser, Direct and modulated reflectance spectra of MnO, CoO, and NiO. *Surf. Sci.* **37**, 267–279 (1973).

92. R. Glosser, W. Walker, Electroreflectance observation of localized and itinerant electron states in NiO. *Solid State Commun.* **9**, 1599–1602 (1971).

93. L. D'Amario, J. Föhlinger, G. Boschloo, L. Hammarström, Unveiling hole trapping and surface dynamics of NiO nanoparticles. *Chem. Sci.* **9**, 223–230 (2018).

94. M. M. Bay, S. Vignolini, K. Vynck, Pyllama: A stable and versatile Python toolkit for the electromagnetic modelling of multilayered anisotropic media. *Comput. Phys. Commun.* **273**, 108256 (2022).

95. M. Newville, R. Otten, A. Nelson, A. Ingargiola, T. Stensitzki, D. Allan, A. Fox, F. Carter, Michał, R. Osborn, D. Pustakhod, lneuhaus, S. Weigand, Glenn, C. Deil, Mark, A. L. R. Hansen, G. Pasquevich, L. Foks, N. Zobrist, O. Frost, A. Beelen, Stuermer, azelcer, A. Hannum, A. Polloreno, J. H. Nielsen, S. Caldwell, A. Almarza, A. Persaud, lmfit/lmfit-py: 1.0.3, Zenodo (2021); https://doi.org/10.5281/zenodo.5570790.

96. T. Lojewski, M. F. Elhanoty, L. L. Guyader, O. Granas, N. Agarwal, C. Boeglin, R. Carley, A. Castoldi, C. David, C. Deiter, F. Doring, R. Y. Engel, H. Fangohr, C. Fiorini, P. Fischer, N. Gerasimova, R. Gort, F. deGroot, K. Hansen, S. Hauf, D. Hickin, M. Izquierdo, B. E. Van Kuiken, Y. Kvashnin, C.-H. Lambert, D. Lomidze, S. Maffessanti, L. Mercadier, G. Mercurio, P. S. Miedema, K. Ollefs, M. Pace, M. Porro, J. Rezvani, B. Rosner, N. Rothenbach, A. Samartsev, A. Scherz, J. Schlappa, C. Stamm, M. Teichmann, P. Thunstrom, M. Turcato, A. Yaroslavtsev, J. Zhu, M. Beye, H. Wende, U. Bovensiepen, O. Eriksson, A. Eschenlohr, The interplay of local electron correlations and ultrafast spin dynamics in fcc Ni. *Mater. Res. Lett.* **11**, 655–661 (2023).

97. P. Baum, S. Lochbrunner, E. Riedle, Tunable sub-10-fs ultraviolet pulses generated by achromatic frequency doubling. *Opt. Lett.* **29**, 1686–1688 (2004).
